# Enabling Suzuki–Miyaura coupling of Lewis-basic arylboronic esters with a nonprecious metal catalyst†

**DOI:** 10.1039/d2sc03877c

**Published:** 2022-10-21

**Authors:** Michael C. Haibach, Andrew R. Ickes, Sergei Tcyrulnikov, Shashank Shekhar, Sebastien Monfette, Rafal Swiatowiec, Brian J. Kotecki, Jason Wang, Amanda L. Wall, Rodger F. Henry, Eric C. Hansen

**Affiliations:** Process Research and Development, AbbVie Inc. North Chicago Illinois 60064 USA michael.haibach@abbvie.com; Pfizer Chemical Research and Development, Pfizer Inc. Groton Connecticut 06340 USA

## Abstract

The high cost and negative environmental impact of precious metal catalysts has led to increased demand for nonprecious alternatives for widely practiced reactions such as the Suzuki–Miyaura coupling (SMC). Ni-catalyzed versions of this reaction have failed to achieve high reactivity with Lewis-basic arylboron nucleophiles, especially pinacolboron esters. We describe the development of (PPh_2_Me)_2_NiCl_2_ as an inexpensive and air-stable precatalyst that addresses this challenge. Under activation by *n*-BuMgCl, this complex can catalyze the coupling of synthetically important heteroaryl pinacolborons with heteroaryl halides. Mildly basic conditions (aqueous K_3_PO_4_) allow the reaction to tolerate sensitive functional groups that were incompatible with other Ni-SMC methods. Experimental and computational studies suggest that catalyst inhibition by substitution of PPh_2_Me from Ni(ii) intermediates by Lewis basic reactants and products is disfavored relative to more commonly employed ligands in the Ni-SMC, which allows it to operate efficiently in the presence of Lewis bases such as unhindered pyridines.

## Abstract 2

A simple inexpensive ligand enables the Ni-catalyzed Suzuki–Miyaura coupling of heterocyclic and Lewis-basic arylboronic esters under mild conditions.

## Graphical abstract

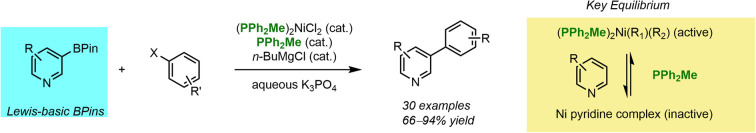

## Introduction

The Pd-catalyzed Suzuki–Miyaura Coupling (Pd-SMC) of aryl halides and arylboron reagents is one of the most practical catalytic methods for C(sp^2^)–C(sp^2^) bond formation.^1–3^ In the pharmaceutical industry, it ranks among the top 2–3 most widely practiced reactions in both medicinal and process chemistry groups.^4,5^ Many ligands have been identified for the Pd-SMC; however, fewer ligands for catalysis by earth-abundant metals (Ni, Cu, Co and Fe) have been developed. Given the increased emphasis on greener and more sustainable catalysis, non-precious metal alternatives for the Pd-SMC are being actively sought.^6^

**Scheme 1:**
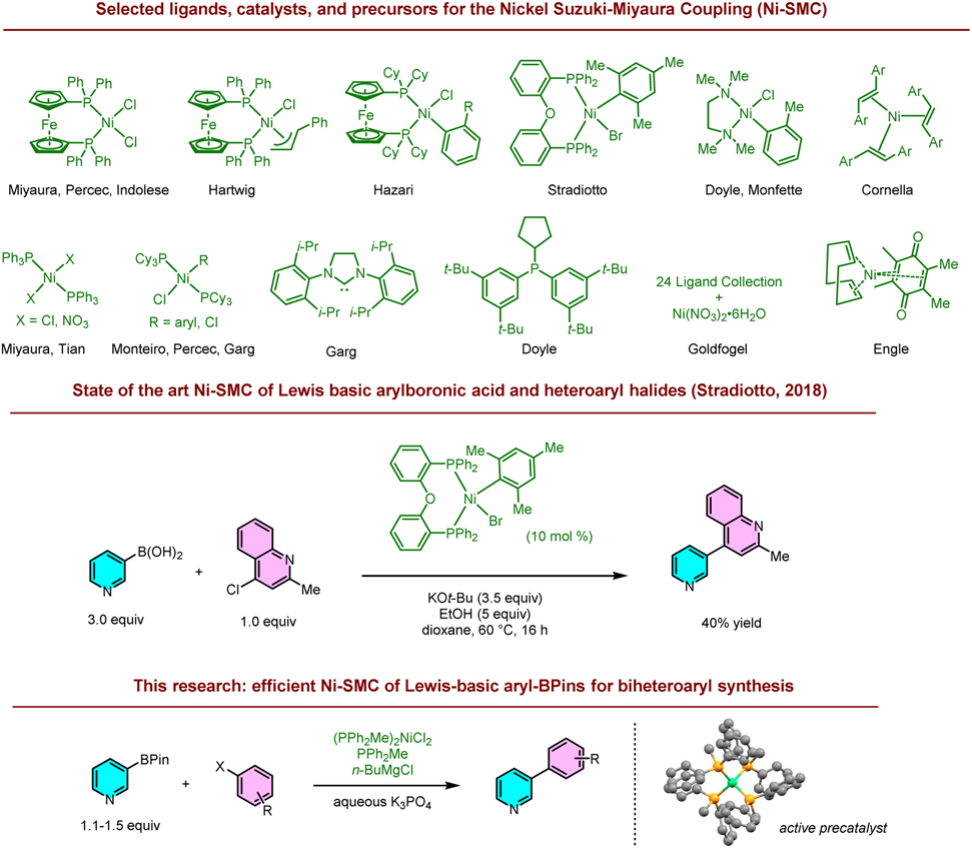
Catalysts for the Ni-SMC and the reaction to prepare Lewis basic heterobiaryls.

The Ni-catalyzed Suzuki–Miyaura Coupling (Ni-SMC), known since the mid-1990s, is a promising alternative to Pd-SMC and even has its advantages (Scheme 1).^7–9^ For example, aryl electrophiles normally unreactive under Pd catalysis, such as carbamates, can be coupled with Ni.^10^ A key challenge to the wider application of the Ni-SMC, however, is the significantly reduced scope of several key heteroarylboron nucleophiles with Ni *vs.* Pd.^11^ Lewis-basic heteroarylborons such as pyridines are particularly problematic for Ni, possibly due to their coordinating ability.^12–14^ Furthermore, aryl BPins were the least reactive of the common SMC nucleophiles examined in a study by Percec.^15^

Complexes of other nonprecious metals besides Ni have also been reported as catalysts for the SMC. Fe^16,17^ and Co-catalyzed^18,19^ couplings of arylboronic esters with alkyl halides have recently been developed. Co catalysts have also been reported for the coupling of arylboronic esters with aryl halides.^20–23^ These examples rely on strong bases (alkyllithiums, lithium amides, or lithium alkoxides) to activate the arylboron reagent, limiting their application with base or nucleophile-sensitive functional groups. An interesting exception to the strong base requirement is the Cu-SMC reported by Giri, which can couple arylboronic esters in the presence of CsF at 120 °C.^24^ In the Fe, Co, and Cu systems, heterocyclic or Lewis-basic arylboron nucleophiles are also not well-precedented.

For these reasons, nonprecious metal catalyzed analogues of the Pd-SMC remain mostly limited to the preparation of biaryl compounds with limited functional groups. The Ni-SMC has rarely been used to prepare heterobiaryls, which are highly relevant in the synthesis of bioactive molecules.^6^ Given our interest in more cost-effective and sustainable catalysts for cross-coupling reactions,^25–27^ the authors at AbbVie and Pfizer desired to develop a general and economical Ni-SMC that addressed these drawbacks. Here we report the identification of a simple inexpensive ligand, PPh_2_Me, that enables the Ni-SMC of an unprecedented range of heterocyclic and Lewis-basic BPins and trifluoroborate salts. The reaction conditions tolerate base-sensitive functional groups such as methyl esters, arylsulfonamides, and unprotected indoles. For several substrates, we observe catalyst performance on par with the best reported Pd catalysts. The origin of the high activity of this catalytic system was also investigated by experimental and computational approaches.

## Results and discussion

### Catalyst optimization

**Scheme 2:**
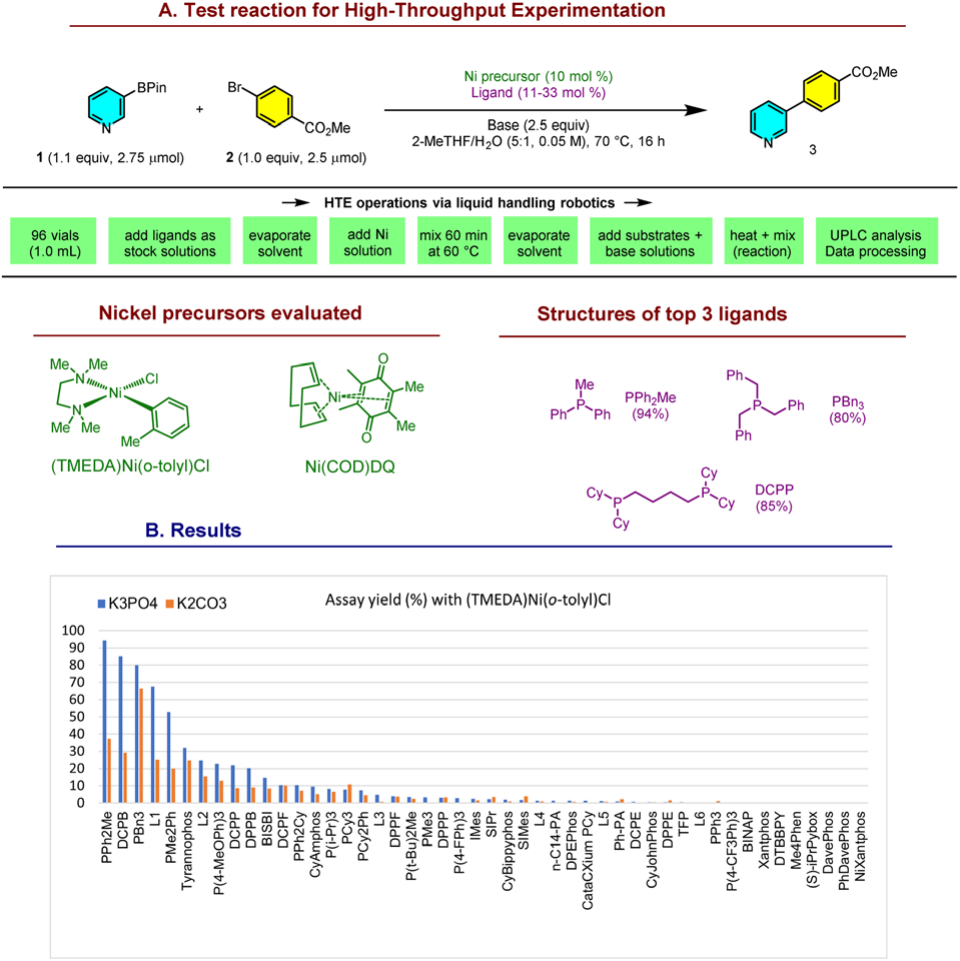
Catalyst discovery by HTE on a bespoke ligand library. See ESI† for structures of all ligands.

At the outset of our research, Ni-SMCs of the important pyridine-3-BPin 1 were unknown with (hetero)aryl halides, and had only been reported in a few cases with specialized electrophiles including vinyl sulfones and amides.^28–30^ The absence of successful precedents and the limited mechanistic understanding of the Ni-SMC of pyridylborons make rational ligand design challenging. Hence, we turned to high-throughput experimentation (HTE)^31–33^ to identify potential leads for a model reaction (Scheme 2).

Screening the coupling of 1 with methyl 4-bromobenzoate 2 against a bespoke library of 48 phosphine, nitrogen, and NHC ligands with (TMEDA)Ni(*o*-tolyl)Cl^34,35^ as the Ni precursor afforded promising leads: PBn_3_ (80% yield), DCPB^36^ (85%) and PPh_2_Me (94%). Other interesting trends were apparent from the data. Reactions with K_3_PO_4_ were typically more effective than K_2_CO_3_, although this difference was smaller with PBn_3_. Commonly applied ligands for the Ni-SMC were ineffective for this coupling, highlighting the challenge posed by Lewis-basic arylborons such as 1 for Ni: DPPF (<5%), DPEPhos (<5%), PCy_3_ (11%), and PPh_3_ (<5%). The same screen was also conducted with Ni(COD)DQ^37^ as the Ni precursor, however this resulted in much lower assay yields (0–17%). We selected the highest yielding, readily available PPh_2_Me for further development.

Based on the reported reactivity of (TMEDA)Ni(*o*-tolyl)Cl,^34,35^ we expected that ligand substitution with PPh_2_Me was initially generating (PPh_2_Me)_2_Ni(*o*-tolyl)Cl in the HTE experiment. Indeed, when used as the catalyst for the coupling of 1 and 2, 3 mol% (PPh_2_Me)_2_Ni(*o*-tolyl)Cl^38,39^ afforded promising conversion (82%) after 16 h (Table 1, entry 2). While both (PPh_2_Me)_2_Ni(*o*-tolyl)Cl and (TMEDA)Ni(*o*-tolyl)Cl are commercially available, they are currently more costly than common Pd sources such as Pd(OAc)_2_ and Pd_2_(dba)_3_. Hence, we pursued the inexpensive and easily prepared (PPh_2_Me)_2_NiCl_2_ (Ni-1) as a catalyst precursor. The catalyst generated from Ni-1, PPh_2_Me and *n*-BuMgCl as an activator afforded 95% conversion of 2 to 3 after 16 h (entry 1). Omitting the *in situ* activation leads to lower conversion and the observation of significant amounts of byproducts (entries 3–4). Zn and *n*-BuLi could also be employed as *in situ* activators^40–42^ of Ni-1, though conversion was slightly lower than with *n*-BuMgCl (entries 5–6).

**Table 1:** Reaction optimization^a^

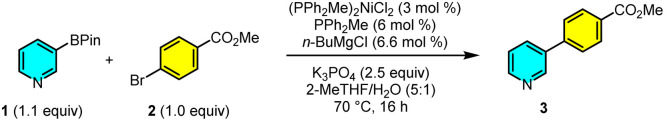		
Entry	Change from conditions shown	%Conv^b^
1	None	95
2	(PPh_2_Me)_2_Ni(*o*-tolyl)Cl (3 mol%) as catalyst	82
3	No added PPh_2_Me	22
4	No added PPh_2_Me, no *n*-BuMgCl	38
5	Zn instead of *n*-BuMgCl	86
6	*n*-BuLi instead of *n*-BuMgCl	86
7	2-MeTHF/H_2_O 4 : 1 v/v	92
8	2-MeTHF/H_2_O 50 : 1 v/v	93
9	2-MeTHF solvent (no added H_2_O)	97
10	Dioxane instead of 2-MeTHF	98
11^c^	*t*-AmOH instead of 2-MeTHF	87
12	Toluene instead of 2-MeTHF	15
13	K_2_HPO_4_ instead of K_3_PO_4_	2
14	K_2_CO_3_ instead of K_3_PO_4_	6
15	1.4 equiv. 1	>99 (91)^d^

a Reactions were conducted on 1.0 mmol scale, 0.4 M in the organic solvent. [Ni-1], PPh_2_Me and *n*-BuMgCl were added to 2-MeTHF prior to addition of water and substrates.

b LC area %conversion to 3, 100 *area product/(total area of product, limiting reagent and relevant impurities).

c Catalyst activation carried out in 2-MeTHF (0.1 v/v *vs. t*-AmOH).

d Isolated yield.

Many reported Ni-SMC methods are either sensitive to adventitious moisture or require careful control of H_2_O stoichiometry. Gratifyingly, the reaction proceeded efficiently with no added H_2_O through 4 : 1 v/v ratio of 2-MeTHF/H_2_O (entries 7–9). We chose 5 : 1 v/v 2-MeTHF/H_2_O for subsequent evaluation due to the slightly higher conversion obtained at this ratio. The conditions with less H_2_O may be useful for specific applications (*i.e.*, a water-sensitive substrate). Dioxane and *t*-AmOH were also effective solvents (entries 10 and 11). Weaker bases K_2_CO_3_ and K_2_HPO_4_ were less effective than K_3_PO_4_ (entries 13 and 14).

### Scope of the reaction

**Scheme 3:**
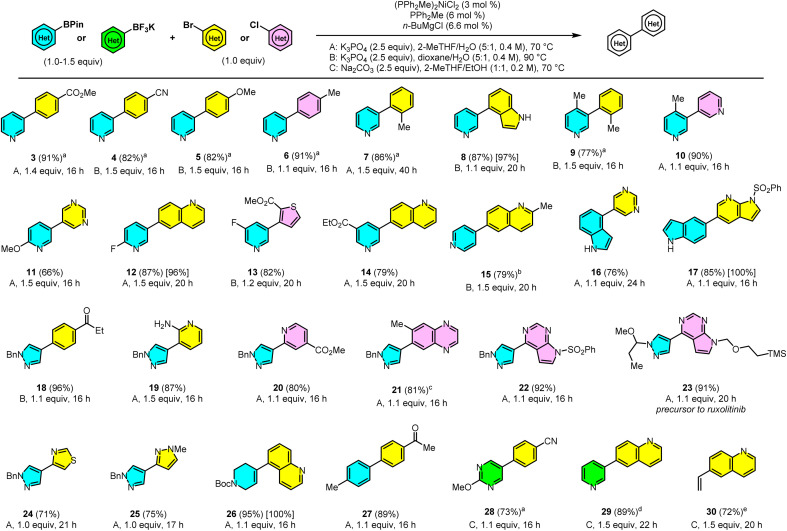
Scope of the Suzuki–Miyaura coupling catalyzed by (PPh_2_Me)_2_NiCl_2_ (Ni-1). Reactions were run on 1.0 mmol scale unless noted, isolated yield in parentheses. Equiv. given in scheme refers to the arylboron reagent. The yield in brackets represents assay yield of reaction with 1 mol% Ni on 1 g scale. (a) Reaction run on 1.0 g scale (b) 5 mol% (PPh_2_Me)_2_NiCl_2_/10 mol% PPh_2_Me/11 mol% *n*-BuMgCl (c) 3 mol% (TMEDA)Ni(*o*-tolyl)Cl/9.9 mol% PPh_2_Me as catalyst (d) 90 °C (e) coupling partner was potassium vinyltrifluoroborate (1.5 equiv.).

With optimized conditions in hand, we explored the scope of the reaction catalyzed by 3 mol% Ni-1 (Scheme 3). The coupling of 1 proceeds efficiently with a range of aryl halides at 70 °C (in 2-MeTHF, bp = 80 °C) or 90 °C (in dioxane, bp = 101 °C). Electron-poor (3 and 4), electron-rich (5 and 6), and *ortho*-substituted (7 and 9) aryl bromides afforded the coupled products in excellent yields. Impressively, an electronically neutral aryl chloride (6), an unprotected bromoindole (8), and Lewis-basic 3-chloropyridine (10) also coupled in high yield.

In addition to the electron-rich pyridine-3-BPin (11), the previously unreported electron-poor pyridine-3-BPins coupled with heteroaryl halides (12–14). Even the less nucleophilic pyridine-4-BPin, an unknown substrate for the Ni-SMC and a challenging one even for the Pd-SMC, coupled efficiently at 90 °C using 5 mol% catalyst (15). A functionalized chlorothiophene was well tolerated as an electrophile (13).

Unprotected indoles are another class of heterocycles previously unreported in the Ni-SMC and were shown to be problematic in Watson's study of DPPF-Ni.^11^ Examples 8, 16 and 17 show the efficient coupling of unprotected indoles as both nucleophile and electrophile components. Compound 17 also demonstrates the viability of a derivatized 7-azaindole as an electrophile, a motif found in several pharmaceuticals.^43^ Methyl/ethyl esters (3, 13, 14, and 20), 2-fluoropyridine (12) and heterocyclic sulfonamides (17 and 22) were well-tolerated, due to the mildly basic conditions of the reaction.

Five-membered Lewis-basic arylborons represent another important class of nucleophiles that is typically difficult for the Ni-SMC. The Ni-SMC of (hetero)aryl halides with pyrazoleborons was previously unreported. Examples 18–25 show the coupling of a pyrazole-4-BPin with functionalized heteroaryl halides at 70 °C, including an unprotected aminopyridine (18). The pyrazolodiazaindole core of several classes of kinase inhibitors was efficiently generated in 22 and 23.^44,45^ Diazaindole 22 is an intermediate in an enantioselective synthesis of ruxolitinib reported by Lin and coworkers at Incyte.^46^ Examples 24 and 25 are notable examples of coupling two five-membered rings, which is typically more challenging than 6-membered rings for both Pd and Ni.^47,48^ An *N*-Boc containing vinyl BPin coupled to form 26 in excellent yield at 70 °C. This type of Suzuki coupling is used extensively in pharma to access 4-arylpiperidine derivatives yet was previously unknown with Ni.^49^ Heteroaryl or Lewis-basic substrates are not required for the catalyst to operate efficiently, as shown by the coupling to form 27.

Excitingly, Ni-1 could also efficiently couple potassium organotrifluoroborates under slightly modified conditions. Both Lewis-basic heteroaryl and vinyl BF_3_K derivatives coupled efficiently at 70–90 °C in the presence of Na_2_CO_3_/EtOH/2-MeTHF^50^ (26–28). These examples further highlight the flexibility of this catalytic system to proceed under mild conditions.

While the scope of the reaction was explored with 3 mol% Ni-1, examples 8, 12, 17 and 26 were also conducted on gram scale with 1 mol% Ni-1/2 mol% PPh_2_Me/2.2 mol% *n*-BuMgCl. The reactions afforded excellent assay yields in 16–24 h without any modification of the general reaction conditions, demonstrating the amenability of this system to large-scale applications. For general couplings of the arylboron nucleophile in 24, the lowest reported catalyst loading with Pd was 1 mol%.^51,52^ In the case of the arylboron in 11, it was 2 mol% with Pd.^53^ Thus, our Ni catalyst can achieve efficiency on par with Pd catalysts for these more challenging substrates! Overall, the scope of the Ni-SMC catalyzed by Ni-1 shows broad Lewis base and functional group tolerance, while in many cases requiring only 1.1 equiv. of the aryl-BPin to afford high yield.

### Characterization of the activated precatalyst

As noted earlier, Ni-1/PPh_2_Me/*n*-BuMgCl showed activity comparable to (PPh_2_Me)_2_Ni(*o*-tolyl)Cl or PPh_2_Me/(TMEDA)Ni(*o*-tolyl)Cl in our optimization studies. NMR and X-ray analysis of Ni-1/PPh_2_Me/*n*-BuMgCl confirmed clean and rapid formation of (PPh_2_Me)_4_Ni, a compound known for over 50 years^54^ but with few prior catalytic applications.^55–57^ Isolated (PPh_2_Me)_4_Ni was found to be a competent precatalyst in the Ni-SMC but had limited stability on the bench, thus *in situ* generation from Ni-1 is preferred.

### Mechanistic studies

We were intrigued by the high activity afforded by the relatively simple PPh_2_Me ligand in the Ni-SMC of Lewis-basic arylboron esters, a reaction where many well-established ligands for Ni did not perform well (see Scheme 2). The most obvious challenge posed by the coupling of substrates such as 1 is their ability to coordinate to Ni, potentially displacing phosphine ligands. Indeed phosphine substitution by Lewis basic amines, including pyridine, was studied by Mizoroki and Nakamura using the related (PPh_3_)_2_Ni(*o*-tolyl)Br complex over forty years ago.^58–60^ Given this precedent, we wondered if the unique ability of (PPh_2_Me)_2_NiCl_2_ in the SMC stems from a reduced tendency of the PPh_2_Me ligand to be substituted by Lewis basic donors.

**Scheme 4:**
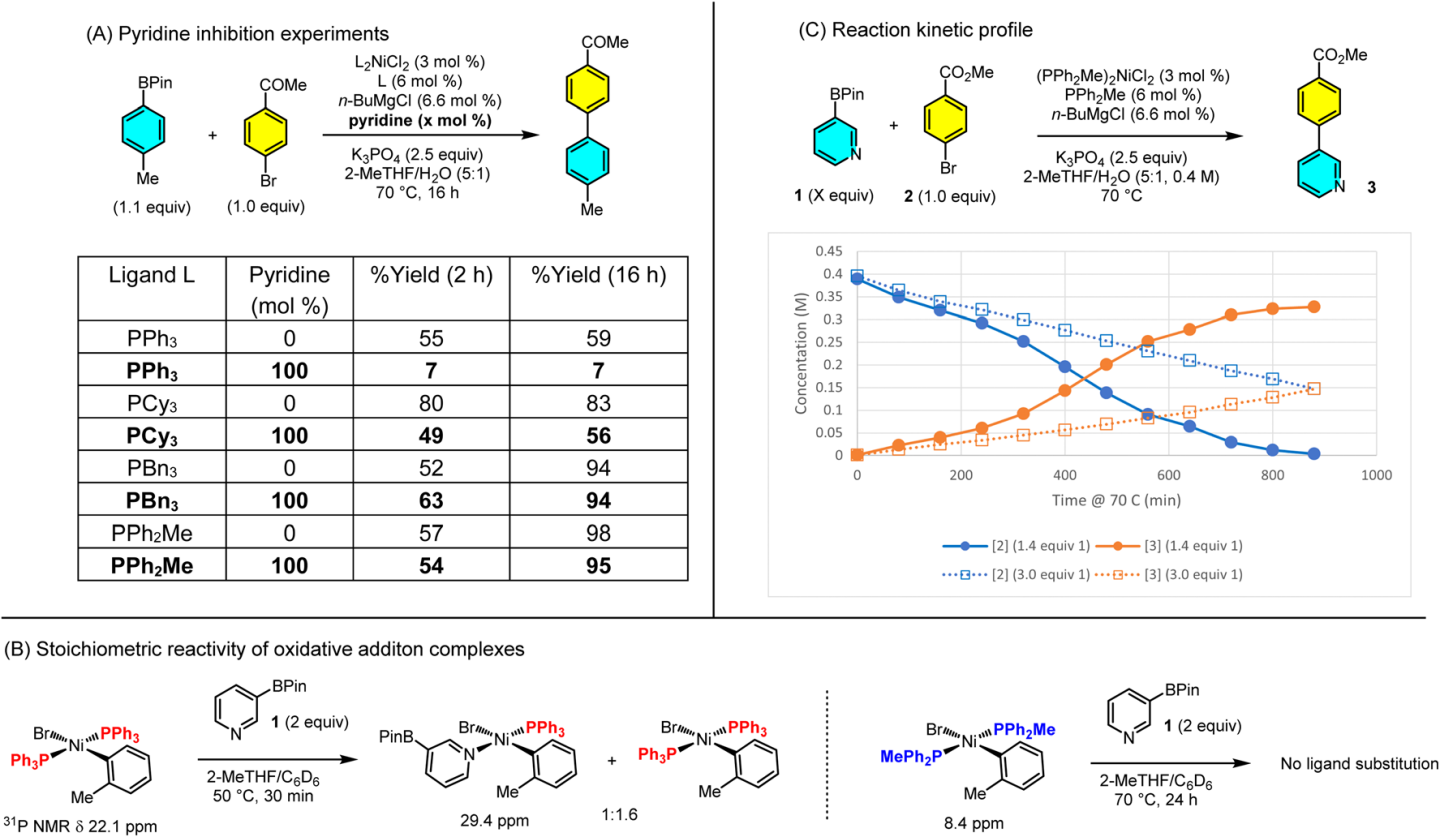
Mechanistic experiments on the role of Lewis bases in the Ni-SMC. ^a^See ESI† for details.

To test this hypothesis, we first investigated the non-heterocyclic Ni-SMC shown in Scheme 4A. With PCy_3_, PPh_3_, PPh_2_Me, or PBn_3_, the reaction proceeded to 59–98% assay yield after 16 h. When 100 mol% pyridine was added, however, the reactions catalyzed by PCy_3_ and PPh_3_ (inactive with 1) were significantly inhibited, as expected, while PPh_2_Me and PBn_3_ (active with 1) were unaffected.^61^ While consistent with our hypothesis that catalyst derived from (PPh_2_Me)_2_NiCl_2_ could be resistant to phosphine displacement, we explored this in more detail through ^31^P NMR studies.

Thus we prepared oxidative addition complexes (PPh_2_Me)_2_Ni(*o*-tolyl)Br and (PPh_3_)_2_Ni(*o*-tolyl)Br to probe the extent of phosphine substitution. We chose these complexes as (1) the ortho substituent renders them isolable as well as air-stable, and (2) 2-bromotoluene is a catalytically relevant aryl halide with PPh_2_Me (Scheme 3, compounds 7 and 9). Complex (PPh_3_)_2_Ni(*o*-tolyl)Br reacted with 2 equiv. 1 at 50 °C to generate a new downfield species in ^31^P NMR that is consistent with previously reported (PPh_3_)(pyridine)Ni(*o*-tolyl)Br complexes (Scheme 4B).^60^ By contrast, heating (PPh_2_Me)_2_Ni(*o*-tolyl)Br with 2 equiv. 1 at 70 °C for 24 h produced no new ^31^P NMR peaks. Hence, substitution of PPh_2_Me by pyridines is disfavored relative to PPh_3_. The extent of substitution of PPh_3_ is significant even with only 2 equiv. of 1 relative to the Ni complex. Under typical catalytic conditions (37–47 equiv. 1 per Ni) the substitution would be expected to proceed much further.^62^ Taken together, these experiments show that addition of a Lewis basic donor such as 1 or pyridine leads to significant substitution of the phosphine ligands, which could lead to off-cycle species and thus slow or inhibit the desired SMC.

**Scheme 5:**
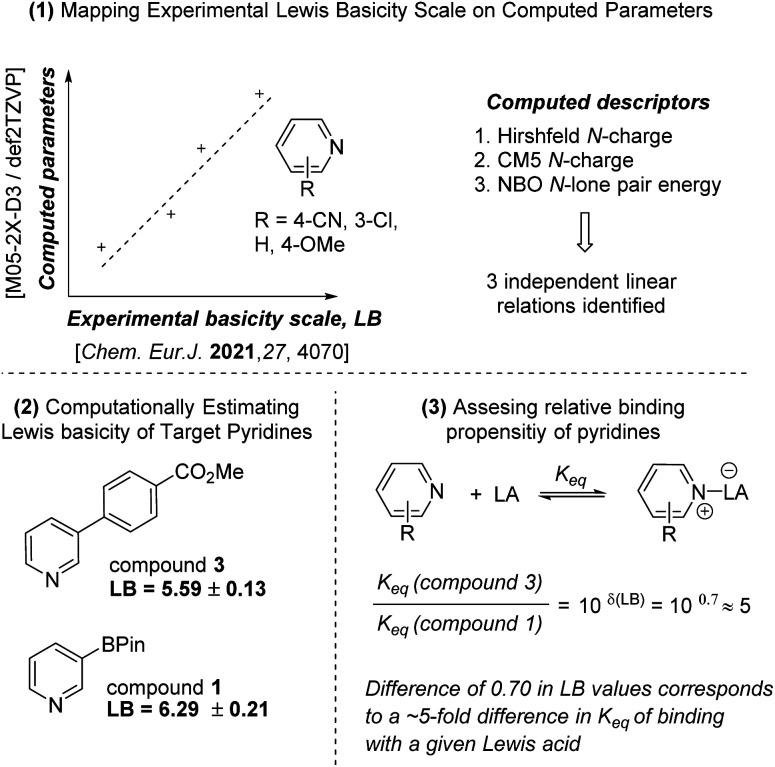
Summary of the procedure used to estimate computed Lewis basicities of pyridines 1 and 3. See ESI† for computational details, calibration of linear models and thermodynamic data.

In Mizoroki and Nakamura's ligand substitution studies, sterics were the primary determinant of substitution equilibrium between PPh_3_ and other ligands. Less-hindered amines and smaller cone angle phosphines displaced PPh_3_ to a greater extent than larger or more hindered ones. When steric effects were held constant, however, higher basicity of the incoming ligand led to greater displacement of PPh_3_. Thus, under typical reaction conditions for the SMC, we should expect a potentially different degree of substitution of the phosphine ligand as BPin 1 is converted into the biaryl product 3. Indeed, the Lewis basicity of 1 and 3, estimated using computed descriptors and experimental values for related pyridines (Scheme 5) show that 1 is 0.70 units more Lewis basic than 3, which corresponds to a 5-fold greater *K*_eq_ in coordinating to a Lewis acid. If correct, we would expect the reaction to show a sigmoidal reaction profile consistent with conversion-dependent relief of inhibition. Thus, we profiled the coupling of 1 and 2 by Ni-1 over time (Scheme 4C) and did observe the expected conversion profile. When the same reaction was conducted with a greater excess 1 (3.0 equiv. *vs.* 1.4 equiv.), the reaction showed overall zero-order behavior over the first 16 h, further evidence of inhibition by 1.^63^ An aliquot of the reaction with 1.4 equiv. 1 at 80 min (5.5% yield) was analyzed by ^31^P NMR, and showed 96% uncoordinated PPh_2_Me, 1% (PPh_2_Me)_4_Ni, and 3% of a PPh_2_Me–Ni complex.^64^

The evidence described above implies that the Lewis basic Ni-SMC is governed by substantial pre-equilibrium steps involving ligand substitution between PPh_2_Me, 1 and 3 (eqn (1)). While under catalytic conditions ([1] > [PPh_2_Me]) most of the equilibrium favors the off-cycle species, enough of the on-cycle intermediates are retained with PPh_2_Me to allow the reaction to proceed efficiently. Indeed, our experiments show that the commonly employed ligands PPh_3_ and PCy_3_ are more susceptible to substitution by pyridines than PPh_2_Me and PBn_3_. Mizoroki and Nakamura's studies imply that this is primarily a steric effect.1
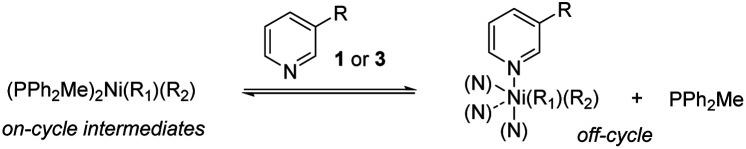


Because they are minor species in eqn (1), the PPh_2_Me–Ni intermediates must also be able to promote very efficient on-cycle steps, including the potentially difficult^65,66^ transmetallation. One implication of this result for future research is to avoid using a large excess of Lewis-basic arylboron reagents when screening Ni-SMCs.

## Conclusions

We have developed an effective and inexpensive catalyst system, *in situ* activated (PPh_2_Me)_2_NiCl_2_, for the Suzuki–Miyaura coupling of Lewis basic heteroaryl BPins and BF_3_Ks with (hetero)aryl bromides and chlorides. The scope and functional group tolerance of the reaction allow important Lewis-basic and biheteroaryl compounds to be prepared with a nonprecious metal catalyst for the first time. Ligand substitution of PPh_2_Me by Lewis basic heterocycles is disfavored relative to more commonly studied phosphines, a key reason for its unique catalytic activity. Future research on the precise balance of steric and electronic factors that contribute to this property should lead to the development of even more highly active ligands for Ni-catalyzed cross couplings of Lewis-basic substrates.

## Author contributions

M. C. H. conceived and directed the study with contributions from S. S. and S. M. M. C. H., A. R. I., and S. S. conducted synthetic experiments. S. T. conducted the computational study. R. S., B. J. K., and J. W. conducted high-throughput experiments. A. L. W. and R. F. H. conducted HRMS and X-ray crystallography, respectively. M. C. H. and S. T. wrote the final draft manuscript. All authors contributed to the data interpretation, manuscript review, and have approved of the final version of the manuscript.

## Conflicts of interest

AbbVie contributed to the design, approval, and execution of this study. M. C. H., A. R. I., S. S., R. S., B. J. K., J. W., A. L. W., and R. F. H. are current or former AbbVie employees and may own AbbVie stocks.

## Supplementary Material

SC-013-D2SC03877C-s001

SC-013-D2SC03877C-s002

SC-013-D2SC03877C-s003
