# Supplementary material for: Enabling Suzuki–Miyaura coupling of Lewis-basic arylboronic esters with a nonprecious metal catalyst
Source: Chem Sci. 2022 Oct 21;13(43):12906–12. doi: 10.1039/d2sc03877c (PMC9645418; doi:10.1039/d2sc03877c)
Supplement: SC-013-D2SC03877C-s001 [file SC-013-D2SC03877C-s001.pdf]

Electronic Supplementary Material (ESI) for Chemical Science.
This journal is © The Royal Society of Chemistry 2022

Supporting Information: Computational Details

Enabling Suzuki-Miyaura coupling of Lewis-basic arylboronic esters with a nonprecious metal catalyst

Michael C. Haibach[1]*, Andrew R. Ickes[1], Sergei Tcyrulnikov[2], Shashank Shekhar[1], Sebastien Monfette[2], Rafal Swiatowiec[1], Brian J. Kotecki[1], Jason Wang[1], Amanda L. Wall[2], Rodger F. Henry[1] and Eric C. Hansen[2]

[1]Process Research and Development, AbbVie, Inc., 1 N Waukegan Road, North Chicago, IL 60064 USA
[2] Pfizer Chemical Research and Development, Pfizer Inc., Groton, Connecticut 06340, USA

Corresponding author email: michael.haibach@abbvie.com

Computational Details

Calculations were performed using Gaussian 16, Revision C.01 software[1]. Optimizations of intermediates and transition states was carried out in the gas phase, using M05-2x functional[2] with Grimme D3 dispersion correction and def2TZVP basis set.[3]. Vibrational frequencies were computed at the specified level of theory to characterize the stationary points as minima (zero imaginary frequencies). All structures were also assessed for conversion using default Gaussian thresholds for maximum force, RMS force, maximum displacement, and RMS displacement. Computations were performed for molecules in their singlet electronic ground states. For a set of pyridines, parameters describing electron density on nitrogen atom were computed at the specified level of theory: Hirshfeld charge[4], CM5 charge[5] and NBO energy of

[1] Gaussian 16, Revision C.01, M. J. Frisch, G. W. Trucks, H. B. Schlegel, G. E. Scuseria, M. A. Robb, J. R. Cheeseman, G. Scalmani, V. Barone, G. A. Petersson, H. Nakatsuji, X. Li, M. Caricato, A. V. Marenich, J. Bloino, B. G. Janesko, R. Gomperts, B. Mennucci, H. P. Hratchian, J. V. Ortiz, A. F. Izmaylov, J. L. Sonnenberg, D. Williams-Young, F. Ding, F. Lipparini, F. Egidi, J. Goings, B. Peng, A. Petrone, T. Henderson, D. Ranasinghe, V. G. Zakrzewski, J. Gao, N. Rega, G. Zheng, W. Liang, M. Hada, M. Ehara, K. Toyota, R. Fukuda, J. Hasegawa, M. Ishida, T. Nakajima, Y. Honda, O. Kitao, H. Nakai, T. Vreven, K. Throssell, J. A. Montgomery, Jr., J. E. Peralta, F. Ogliaro, M. J. Bearpark, J. J. Heyd, E. N. Brothers, K. N. Kudin, V. N. Staroverov, T. A. Keith, R. Kobayashi, J. Normand, K. Raghavachari, A. P. Rendell, J. C. Burant, S. S. Iyengar, J. Tomasi, M. Cossi, J. M. Millam, M. Klene, C. Adamo, R. Cammi, J. W. Ochterski, R. L. Martin, K. Morokuma, O. Farkas, J. B. Foresman, and D. J. Fox, Gaussian, Inc., Wallingford CT, 2019.

[2] Y. Zhao, N. E. Schultz and D. G. Truhlar, Design of Density Functionals by Combining the Method of Constraint Satisfaction with Parametrization for Thermochemistry, Thermochemical Kinetics, and Noncovalent Interactions. *J. Chem. Theory Comput.,* **2006**, *2*, 364–382

[3] Goerigk, L., Hansen, A., Bauer, C., Ehrlich, S., Najibi, A and Grimme, S. A look at the density functional theory zoo with the advanced GMTKN55 database for general main group thermochemistry, kinetics and noncovalent interactions. *Phys. Chem. Chem. Phys.,* **2017**, *19*, 32184-32215

[4] F. L. Hirshfeld, Bonded-atom fragments for describing molecular charge densities, *Theor. Chem. Acc.,* **1977**, *44*, 129-38. DOI: 10.1007/BF00549096

[5] A. V. Marenich, S. V. Jerome, C. J. Cramer and D. G. Truhlar, Charge Model 5: An Extension of Hirshfeld Population Analysis for the Accurate Description of Molecular Interactions in Gaseous and Condensed Phases, *J. Chem. Theory*

lone pair[6]. A set of pyridines included 4 training pyridines and 2 pyridines of interest: product of the reaction and the starting material (in the box on the scheme below):

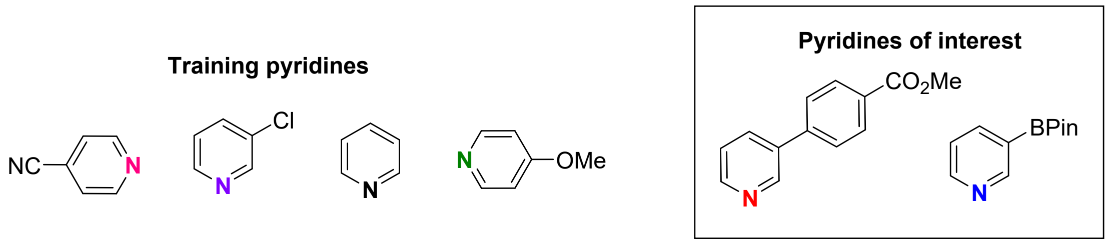

For a 'training set', computed parameters were related to experimentally determined Lewis basicity[7].

	4CN	3Cl	H	4OMe	pdt	SM
Experimental Lewis basicity	3.92	4.83	6.14	7.16	-	-
**Charge on N atom**						
Hirshfeld	-0.146	-0.153	-0.164	-0.181	-0.16	-0.167
CM5	-0.343	-0.35	-0.361	-0.377	-0.357	-0.363
NBO N-LP energy	-0.448	-0.434	-0.422	-0.416	-0.426	-0.417

Graphical representation of obtained trends between computed parameters and experimental Lewis basicity:

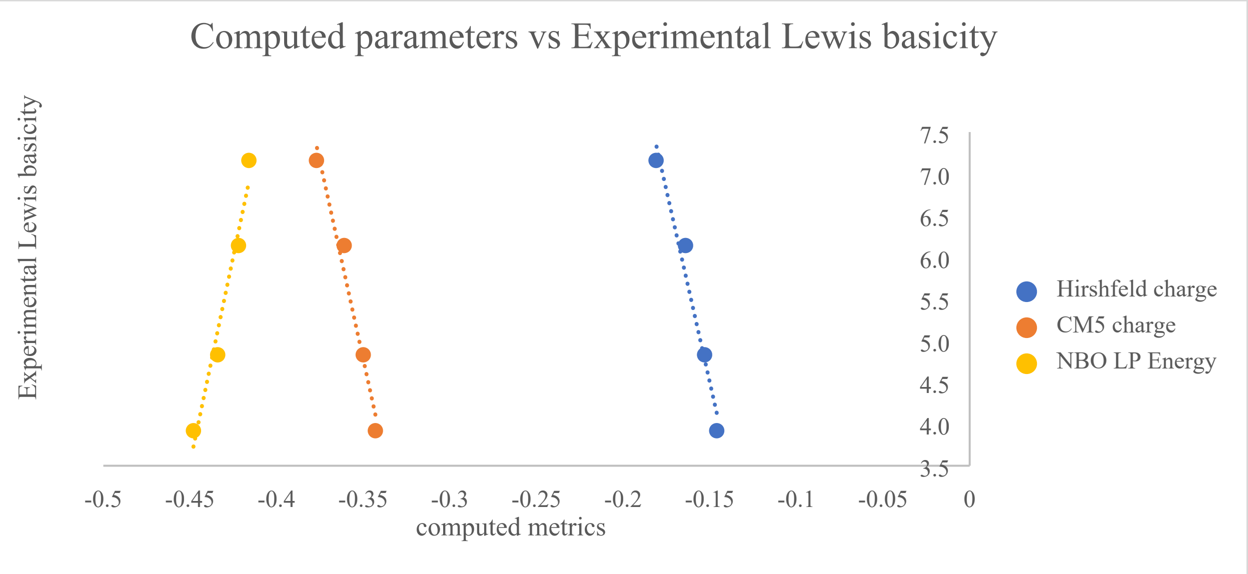

Regression analysis was performed in MS Excel to obtain accurate slope values:

---

and Comput. **2012**, *8*, 527. DOI: 10.1021/ct200866d

[6] J. P. Foster and F. Weinhold, Natural hybrid orbitals, *J. Am. Chem. Soc.,* **1980**, *102*, 7211-18. DOI: 10.1021/ja00544a007

[7] Mayer, R. J., Hampel, N., Ofial, A. R. Lewis Acidic Boranes, Lewis Bases, and Equilibrium Constants: A Reliable Scaffold for a Quantitative Lewis Acidity/Basicity Scale. *Chem. Eur.J.* **2021**,*27*, 4070

Hirshfeld charge vs Lewis basicity

SUMMARY OUTPUT

Regression Statistics	
Multiple R	0.982778934
R Square	0.965854433
Adjusted R Squ	-2
Standard Error	0.322974568
Observations	1

Estimated Lewis basicity of product and SM	
pdt	5.420552
SM	6.064191
delta	0.643639

ANOVA

	df	*SS*	*MS*	*F*	*ignificance F*
Regression	4	5.90125	1.475312	56.5727579	#NUM!
Residual	2	0.208625	0.104313		
Total	6	6.109875			

	Coefficients	*andard Err*	*t Stat*	*P-value*	*Lower 95%*	*Upper 95%*	*Lower 95.0%*	*pper 95.0%*
Intercept							-7.67E-307	7.7E-307
X Variable 1							0	0
X Variable 2	0	0	65535	#NUM!	0	0	0	0
X Variable 3	-9.29119628	1.974801	-4.70488	#NUM!	-17.7881	-0.79431	-17.788081	-0.79431
X Variable 4	-91.9484241	12.22477	-7.52149	0.01722107	-144.547	-39.3495	-144.54735	-39.3495

5

CM5 charge vs Lewis basicity

SUMMARY OUTPUT

Regression Statistics	
Multiple R	0.985665
R Square	0.971535
Adjusted R	-2
Standard E	0.294886
Observatic	1

Estimated Lewis basicity of product and SM	
pdt	5.441306
SM	6.010861
delta	0.569556

ANOVA

	df	*SS*	*MS*	*F*	*ignificance F*
Regressior	4	5.93596	1.48399	68.26271	#NUM!
Residual	2	0.173915	0.086958		
Total	6	6.109875			

	Coefficients	*andard Err*	*t Stat*	*P-value*	*Lower 95%*	*Upper 95%*	*ower 95.0*	*pper 95.0%*
Intercept							0	0
X Variable 1							0	0
X Variable 2							0	0
X Variable	-28.4473	4.112939	-6.91653	0.02027	-46.1438	-10.7507	-46.1438	-10.7507
X Variable	-94.926	11.4893	-8.26213	0.014335	-144.36	-45.4915	-144.36	-45.4915

NBO N LP energy vs Lewis basicity

SUMMARY OUTPUT

Regression Statistics	
Multiple R	0.982379
R Square	0.965069
Adjusted R	-2
Standard E	0.326668
Observatic	1

Estimated Lewis basicity of product and SM	
pdt	5.909033
SM	6.801233

delta	0.8922

ANOVA

	df	*SS*	*MS*	*F*	*ignificance F*
Regressior	4	5.896451	1.474113	55.25566	#NUM!
Residual	2	0.213424	0.106712		
Total	6	6.109875			

	Coefficients	*andard Err*	*t Stat*	*P-value*	*Lower 95%*	*Upper 95%*	*ower 95.0%*	*pper 95.0%*
Intercept							0	0
X Variable 1							0	0
X Variable 2							-5E-306	5.4E-306
X Variable	48.13983	5.736882	8.391289	0.013906	23.45602	72.82364	23.45602	72.82364
X Variable	99.13333	13.33618	7.433415	0.017621	41.7524	156.5143	41.7524	156.5143

Using each of the computed parameters, Lewis basicities of product and starting material were estimated:

Estimated Lewis basicity	Average using 3 models	St dev	st error
Product	5.590296804	0.22554	0.130216
Starting material	6.292095119	0.360673	0.208235
Difference	0.701798315		

Difference between obtained basicity values can be related to relative binding abilities of corresponding pyridines, as can be derived from the original paper.[8]

$$\log\left(K_{eq}\right) = LA_B + LB_B$$

,where LA_b is a Lewis acidity parameter of a given Lewis acid, and LB_b parameter refers to a specific Lewis base. Therefore, for a pair of Lewis bases, we have:

[8] Chem. Eur.J. 2021,27, 4070

$$\log\left(\frac{K_{eq1}}{K_{eq2}}\right) = \log\left(K_{eq1}\right) - \log\left(K_{eq2}\right) = LB_{B1} - LB_{B2}$$

And

$$\frac{K_{eq1}}{K_{eq2}} = 10^{\left(LB_{B1} - LB_{B2}\right)}$$

We estimate that an average difference between computed Lewis basicities (~0.7) corresponds to ~5-fold difference in Keq

$$\frac{K_{eq1}}{K_{eq2}} = 10^{(0.7)} = 5.01$$

As can be seen from the graphics below, performed analysis is interpolative as the training set pf pyridines covers a wide range of computed parameters and experimental basicities.

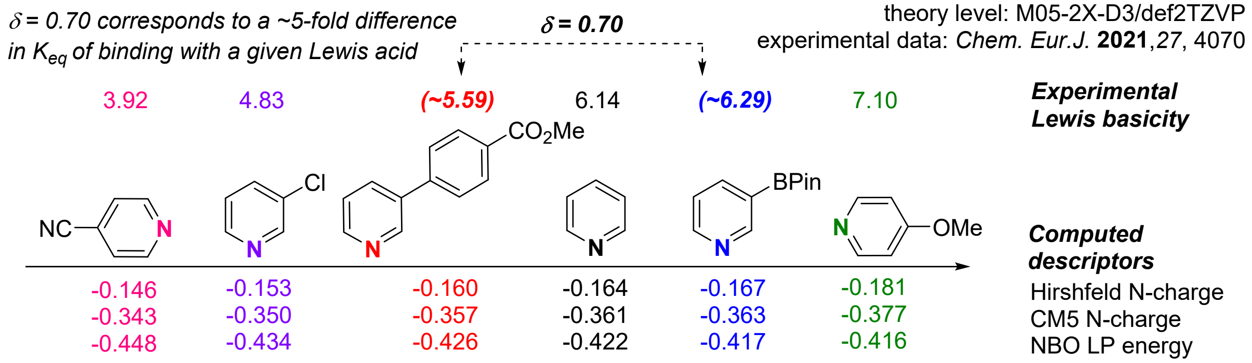

Coordinates and Thermochemical Data

4CN pyridine
Electronic Energy (EE) -340.61514
Zero-point Energy Correction 0.089044
Thermal Correction to Energy 0.094973
Thermal Correction to Enthalpy 0.095917
Thermal Correction to Free Energy 0.058853
EE + Zero-point Energy -340.5261
EE + Thermal Energy Correction -340.52017
EE + Thermal Enthalpy Correction -340.51922
EE + Thermal Free Energy Correction -340.55629

C	1.49549300	1.13683400	-0.00014500
C	1.49544700	-1.13683500	0.00010900
C	0.11169600	1.19784300	-0.00012400
H	2.07720100	2.04789200	-0.00023300
C	0.11156600	-1.19777300	0.00012100
H	2.07701300	-2.04799800	0.00018400
C	-0.59017600	0.00005400	0.00000700
H	-0.40561000	2.14351000	-0.00021000
N	2.18101200	-0.00007500	-0.00002600
H	-0.40582200	-2.14338100	0.00022000
C	-2.02610200	-0.00018300	0.00002400
N	-3.17106000	0.00012300	0.00003800

3Cl pyridine
Electronic Energy (EE) -707.9544
Zero-point Energy Correction 0.080848
Thermal Correction to Energy 0.086141
Thermal Correction to Enthalpy 0.087086
Thermal Correction to Free Energy 0.051187
EE + Zero-point Energy -707.87355
EE + Thermal Energy Correction -707.86826
EE + Thermal Enthalpy Correction -707.86731
EE + Thermal Free Energy Correction -707.90321

C	-2.19993800	-0.09596700	0.00039900
C	-0.20169100	-1.18214000	-0.00024000
C	-1.58847900	1.14829300	0.00016100
H	-3.27788100	-0.17628000	0.00024000
C	0.49214300	0.01878600	-0.00006300
H	0.33972400	-2.11777300	-0.00014400
C	-0.20620000	1.21102800	0.00006200
H	-2.18226700	2.04878900	0.00014400
H	0.32143600	2.15199300	0.00003600
N	-1.52519900	-1.23953100	0.00007600
Cl	2.21766900	0.00999900	-0.00016000

4OMe pyridine
Electronic Energy (EE) -362.90675
Zero-point Energy Correction 0.12398
Thermal Correction to Energy 0.130579
Thermal Correction to Enthalpy 0.131523
Thermal Correction to Free Energy 0.093131
EE + Zero-point Energy -362.78277

EE + Thermal Energy Correction -362.77618
EE + Thermal Enthalpy Correction -362.77523
EE + Thermal Free Energy Correction -362.81362

C	1.33987800	1.29065200	-0.00002700
C	1.85201800	-0.90962100	0.00012800
C	-0.02665300	1.04162600	-0.00014000
H	1.68803200	2.31517900	0.00000900
C	0.52460200	-1.28178000	-0.00001300
H	2.62127500	-1.66983800	0.00021200
C	-0.44416300	-0.28270000	-0.00020000
H	-0.71886900	1.86627700	-0.00016600
N	2.27566800	0.35451700	0.00008700
H	0.22537000	-2.31768300	0.00000600
C	-2.73093600	0.32330800	0.00023300
H	-3.67928400	-0.19995800	0.00058400
H	-2.65321600	0.94448700	0.89155600
H	-2.65390000	0.94453700	-0.89111400
O	-1.73094400	-0.68169200	-0.00019700

pyridine

Electronic Energy (EE) -248.34691
Zero-point Energy Correction 0.090498
Thermal Correction to Energy 0.094716
Thermal Correction to Enthalpy 0.09566
Thermal Correction to Free Energy 0.063138
EE + Zero-point Energy -248.25641
EE + Thermal Energy Correction -248.25219
EE + Thermal Enthalpy Correction -248.25125
EE + Thermal Free Energy Correction -248.28377

C	-1.13495600	-0.71744500	-0.00037200
C	1.13447900	-0.71818000	0.00040200
C	-1.18999800	0.66834900	0.00014500
H	-2.04701300	-1.29912500	0.00063300
C	1.19040700	0.66762400	-0.00019400
H	2.04621400	-1.30036200	-0.00003800
C	0.00045500	1.37495800	-0.00005900
H	-2.14288700	1.17412700	0.00047400
H	2.14365700	1.17274300	-0.00021100
H	0.00078900	2.45473500	0.00018900
N	-0.00044100	-1.40770800	-0.00008300

product

Electronic Energy (EE) -707.41976
Zero-point Energy Correction 0.216787
Thermal Correction to Energy 0.229901
Thermal Correction to Enthalpy 0.230846
Thermal Correction to Free Energy 0.175748
EE + Zero-point Energy -707.20297
EE + Thermal Energy Correction -707.18986
EE + Thermal Enthalpy Correction -707.18891
EE + Thermal Free Energy Correction -707.24401

C	-3.11351400	0.99753800	-0.52784600
C	-5.06948600	-0.06196400	-0.07537600
C	-2.34710500	-0.03740800	0.00594900
H	-2.62129800	1.85961500	-0.95887800
C	-4.41083400	-1.15247000	0.47182500
H	-6.14975200	-0.03817800	-0.11715200
C	-3.02927100	-1.13753500	0.51262400
H	-4.97141200	-1.98628300	0.86443400
H	-2.48150200	-1.95791800	0.95305300
N	-4.43800900	0.99586100	-0.57080900
C	-0.87389500	0.03978100	0.02131100
C	-0.10414800	-1.08639300	-0.26463800
C	-0.22925100	1.23930500	0.32107100
C	1.27683500	-1.01983400	-0.25164400
H	-0.59353800	-2.01345800	-0.52368900
C	1.15050900	1.31020300	0.33336900
H	-0.81484700	2.11157600	0.56962200
C	1.90797400	0.18146500	0.04757900
H	1.86846600	-1.89123300	-0.48070900
H	1.66119900	2.23037100	0.57100700
C	3.38683700	0.31265400	0.07540700
O	4.01397600	-0.83385200	-0.21441400
O	3.97038200	1.33296900	0.32930100
C	5.44365800	-0.75762400	-0.20230900
H	5.78233900	-0.03141700	-0.93451600
H	5.79080800	-0.46147700	0.78256700
H	5.79088000	-1.75186200	-0.45310700

Starting material

Electronic Energy (EE) -659.16087
Zero-point Energy Correction 0.264994
Thermal Correction to Energy 0.278974
Thermal Correction to Enthalpy 0.279918
Thermal Correction to Free Energy 0.224591
EE + Zero-point Energy -658.89587
EE + Thermal Energy Correction -658.88189
EE + Thermal Enthalpy Correction -658.88095
EE + Thermal Free Energy Correction -658.93628

C	2.41010900	-1.16052600	0.18233500
C	4.39924900	-0.08634800	0.01330800
C	1.68124000	0.01333700	-0.00110000
H	1.88514400	-2.09550600	0.32816800
C	3.78473100	1.14246000	-0.18006700
H	5.47883100	-0.15857900	0.02452200
C	2.40289100	1.18904000	-0.18587800
H	4.38019100	2.03111300	-0.32030500
H	1.87810500	2.12248200	-0.33119700
N	3.73632400	-1.22402700	0.19258300
C	-1.99502900	-0.77105800	-0.09291200
C	-1.99758800	0.77323300	0.09213400
B	0.13179500	0.00354500	0.00060800
O	-0.62769300	1.12225600	-0.23153400
O	-0.62473100	-1.11576300	0.23255300
C	-2.93311600	1.52099600	-0.83260100
H	-3.95977600	1.19320400	-0.67250100
H	-2.87498000	2.58568900	-0.61854100
H	-2.66836700	1.36339800	-1.87319200
C	-2.22258800	-1.19240400	-1.53641600
H	-1.99893700	-2.25277400	-1.62449800
H	-3.25477100	-1.02494200	-1.83696600
H	-1.56454600	-0.64402200	-2.20785400
C	-2.92921200	-1.52189400	0.83068300
H	-3.95685900	-1.19784900	0.66912800
H	-2.86660600	-2.58630500	0.61681600
H	-2.66622500	-1.36358200	1.87160600
C	-2.22840200	1.19352000	1.53539700
H	-2.00949600	2.25489700	1.62410600
H	-3.26019900	1.02174900	1.83476200
H	-1.56907200	0.64740000	2.20739000
