# Supplementary material for: Enabling Suzuki–Miyaura coupling of Lewis-basic arylboronic esters with a nonprecious metal catalyst
Source: Chem Sci. 2022 Oct 21;13(43):12906–12. doi: 10.1039/d2sc03877c (PMC9645418; doi:10.1039/d2sc03877c)
Supplement: SC-013-D2SC03877C-s002 [file SC-013-D2SC03877C-s002.pdf]

Electronic Supplementary Material (ESI) for Chemical Science.
This journal is © The Royal Society of Chemistry 2022

Supporting Information: Experimental Details

Enabling Suzuki-Miyaura coupling of Lewis-basic arylboronic esters with a nonprecious metal catalyst

Michael C. Haibach[1]*, Andrew R. Ickes[1], Sergei Tcyrulnikov[2], Shashank Shekhar[1], Sebastien Monfette[2], Rafal Swiatowiec[1], Brian J. Kotecki[1], Jason Wang[1], Amanda L. Wall[2], Rodger F. Henry[1] and Eric C. Hansen[2]

[1]Process Research and Development, AbbVie, Inc., 1 N Waukegan Road, North Chicago, IL 60064 USA
[2] Pfizer Chemical Research and Development, Pfizer Inc., Groton, Connecticut 06340, USA

Corresponding author email: michael.haibach@abbvie.com

Table of Contents

General Information	2
Catalyst Syntheses	3
HTE Information	4
Control Experiments on Trace Pd	6
Pyridine Inhibition Study	7
Stoichiometric Reactivity of Ni Oxidative Addition Complexes	8
General Procedures	9
Determination of Assay Yields by Response Factor	10
Determination of Assay Yields by Internal Standard	11
Kinetics Experiments	12
Characterization of Products	14
References	20
NMR Spectra	22

General Information

All catalytic reactions were carried out under inert atmosphere. Solvents and liquid reagents were sparged with nitrogen or argon prior to use. Small-scale catalytic reactions were conducted with glass vials and PTFE stir bars, which were disposed of after one use. Commercially available reagents were used as received. All ligands and catalysts were obtained commercially unless otherwise noted and stored under N_2. Flash chromatography was performed on a Teledyne Isco system with silica cartridges, using Celite to dry-load substrates. All previously reported products of catalytic reactions were characterized by ^{1}H and ^{13}C NMR, MS, and compared with literature data. Previously unreported compounds were additionally characterized by HRMS.

Instrument frequencies for the ^{1}H, ^{13}C, and ^{31}P NMR spectra are given in the characterization data. ^{1}H and ^{13}C spectra were referenced to the residual proteo (^{1}H) or deuterated (^{13}C) solvent solvent signal. ^{31}P spectra were referenced to an external standard of 85% H_3PO_4. NMR spectra were processed with MNova. HPLC analyses were performed using acetonitrile and water with 0.1% $HClO_4$, 0.1% H_3PO_4 or 0.1% HCOOH as eluents. WD-XRF analysis was conducted on a Rigaku Primus III+ WD-XRF instrument using a calibrated method (Pharmpak_Pd) provided by Rigaku. ICP-MS analysis was conducted by Intertek Pharmaceutical Services (Whitehouse, NJ). Routine MS analyses were performed with an APCI instrument. HRMS samples were dissolved in a solution of 1:1 ACN:H_2O with an added lock mass, then loop injected into a Thermo Scientific LTQ Orbitrap instrument in positive mode HESI.

Scheme S1: Catalyst syntheses

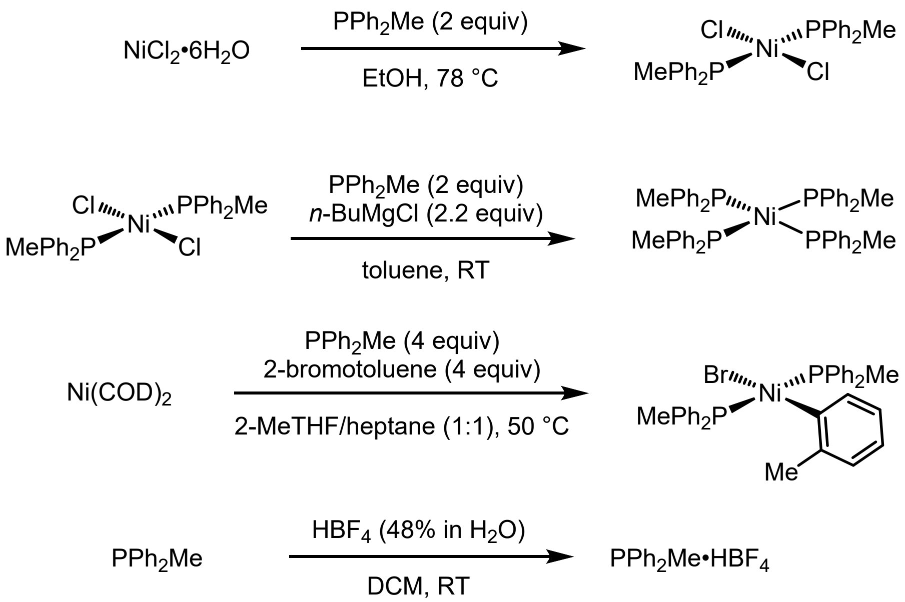

($(PPh_2Me)_2NiCl_2$ (**[Ni-1]**) was prepared according to a literature procedure.[1] Layering a saturated THF solution of *trans*-$(PPh_2Me)_2NiCl_2$ with EtOH resulted in the formation of large, maroon-colored crystals suitable for X-Ray analysis after 48 h. The CIF was deposited at the CCDC, deposition number 2109558.

$(PPh_2Me)_4Ni$: A 20 mL vial was charged with $(PPh_2Me)_2NiCl_2$ (500 mg, 0.943 mmol, 1.00 equiv), toluene (10 mL) and PPh_2Me (378 mg, 1.887 mmol, 2.00 equiv). The resulting deep red slurry was mixed and *n*-BuMgCl (1.04 mL of a 2 M solution in THF, 2.075 mmol, 2.20 equiv) was added dropwise. During the addition, gas evolution was observed. Near the end of the addition, the reaction mixture changed from a deep red slurry to a cloudy orange solution. The solution was stirred for an additional 5 min after the Grignard addition was complete. The solution was filtered through a 0.45 µm PTFE syringe filter and concentrated in vacuo to give $(PPh_2Me)_4Ni$ as an orange solid (793 mg, 0.922 mmol, 98% yield). X-Ray quality crystals were obtained by layering a saturated toluene solution of $(PPh_2Me)_4Ni$ with n-heptane at ambient temperature for 48 h. ^1H NMR (400 MHz, C_6D_6) δ 7.28 – 7.21 (m, 16H), 7.07 – 6.93 (m, 32H), 1.71 (s, 12H). ^{31}P NMR (162 MHz, C_6D_6) δ 3.94. Spectroscopic data were consistent with the literature reports.[2, 3] The CIF was deposited at the CCDC, deposition number 2109557.

$(PPh_2Me)_2Ni$(*o*-tolyl)Br: A 2-dram vial was charged with $Ni(COD)_2$ (100 mg, 0.36 mmol, 1.00 equiv), *n*-heptane (2 mL), 2-MeTHF (2 mL), 2-bromotoluene (249 mg, 1.45 mmol, 4.00 equiv), and PPh_2Me (291 mg, 1.45 mmol, 4.00 equiv). The reaction mixture was stirred at 50 °C for 16 h, then cooled to ambient temperature and concentrated on a rotovap to give an orange oil. This was crystallized twice from *i*-PrOH/*n*-heptane to give $(PPh_2Me)_2Ni$(*o*-tolyl)Br as a yellow solid (63 mg, 0.10 mmol, 28% yield). ^1H NMR (400 MHz, C_6D_6) δ 7.78 (dtd, *J* = 7.2, 5.1, 2.4 Hz, 4H), 7.61 (ddt, *J* = 9.5, 4.8, 2.5 Hz, 4H), 7.13 (s, 4H), 7.08 – 6.98 (m, 8H), 7.01 – 6.91 (m, 2H), 6.64 (td, *J* = 7.1, 1.5 Hz, 1H), 6.54 (ddd, *J* = 7.0, 5.5, 1.8 Hz, 2H), 2.73 (s, 3H), 1.10 (t, *J* = 3.7 Hz, 6H). ^{31}P NMR (162 MHz, C_6D_6) δ 8.54. This compound was prepared previously.[4, 5]

$(PPh_3)_2Ni$(*o*-tolyl)Br was prepared according to a literature procedure.[6]

PPh_2Me-HBF_4: A 250 mL round bottom flask was charged with 70 mL CH_2Cl_2 and sparged with N_2 for 20 min. PPh_2Me (5 mL, 26.9 mmol) was added to the flask in one portion and the mixture stirred. 48% aqueous HBF_4 was added slowly, and the resulting biphasic mixture was stirred vigorously at ambient temperature for 2 h.[7] The reaction mixture was transferred to a separatory funnel (no longer air-sensitive). The lower layer was collected, dried over $MgSO_4$, filtered, and concentrated on a rotovap to give a clear, colorless oil. The resulting oil rapidly solidified to give a white solid, which was crushed with a spatula to give a white powder (7.37 g, 25.6 mmol, 95% yield). ^1H NMR (400 MHz, $CDCl_3$) δ 8.15 (d, J = 264 Hz, 1 H) 7.86 – 7.72 (m, 6H), 7.66 (tdd, J = 7.3, 3.5, 2.3 Hz, 4H), 7.49 (s, 1H), 2.52 (d, J = 15.2 Hz, 3H). ^{31}P NMR (162 MHz, $CDCl_3$) δ 2.39. Spectroscopic data were consistent with the literature report.[8]

Experimental Procedure for High-Throughput Screening Experiments

Inside a nitrogen-filled glovebox, 1 mL glass tubes in an aluminum block were charged with a 2-MeTHF solution of ligands (0.275–0.825 µmol, 0.11–0.33 equiv). The solvent was removed in vacuo. Next, (TMEDA)Ni(*o*-tolyl)Cl or Ni(COD)DQ (0.075 mg, 0.25 µmol, 0.10 equiv) was dispensed as a solution in 2-MeTHF to vials containing ligands. DIPEA was added to the vials containing protonated ligand precursors (1 equiv DIPEA per proton). A glass bead was charged to each tube and the tubes were sealed. The tubes were sealed and mixed at 300 RPM on an orbital mixer for 60 min at 60 °C, then the solvent was removed in vacuo using a Genevac centrifugal evaporator inside the glovebox. Next, 3-(4,4,5,5-tetramethyl-1,3,2-dioxaborolan-2-yl)pyridine (0.564 mg, 2.75 µmol, 1.10 equiv), methyl 4-bromobenzoate (0.538 mg, 2.5 µmol, 1.00 equiv) were dispensed as a solution in 2-MeTHF (100 µL) to each tube, followed by a solution of K_3PO_4 (1.327 mg, 6.25 µmol, 2.50 equiv) in H_2O (20 µL). The tubes were sealed and agitated at 300 RPM on an orbital mixer at 70 °C for 16 h. After cooling to ambient temperature, the vials were diluted with acetonitrile containing 4,4'-dimethylbiphenyl as an internal standard, filtered, and analyzed by UPLC-MS.

Scheme S2: Structures of Catalysts from HTS Experiment and Ligand to Metal Ratios (L/M)

Alkylphosphines: L/M = 2.2

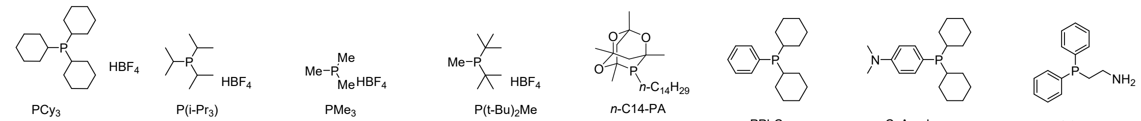

PCy$_3$ P(i-Pr$_3$) PMe$_3$ P(t-Bu)$_2$Me *n*-C14-PA PPhCy$_2$ CyAmphos L6

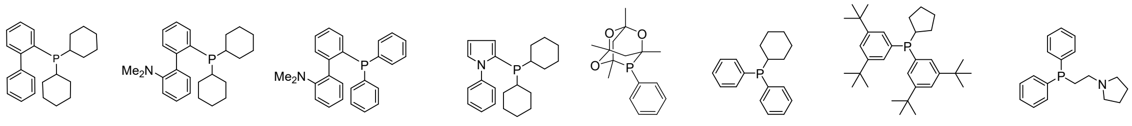

CyJohnphos Davephos PhDavephos CataCXium PCy Ph-PA PPh$_2$Cy Tyrranophos L1

Triarylphosphines: L/M = 3.3

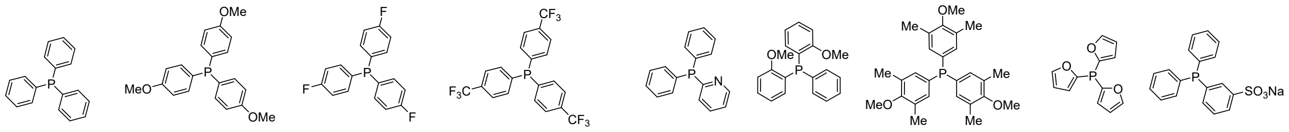

PPh$_3$ P(p-MeOPh)$_3$ P(p-FPh)$_3$ P(p-CF$_3$Ph)$_3$ L4 L5 L2 TFP L3

Bidentate and Tridentate Ligands: L/M = 1.1

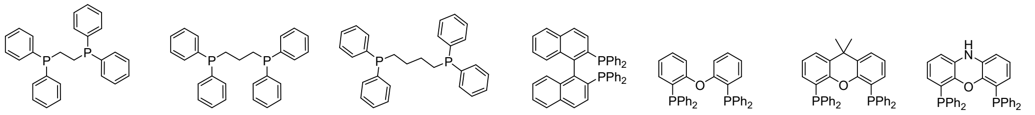

DPPE DPPP DPPB BINAP DPEPhos Xantphos NiXantphos

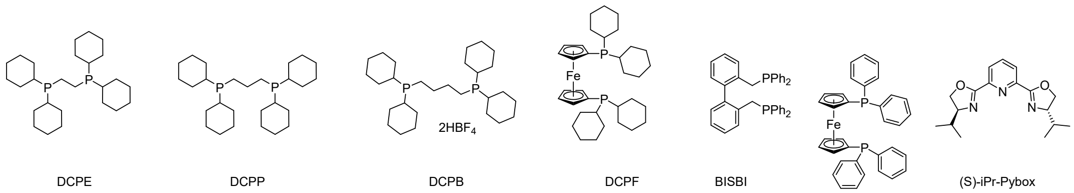

DCPE DCPP DCPB DCPF BISBI (S)-iPr-Pybox

DPPF

Others L/M = 2.2

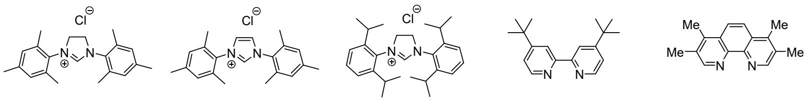

SIMes IMes SIPr DTBPY Me$_4$Phen

Control Experiments: Catalysis by Trace Pd

Several avenues exist for possible contamination of experiments by Pd, as some previous publications on "metal-free" or "Pd-free" versions of C-N and C-C couplings have shown. Pd-catalyzed borylation of aryl halides can be used to produce aryl pinacol boronates, so the arylborons used in this study could be a source of Pd. In some accounts, trace Pd was found to be present in inorganic bases such as Na_2CO_3. Hypothetically, residual Pd could also be present in the Ni salt used to prepare $(PPh_2Me)_2NiCl_2$. To evaluate the likelihood of trace Pd causing the catalytic activity observed in this study, we conducted the following additional experiments.[9]

Scheme S3: Analysis of trace Pd in starting materials and control experiments

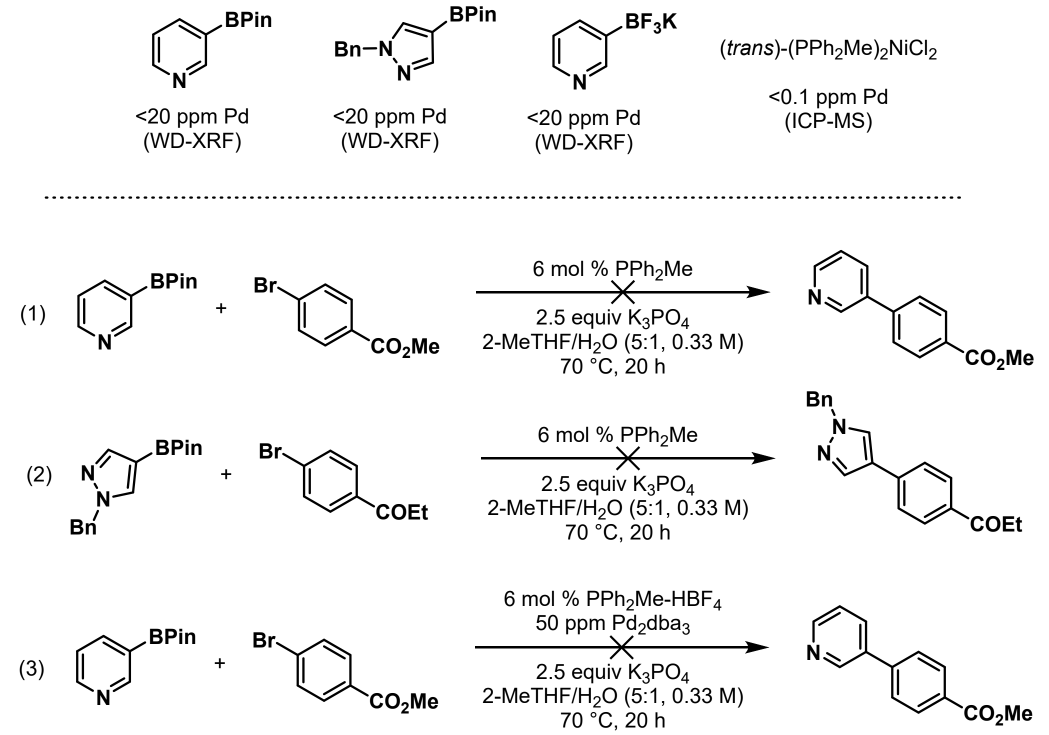

Three heterorarylborons used in this study were analyzed for Pd content by a calibrated XRF method with a practical quantification limit of 20 ppm (Scheme S3). All the compounds were found to contain <20 ppm Pd. While Suzuki couplings catalyzed by <20 ppm Pd have been reported, they are typically under more forceful conditions (i.e., >120 °C).[10] A sample of the $(PPh_2Me)_2NiCl_2$ used in our study was analyzed by ICP-MS and found to contain <0.1 ppm Pd.

We conducted control reactions without $(PPh_2Me)_2NiCl_2$ and *n*-BuMgCl (Figure S3, equations 1 and 2). In both cases, no formation of the Suzuki coupled product could be detected. Finally, when 50 ppm $Pd_2(dba)_3$ was added in place of Ni (Equation 3), no coupled product was detected. This argues against enough trace Pd being present in the reagents, base, or solvent to result in the observed catalytic activity in this study.

Pyridine inhibition experiment

Table S1: Effective of added pyridine on PPh_3, PCy_3, PBn_3 and PPh_2Me derived catalysts

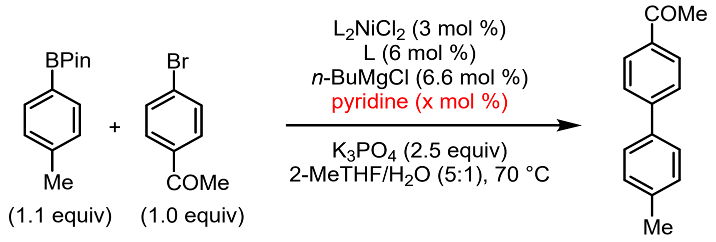

Entry	Ligand L	Pyridine (mol %)	%Yield (2 h)	%Yield (16 h)
1	PPh_3	0	55	59
2	PPh_3	100	7	7
3	PCy_3	0	80	83
4	PCy_3	100	49	56
5	PBn_3	0	52	94
6	PBn_3	100	63	94
7	PPh_2Me	0	57	98
8	PPh_2Me	100	54	95

Reactions were conducted on 1.0 mmol scale according to the general procedure. Yields were determined by HPLC using biphenyl as an internal standard.

Stoichiometric reactivity of Ni oxidative addition complexes

$(PPh_3)_2Ni($*o*-tolyl$)Br$: A 4 mL vial was charged with $(PPh_3)_2Ni$(*o*-tolyl)Cl (27 mg, 0.036 mmol, 1.0 equiv), 3-(4,4,5,5-tetramethyl-1,3,2-dioxaborolan-2-yl)pyridine (15 mg, 0.072 mmol) and 2-MeTHF (1.0 mL). Tricyclohexylphosphine oxide (4.6 mg, 0.016 mmol) was added as an internal standard and 0.1 mL C_6D_6 was added for locking. The reaction was stirred at 50 °C for 30 min, then 31P NMR of an aliquot was recorded. The starting material (22.11 ppm) was present along with a new peak (29.40 ppm) in a 1.6:1 ratio.

$(PPh_2Me)_2Ni($*o*-tolyl$)Br$: A 4 mL vial was charged with $(PPh_2Me)_2Ni$(*o*-tolyl)Cl (27 mg, 0.036 mmol, 1.0 equiv), 3-(4,4,5,5-tetramethyl-1,3,2-dioxaborolan-2-yl)pyridine (15 mg, 0.072 mmol) and 2-MeTHF (1.0 mL). Tricyclohexylphosphine oxide (4.3 mg, 0.015 mmol) was added as an internal standard and 0.1 mL C_6D_6 was added for locking. The reaction was stirred at 50 °C and analyzed by ^{31}P NMR. No new peaks were detected after 30 min or 24 h. The same reaction was repeated at 70 °C for 24 h, and no new ^{31}P NMR were observed.

General Procedures for Suzuki-Miyaura Coupling Catalyzed by $(PPh_2Me)_2NiCl_2$

General Procedure A

A vial was charged with $(PPh_2Me)_2NiCl_2$ (0.03 mmol, 3 mol %), 2-MeTHF (2.5 mL) and PPh_2Me (0.06 mmol, 6 mol %) and the resulting deep red solution was stirred. *n*-BuMgCl (2 M in THF, 0.066 mmol, 6.6 mol %) was added dropwise, resulting in a yellow solution. This solution was transferred to a vial containing the aryl pinacol boronate (1.0–1.5 mmol), aryl halide (if solid, 1.0 mmol), and K_3PO_4 (2.5 mmol). Next, aryl halide (if liquid, 1.0 mmol) and H_2O (0.5 mL) were added. The reaction mixture was stirred at 70 °C for 16-24 h, then cooled to ambient temperature. The reaction mixture was diluted with 20 mL EtOAc and washed with H_2O (20 mL x 2) and 20 wt% NaCl (20 mL). The combined aqueous layers were extracted with EtOAc (20 mL). The combined organic layers were concentrated in vacuo and the crude product was purified by flash chromatography.

General Procedure B

A vial was charged with $(PPh_2Me)_2NiCl_2$ (0.03 mmol, 3 mol %), dioxane (2.5 mL) and PPh_2Me (0.06 mmol, 6 mol %) and the resulting deep red solution was stirred. *n*-BuMgCl (2 M in THF, 0.066 mmol, 6.6 mol %) was added dropwise, resulting in a yellow solution. This solution was transferred to a vial containing the aryl pinacol boronate (1.0–1.5 mmol), aryl halide (if solid, 1.0 mmol), and K_3PO_4 (2.5 mmol). Next, aryl halide (if liquid, 1.0 mmol) and H_2O (0.5 mL) were added. The reaction mixture was stirred at 90 °C for 16-24 h, then cooled to ambient temperature. The reaction mixture was diluted with 20 mL EtOAc and washed with H_2O (20 mL x 2) and 20 wt% NaCl (20 mL). The combined aqueous layers were extracted with EtOAc (20 mL). The combined organic layers were concentrated in vacuo and the crude product was purified by flash chromatography.

General Procedure C

A vial was charged with $(PPh_2Me)_2NiCl_2$ (0.03 mmol, 3 mol %), 2-MeTHF (2.5 mL) and PPh_2Me (0.06 mmol, 6 mol %) and the resulting deep red solution was stirred. *n*-BuMgCl (2 M in THF, 0.066 mmol, 6.6 mol %) was added dropwise, resulting in a yellow solution. This solution was transferred to a vial containing the aryl pinacol boronate (1.0–1.5 mmol), aryl halide (if solid, 1.0 mmol), and Na_2CO_3 (2.5 mmol). Next, aryl halide (if liquid, 1.0 mmol) and EtOH (2.5 mL) were added. The reaction mixture was stirred at 70 °C for 16-24 h, then cooled to ambient temperature. The reaction mixture was diluted with 20 mL EtOAc and filtered through a Celite plug, eluting with an additional 20 mL EtOAc. The filtrate was concentrated in vacuo and the crude product was purified by flash chromatography.

Determination of reaction assay yields using product response factor (Scheme 3, 1 mol % Ni examples)

After the reaction was complete according to HPLC analysis, the bottom aqueous layer was removed. The upper organic layer was diluted with EtOAc until homogenous, then weighed (wt_{soln}). An aliquot of the organic layer was weighed (wt_{sample}) into a tared volumetric flask, which was then filled to the line with ACN (vol_{sample}). This solution was analyzed by HPLC to obtain an area response (A_{sample}) for a given injection volume (I_{sample}).

Separately, a standard of the product was weighed (wt_{std}) into a volumetric flask and filled to the line with ACN (vol_{std}). This solution was analyzed by HPLC to obtain an area response (A_{std}) for a given injection volume (I_{std}). In the examples analyzed, $I_{sample} = I_{std}$.

The weight percent of product in the organic layer (wt%) and reaction assay yield was then calculated as follows:

$$wt\% = \frac{A_{sample} \times vol_{sample} \times wt_{std} \times 100}{A_{std} \times vol_{std} \times wt_{sample}}$$

$$reaction\ assay\ yield\ (\%) = \frac{wt\% \times wt_{soln} \times 100}{theoretical\ yield}$$

Substrate	A soln (mAu)	vol soln (ml)	weight sample (mg)	area std	vol std (ml)	weight std (mg)	wt%	wt soln (g)	theor. yield (g)	yield (%)
8	2530.9	50	137.0	3173.4	50	5.2	3.03	31.700	0.991	96.8
12	484.6	100	166.0	5327.2	25	24.9	5.46	19.034	1.078	96.4
17	313.6	100	75.0	2885.9	25	17.7	10.26	10.814	1.107	100.2
26	653.7	100	150.0	4234.8	25	25.9	10.66	14.061	1.492	100.5

Determination of reaction assay yields of using an internal standard (Scheme 2, Table S2, Kinetics Experiments)

An internal standard (biphenyl, Sigma-Aldrich, >99% or 4,4'-dimethylbiphenyl, Combi-Blocks, 98%) and product standard were weighed into a volumetric flask and diluted to a known volume. This solution was analyzed by HPLC to give a wt/wt relative response factor RRf:

$$RRf = \frac{Rf\ standard}{Rf\ product} = \frac{area\ standard}{area\ product} * \frac{wt\ product}{wt\ standard}$$

Note that in the above equation, the injection volume and dilution volume terms cancel due to the method of solution preparation. The reaction mixture was sampled and analyzed by HPLC. The reaction assay yield was then calculated as follows:

$$Reaction\ assay\ (mg) = RRf * \frac{wt\ standard\ (mg) * area\ product}{area\ standard}$$

$$\%Assay\ yield = \frac{Reaction\ assay\ (mg)}{Theor.\ yield\ (mg)} * 100$$

Procedure for kinetics experiment

Inside a N_2 glovebox, a 100 mL cylindrical overhead stirred reactor (Mettler-Toledo EasyMax) was charged with methyl 4-bromobenzoate **2** (5.00 g, 23.25 mmol, 1.00 equiv), 3-(4,4,5,5-tetramethyl-1,3,2-dioxaborolan-2-yl)pyridine **1** (6.68 g, 32.60 mmol, 1.40 equiv), K_3PO_4 (12.34 g, 58.10 mmol, 2.5 equiv), and 4,4'-dimethylbiphenyl (2.00 g). The reactor was fitted with a downward impellor style stir blade, an internal temperature probe, an air-cooled condensor and a Mettler-Toledo EasySampler probe.

A solution of Ni catalyst was prepared by adding *n*-BuMgCl (0.77 mL of 2 M solution in THF, 1.54 mmol, 6.6 mol %) dropwise to a stirred slurry of $(PPh_2Me)_2NiCl_2$ (370 mg, 0.689 mmol, 3 mol %) and PPh2Me (279 mg, 1.395 mmol, 6 mol %) in 10 mL 2-MeTHF. Upon addition of the Grignard, the maroon slurry became a yellow-orange solution.

The reactor was charged with 48.7 mL 2-MeTHF and mixed at 300 rpm. The catalyst solution was then charged, followed by 11.7 mL H_2O. The initial reaction concentration was 0.40 M **2** in 2-MeTHF. A slight temperature increase was observed upon addition of H_2O due to dissolution of the K_3PO_4.

The reactor internal temperature was adjusted to 70 °C (ramp time approximately 20 min), then the reaction was periodically sampled by the Easysampler probe. Samples were automatically diluted 200x with ACN/H_2O 4:1, and the reaction was backfilled with a continuously N_2 sparged solution of 2-MeTHF. The samples remained homogenous and were analyzed by HPLC or UPLC-MS.

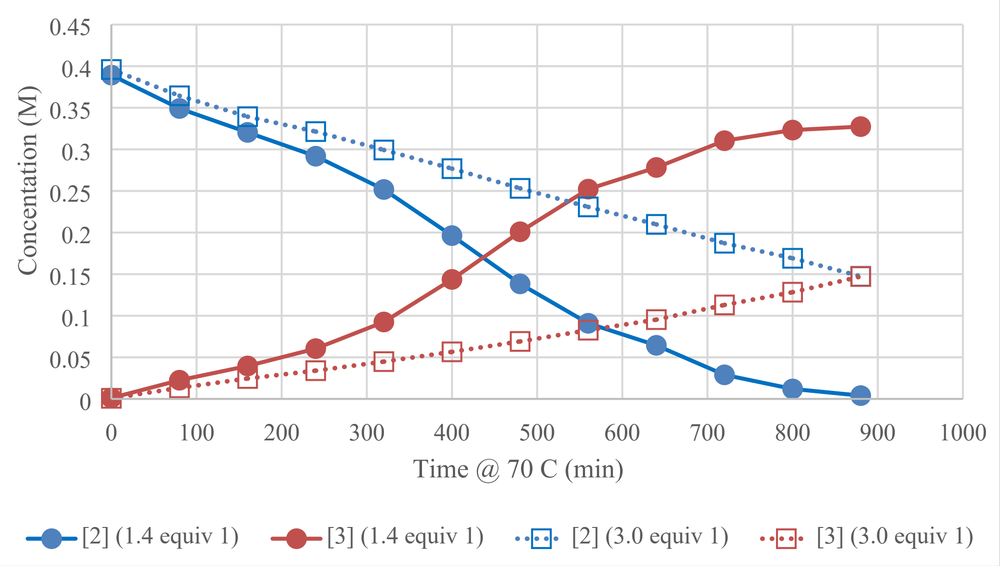

Kinetics and catalyst resting state analysis of a non-Lewis basic coupling

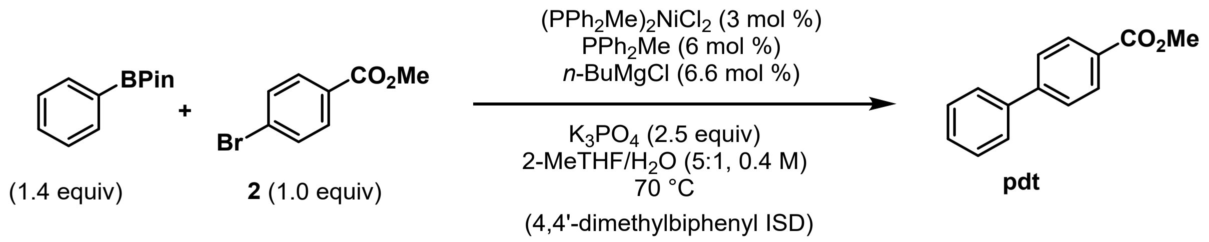

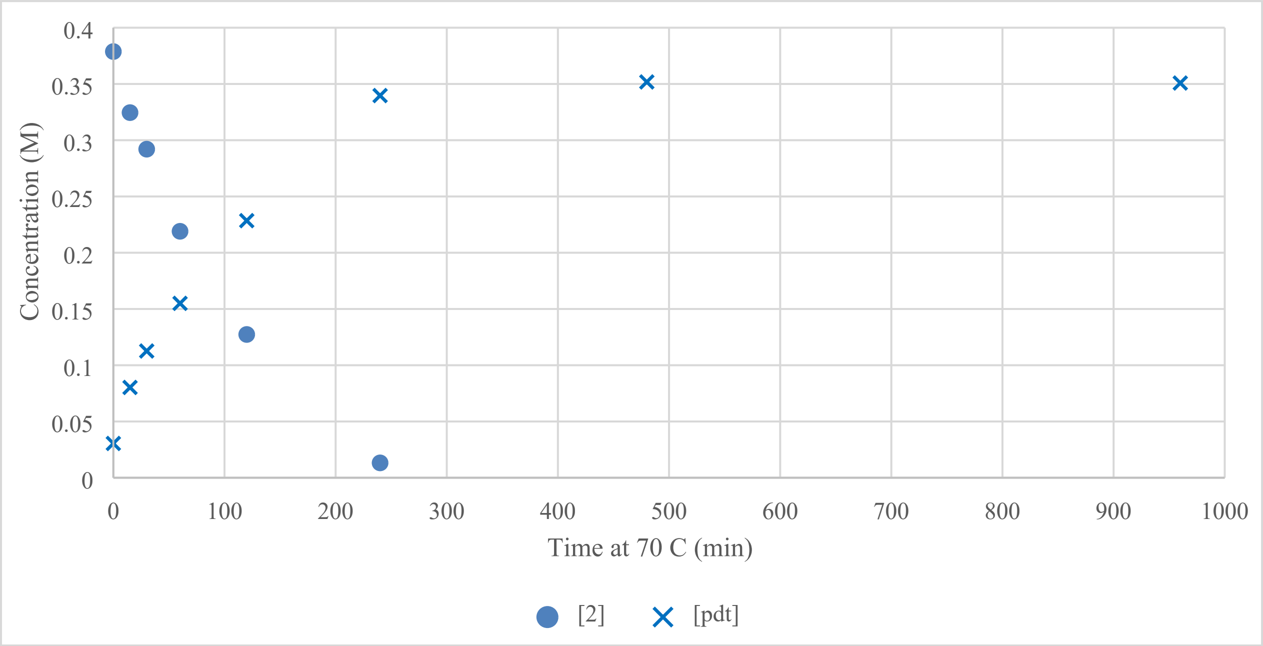

NMR analysis

At 1 h and 16 h, a sample of the stirred reaction mixture (0.4 mL) was analyzed by ^{31}P NMR with 0.1 mL C_6D_6 added for locking. At 1 h, the following species were present: 14.09 ppm (37%), $(PPh_2Me)_4Ni$, 3.39 ppm (15%), PPh_2Me, -27.35 ppm (48%). At 16 h, the following species were present: 5.59 ppm, 12%, $(PPh_2Me)_4Ni$, 3.39 ppm (71%), -5.58 ppm (3%), -27.37 ppm, PPh_2Me (14%). PPh_2Me oxide (reported 29.93 ppm in $CDCl_3$[11]) was not observed.

Characterization of products

Methyl 4-(pyridin-3-yl)benzoate (3)[12]: Prepared according to general procedure A on from methyl 4-bromobenzoate (1.00 equiv, 1.00 g, 4.65 mmol) and 3-(4,4,5,5-tetramethyl-1,3,2-dioxaborolan-2-yl)pyridine (1, 1.40 equiv). 16 h reaction time. Isolated 904 mg as a white solid (4.24 mmol, 91% yield). ^{1}H NMR (400 MHz, $CDCl_3$) δ 8.83 (dd, *J* = 2.4, 0.9 Hz, 1H), 8.59 (dd, *J* = 4.8, 1.6 Hz, 1H), 8.12 – 8.05 (m, 2H), 7.85 (ddd, *J* = 8.0, 2.4, 1.7 Hz, 1H), 7.63 – 7.56 (m, 2H), 7.33 (ddd, *J* = 7.9, 4.8, 0.9 Hz, 1H), 3.89 (s, 3H). ^{13}C NMR (101 MHz, $CDCl_3$) δ 166.66, 149.25, 148.32, 142.17, 135.49, 134.47, 130.34, 129.74, 127.06, 123.65, 52.22. MS (m/z) 214.2 $(M+H)^+$

4-(pyridin-3-yl)benzonitrile (4)[13]: Prepared according to general procedure B on 5.49 mmol scale from 4-bromobenzonitrile (1.00 equiv, 1.00 g, 5.49 mmol) and 3-(4,4,5,5-tetramethyl-1,3,2-dioxaborolan-2-yl)pyridine (1.50 equiv). 16 h reaction time. Isolated as 815 mg as a white solid (4.52 mmol, 82% yield). ^{1}H NMR (400 MHz, $CDCl_3$) δ 8.82 (dd, *J* = 2.4, 0.9 Hz, 1H), 8.63 (dd, *J* = 4.8, 1.6 Hz, 1H), 7.86 (ddd, *J* = 7.9, 2.4, 1.6 Hz, 1H), 7.77 – 7.70 (m, 2H), 7.70 – 7.62 (m, 2H), 7.39 (ddd, *J* = 8.0, 4.9, 0.9 Hz, 1H). ^{13}C NMR (101 MHz, $CDCl_3$) δ 149.75, 148.23, 142.31, 134.75, 134.50, 132.87, 127.80, 123.81, 118.57, 111.91. MS (m/z) 181.2 $(M+H)^+$

3-(4-methoxyphenyl)pyridine (5)[12]: Prepared according to general procedure B from 4-bromoanisole (1.00 g, 5.35 mmol, 1.00 equiv) and 3-(4,4,5,5-tetramethyl-1,3,2-dioxaborolan-2-yl)pyridine (1.645 g, 8.02 mmol, 1.50 equiv). 16 h reaction time. Isolated 812 mg (4.38 mmol, 82% yield) as a white solid. ^{1}H NMR (400 MHz, $CDCl_3$) δ 8.81 (d, *J* = 1.5 Hz, 1H), 8.54 (dd, *J* = 4.8, 1.6 Hz, 1H), 7.86 – 7.79 (m, 1H), 7.55 – 7.49 (m, 2H), 7.37 – 7.29 (m, 1H), 7.05 – 6.98 (m, 2H), 3.86 (s, 3H) ppm. ^{13}C NMR (101 MHz, $CDCl_3$) δ 159.90, 148.15, 148.02, 136.39, 133.99, 130.40, 128.36, 123.63, 114.70, 55.52 ppm. MS (m/z) 186.2 $(M+H)^+$

3-(p-tolyl)pyridine (6)[14]: Prepared according to general procedure B from 4-chlorotoluene (1.00 equiv, 127 mg, 1.00 mmol) and 3-(4,4,5,5-tetramethyl-1,3,2-dioxaborolan-2-yl)pyridine (226 mg, 1.10 mmol, 1.10 equiv). 16 h reaction time. Isolated 154 mg (0.91 mmol, 91% yield) as a colorless oil. ^{1}H NMR (400 MHz, $CDCl_3$) δ 8.84 (d, *J* = 2.3 Hz, 1H), 8.56 (dd, *J* = 4.8, 1.7 Hz, 1H), 7.83 (dt, *J* = 7.9, 2.0 Hz, 1H), 7.47 (d, *J* = 8.1 Hz, 2H), 7.32 (dd, *J* = 7.9, 4.8 Hz, 1H), 7.28 (d, *J* = 7.8 Hz, 2H), 2.40 (s, ^{3}H). ^{13}C NMR (101 MHz, $CDCl_3$) δ 148.24, 148.23, 138.03, 136.56, 134.96, 134.11, 129.82, 126.99, 123.52, 21.16. MS (m/z) 170.2 $(M+H)^+$

3-(o-tolyl)pyridine (7)[15]: Prepared according to general procedure A from 2-bromotoluene (1.00 equiv, 1.00 g, 5.85 mmol) and 3-(4,4,5,5-tetramethyl-1,3,2-dioxaborolan-2-yl)pyridine (1.50 equiv). 16 h reaction time. 852 mg (5.03 mmol, 86% yield) isolated as colorless oil. ^{1}H NMR (400 MHz, $CDCl_3$) δ 8.61 – 8.57 (m, 2H), 7.65 – 7.61 (ddd, 1H), 7.35 – 7.18 (m, 5H), 2.27 (s, 3H) ppm. ^{13}C NMR (176 MHz, $CDCl_3$) δ 149.98, 148.14, 138.10, 137.43, 136.41, 135.55, 129.85, 128.10, 126.08, 122.97, 20.34 ppm. MS (m/z) 170.2 $(M+H)^+$

4-(pyridin-3-yl)-1H-indole (8)[16]: Prepared according to general procedure B from 4-bromoindole (1.00 equiv, 196 mg, 1.00 mmol) and 3-(4,4,5,5-tetramethyl-1,3,2-dioxaborolan-2-yl)pyridine (1.10 equiv). 16 h reaction time. 169 mg (0.87 mmol, 87% yield) isolated as white

solid. ^{1}H NMR (400 MHz, DMSO-d_6) δ 11.35 (s, 1H), 8.87 (dd, *J* = 2.4, 0.9 Hz, 1H), 8.58 (dd, *J* = 4.8, 1.6 Hz, 1H), 8.06 (dt, *J* = 7.9, 2.0 Hz, 1H), 7.56 – 7.44 (m, 3H), 7.22 (t, *J* = 7.6 Hz, 1H), 7.14 (dd, *J* = 7.3, 1.0 Hz, 1H), 6.55 (ddd, *J* = 3.1, 2.0, 1.0 Hz, 1H). ^{13}C NMR (101 MHz, DMSO-d_6) δ 148.79, 147.91, 136.48, 136.37, 135.47, 129.41, 126.35, 125.57, 123.76, 121.44, 118.89, 111.58, 99.69. MS (m/z) 195.2 $(M+H)^+$

4-methyl-3-(o-tolyl)pyridine (9)[17]: Prepared according to general procedure B from 2-bromotoluene (1.00 g, 5.85 mmol, 1.00 equiv) and 4-methyl-3-(4,4,5,5-tetramethyl-1,3,2-dioxaborolan-2-yl)pyridine (1.50 equiv). 16 h reaction time. Isolated 820 mg (4.47 mmol, 77% yield) as a white solid. ^{1}H NMR (400 MHz, $CDCl_3$) δ 8.46 (d, *J* = 5.0 Hz, 1H), 8.34 (s, 1H), 7.34 – 7.22 (m, 3H), 7.20 (d, *J* = 5.0 Hz, 1H), 7.09 (d, *J* = 7.0 Hz, 1H), 2.09 (s, 3H), 2.06 (s, 3H) ppm. ^{13}C NMR (101 MHz, $CDCl_3$) δ 149.88, 148.51, 145.26, 137.57, 137.53, 136.24,130.18, 129.64, 128.08, 125.90, 124.89, 19.92, 19.38 ppm. MS (m/z) 184.2 $(M+H)^+$

4-methyl-3,3'-bipyridine (10)[18]: Prepared according to general procedure A from 4-methyl-3-(4,4,5,5-tetramethyl-1,3,2-dioxaborolan-2-yl)pyridine (241 mg, 1.10 mmol, 1.10 equiv) and 3-chloropyridine (114 mg, 1.00 mmol, 1.00 equiv). 16 h reaction time. Isolated 154 mg (0.91 mmol, 91% yield) as a colorless oil. ^{1}H NMR (400 MHz, $CDCl_3$) δ 8.65 (dd, *J* = 4.9, 1.7 Hz, 1H), 8.60 (dd, *J* = 2.4, 0.9 Hz, 1H), 8.49 (d, *J* = 5.1 Hz, 1H), 8.43 (s, 1H), 7.66 (ddd, *J* = 7.9, 2.3, 1.7 Hz, 1H), 7.39 (ddd, *J* = 7.8, 4.9, 0.9 Hz, 1H), 7.22 (dt, *J* = 5.0, 0.8 Hz, 1H), 2.29 (s, 3H). ^{13}C NMR (101 MHz, $CDCl_3$) δ 149.84, 149.12, 148.93, 144.85, 136.53, 134.10, 133.67, 125.39, 123.34, 19.80. MS (m/z) 171.1 $(M+H)^+$

5-(6-methoxypyridin-3-yl)pyrimidine (11)[19]: Prepared according to general procedure A from 5-bromopyrimidine (159 mg, 1.00 mmol, 1.00 equiv) and 2-methoxy-5-(4,4,5,5-tetramethyl-1,3,2-dioxaborolan-2-yl)pyridine (1.50 equiv). 16 h reaction time. Isolated 124 mg (0.66 mmol, 66% yield) as a white solid. ^{1}H NMR (400 MHz, $CDCl_3$) δ 9.22 (s, 1H), 8.92 (s, 2H), 8.40 (d, *J* = 2.6 Hz, 1H), 7.79 (dd, *J* = 8.6, 2.6 Hz, 1H), 6.91 (d, *J* = 8.6 Hz, 1H), 4.01 (s, 3H) ppm. ^{13}C NMR (176 MHz, $CDCl_3$) δ 164.88, 157.71, 154.58, 145.33, 137.14, 131.70, 123.47, 111.90, 53.94. MS (m/z) 188.2 $(M+H)^+$

6-(6-fluoropyridin-3-yl)quinoline (12): Prepared according to general procedure A from 6-bromoquinoline (208 mg, 1.00 mmol, 1.00 equiv) and 2-fluoro-5-(4,4,5,5-tetramethyl-1,3,2-dioxaborolan-2-yl)pyridine (1.50 equiv). 20 h reaction time. Isolated 196 mg (0.87 mmol, 87% yield) as a white solid. ^{1}H NMR (400 MHz, $CDCl_3$) δ 8.96 (dd, *J* = 4.3, 1.7 Hz, 1H), 8.58 – 8.53 (m, 1H), 8.27 – 8.19 (m, 2H), 8.10 (ddd, *J* = 8.8, 7.6, 2.6 Hz, 1H), 7.98 (d, *J* = 2.1 Hz, 1H), 7.90 (dd, *J* = 8.8, 2.1 Hz, 1H), 7.47 (dd, *J* = 8.3, 4.2 Hz, 1H), 7.07 (dd, *J* = 8.5, 3.0 Hz, 1H). ^{13}C NMR (101 MHz, $CDCl_3$) δ 163.51 (d, J = 240.2 Hz), 151.11, 147.95, 146.37 (d, J = 15.0 Hz), 140.14 (d, J = 8.0 Hz), 136.42, 135.00, 134.27, 134.22, 128.74, 128.58, 125.97, 122.03, 109.86 (d, J = 37.4 Hz). ^{19}F NMR (376 MHz, $CDCl_3$) δ -70.58. HRMS (m/z) $(M+H)^+$: calculated 225.08225, found 225.08210.

Methyl 3-(5-fluoropyridin-3-yl)thiophene-2-carboxylate (13): Prepared according to general procedure B from methyl 3-chlorothiophene-2-carboxylate (177 mg, 1.00 mmol, 1.00 equiv) and 3-fluoro-5-(4,4,5,5-tetramethyl-1,3,2-dioxaborolan-2-yl)pyridine (1.20 equiv). 20 h reaction time. Isolated 194 mg (0.82 mg, 82% yield) as a white solid. ^{1}H NMR (400 MHz, DMSO-d_6) δ

8.59 (d, J = 2.8 Hz, 1H), 8.54 (d, J = 1.8 Hz, 1H), 8.02 (d, J = 5.1 Hz, 1H), 7.92 (ddd, J = 10.0, 2.8, 1.7 Hz, 1H), 7.35 (d, J = 5.0 Hz, 1H), 3.73 (s, 3H). ^{13}C NMR (101 MHz, DMSO-d_6) δ 161.43, 158.46 (d, J = 253.6 Hz), 145.84, 145.80, 142.59, 142.57, 136.84, 136.73 (d, J = 22.8 Hz), 132.52, 131.53, 127.84, 123.79 (d, J = 19.1 Hz), 52.16. ^{19}F NMR (376 MHz, DMSO-d_6) δ -129.34. HRMS (m/z) (M+H)$^+$: calculated 238.03325, found 238.03302.

Ethyl 5-(quinolin-6-yl)nicotinate (14): Prepared according to general procedure B from 6-bromoquinoline (208 mg, 1.00 mmol, 1.00 equiv) and ethyl 5-(4,4,5,5-tetramethyl-1,3,2-dioxaborolan-2-yl)nicotinate (1.50 equiv). 20 h reaction time. Isolated 220 mg (0.79 mmol, 79% yield) as a white solid. ^{1}H NMR (400 MHz, $CDCl_3$) δ 9.25 (d, J = 2.0 Hz, 1H), 9.14 (d, J = 2.3 Hz, 1H), 8.98 (dd, J = 4.2, 1.7 Hz, 1H), 8.62 (t, J = 2.2 Hz, 1H), 8.30 – 8.22 (m, 2H), 8.08 (d, J = 2.1 Hz, 1H), 8.00 (dd, J = 8.8, 2.1 Hz, 1H), 7.49 (dd, J = 8.3, 4.2 Hz, 1H), 4.48 (q, J = 7.1 Hz, 3H), 1.46 (t, J = 7.1 Hz, 4H). ^{13}C NMR (101 MHz, $CDCl_3$) δ 165.33, 152.01, 151.29, 149.90, 148.15, 136.44, 135.79, 135.61, 134.97, 130.81, 128.59, 128.55, 126.58, 126.35, 122.03, 61.81, 14.44. HRMS (m/z) (M+H)$^+$: calculated 279.11234, found 279.11280.

2-methyl-6-(pyridin-4-yl)quinoline (15)[20]: Prepared according to general procedure B from 6-bromo-2-methylquinoline (222 mg, 1.00 mmol, 1.00 equiv) and 4-(4,4,5,5-tetramethyl-1,3,2-dioxaborolan-2-yl)pyridine (308 mg, 1.50 mmol, 1.00 equiv) using $(PPh_2Me)_2NiCl_2$ (5 mol %), PPh_2Me (10 mol %) and *n*-BuMgCl (11 mol %) as catalyst. Isolated 174 mg (0.79 mmol, 79% yield) as a white solid. ^{1}H NMR (400 MHz, DMSO-d_6) δ 8.72 – 8.66 (m, 2H), 8.41 (d, J = 2.1 Hz, 1H), 8.36 – 8.30 (m, 1H), 8.13 (dd, J = 8.7, 2.1 Hz, 1H), 8.03 (d, J = 8.8 Hz, 1H), 7.88 – 7.80 (m, 2H), 7.48 (d, J = 8.4 Hz, 1H), 2.68 (s, 3H). ^{13}C NMR (151 MHz, DMSO-d_6) δ 159.83, 150.33, 147.45, 146.31, 136.65, 134.06, 129.11, 127.81, 126.31, 126.22, 122.83, 121.40, 24.95. MS (m/z) 221.2 (M+H)$^+$

4-(pyrimidin-5-yl)-1H-indole (16): Prepared according to general procedure A from 5-bromopyrimidine (159 mg, 1.00 mmol, 1.00 equiv) and 4-(4,4,5,5-tetramethyl-1,3,2-dioxaborolan-2-yl)-1H-indole (267 mg, 1.10 mmol, 1.10 equiv). 24 h reaction time. Isolated 149 mg (0.76 mmol, 76% yield) as a white solid. ^{1}H NMR (400 MHz, DMSO-d_6) δ 11.43 (s, 1H), 9.21 (s, 1H), 9.11 (s, 2H), 7.57 – 7.46 (m, 2H), 7.29 – 7.18 (m, 2H), 6.63 – 6.57 (m, 1H). ^{13}C NMR (101 MHz, DMSO-d_6) δ 156.81, 155.70, 136.38, 134.33, 126.84, 125.74, 125.48, 121.51, 119.22, 112.35, 99.43. HRMS (m/z) (M+H)$^+$: calculated 196.08692, found 196.08665.

5-(1H-indol-5-yl)-1-(phenylsulfonyl)-1H-pyrrolo[2,3-b]pyridine (17): Prepared according to general procedure A from 1-benzenesulfonyl-5-bromo-1h-pyrrolo[2,3-b]pyridine (337 mg, 1.00 mmol, 1.00 equiv) and 5-(4,4,5,5-tetramethyl-1,3,2-dioxaborolan-2-yl)-1h-indole (267 mg, 1.10 mmol, 1.10 equiv). 16 h reaction time. Isolated 323 mg (0.86 mmol, 86% yield) as a white solid. ^{1}H NMR (400 MHz, DMSO-d_6) δ 11.19 (s, 1H), 8.66 (d, J = 2.3 Hz, 1H), 8.26 (d, J = 2.2 Hz, 1H), 8.17 – 8.11 (m, 2H), 7.93 (d, J = 4.0 Hz, 1H), 7.84 (d, J = 1.7 Hz, 1H), 7.72 (t, J = 7.4 Hz, 1H), 7.63 (t, J = 7.7 Hz, 2H), 7.50 (d, J = 8.4 Hz, 1H), 7.44 – 7.37 (m, 2H), 6.86 (d, J = 4.1 Hz, 1H), 6.49 (t, J = 2.4 Hz, 1H). ^{13}C NMR (151 MHz, DMSO-d_6) δ 145.60, 143.58, 137.66, 135.57, 134.70, 133.75, 129.63, 128.36, 128.32, 127.87, 127.42, 126.29, 122.69, 120.62, 118.75, 112.03, 106.40, 101.53. One resonance not located. HRMS (m/z) (M+H)$^+$: calculated 374.09577, found 374.09546.

1-(4-(1-benzyl-1H-pyrazol-4-yl)phenyl)propan-1-one (18): Prepared according to general procedure B from 4'-bromopropiophenone (213 mg, 1.00 mmol, 1.00 equiv) and 1-benzyl-4-(4,4,5,5-tetramethyl-1,3,2-dioxaborolan-2-yl)-1h-pyrazole (313 mg, 1.10 mmol, 1.10 equiv). 16 h reaction time. Isolated 278 mg (0.96 mmol, 96% yield) as a white solid. ^{1}H NMR (400 MHz, $CDCl_3$) δ 7.98 – 7.91 (m, 2H), 7.89 (s, 1H), 7.70 (s, 1H), 7.57 – 7.49 (m, 2H), 7.42 – 7.24 (m, 5H), 5.35 (s, 2H), 2.99 (q, *J* = 7.2 Hz, 2H), 1.23 (t, *J* = 7.2 Hz, 3H). ^{13}C NMR (101 MHz, $CDCl_3$) δ 200.20, 137.42, 137.23, 136.18, 134.87, 129.07, 128.89, 128.42, 127.93, 126.91, 125.31, 122.59, 56.50, 31.76, 8.44. HRMS (m/z) $(M+H)^{+}$: calculated 291.14919, found 291.14899.

3-(1-benzyl-1H-pyrazol-4-yl)pyridin-2-amine (19): Prepared according to general procedure A from 2-amino-3-bromopyridine (173 mg, 1.00 mmol, 1.00 equiv) and 1-benzyl-4-(4,4,5,5-tetramethyl-1,3,2-dioxaborolan-2-yl)-1h-pyrazole (426 mg, 1.50 mmol, 1.50 equiv). 16 h reaction time. Isolated 219 mg (0.87 mmol, 87% yield) as a white solid. ^{1}H NMR (400 MHz, DMSO-d_6) δ 8.18 (d, *J* = 0.9 Hz, 1H), 7.88 (dd, *J* = 4.9, 1.8 Hz, 1H), 7.78 (d, *J* = 0.9 Hz, 1H), 7.49 (dd, *J* = 7.4, 1.8 Hz, 1H), 7.39 – 7.26 (m, 5H), 6.61 (dd, *J* = 7.4, 4.9 Hz, 1H), 5.62 (s, 2H), 5.35 (s, 2H). ^{13}C NMR (176 MHz, DMSO-d_6) δ 156.16, 145.87, 137.90, 137.39, 135.80, 128.53, 128.46, 127.71, 127.60, 117.95, 113.14, 112.20, 54.95. HRMS (m/z) $(M+H)^{+}$: calculated 251.12912, found 251.12857.

Methyl 2-(1-benzyl-1H-pyrazol-4-yl)isonicotinate (20): Prepared according to general procedure A from methyl 2-chloroisonicotinate (172 mg, 1.00 mmol, 1.00 equiv) and 1-benzyl-4-(4,4,5,5-tetramethyl-1,3,2-dioxaborolan-2-yl)-1h-pyrazole (313 mg, 1.10 mmol, 1.10 equiv). 16 h reaction time. Isolated 235 mg (0.80 mmol, 80% yield) as a white solid. ^{1}H NMR (400 MHz, $CDCl_3$) δ 8.69 (dt, *J* = 5.0, 0.9 Hz, 1H), 8.11 (s, 1H), 8.00 (dd, *J* = 2.8, 1.3 Hz, 2H), 7.64 (dt, *J* = 5.2, 1.1 Hz, 1H), 7.47 – 7.23 (m, 5H), 5.38 (s, 2H), 3.99 (d, *J* = 0.9 Hz, 3H). ^{13}C NMR (176 MHz, $CDCl_3$) δ 165.86, 153.14, 150.49, 138.05, 136.01, 129.07, 128.48, 128.47, 128.45, 128.44, 128.39, 128.10, 123.51, 120.19, 120.16, 120.13, 118.87, 118.84, 118.83, 56.61, 52.82. One resonance not located. HRMS (m/z) $(M+H)^{+}$: calculated 294.12370, found 294.12326.

6-(1-benzyl-1H-pyrazol-4-yl)-7-methylquinoxaline (21): A vial was charged with 6-chloro-7-methylquinoxaline (179 mg, 1.00 mmol, 1.00 equiv), 1-benzyl-4-(4,4,5,5-tetramethyl-1,3,2-dioxaborolan-2-yl)-1h-pyrazole (313 mg, 1.10 mmol, 1.10 equiv), K_3PO_4 (531 mg, 2.50 mmol, 2.50 equiv), PPh_2Me (19.8 mg, 0.099 mmol, 9.9 mol %), (TMEDA)Ni(o-tolyl)Cl (9.0 mg, 0.030 equiv, 3.0 mol %), 2-MeTHF (2.5 mL) and water (0.5 mL). The reaction mixture was stirred for 16 h at 70 °C, then worked up according to general procedure A. Isolated 242 mg (0.81 mmol, 81% yield) as an orange oil. ^{1}H NMR (400 MHz, DMSO-d_6) δ 8.88 – 8.82 (m, 2H), 8.38 (d, *J* = 0.9 Hz, 1H), 8.08 (s, 1H), 7.97 (dd, *J* = 6.4, 1.0 Hz, 2H), 7.42 – 7.26 (m, 5H), 5.42 (s, 2H), 2.63 (d, *J* = 0.9 Hz, 3H). ^{13}C NMR (176 MHz, DMSO-d_6) δ 145.25, 145.10, 141.15, 141.01, 139.08, 138.49, 137.41, 135.42, 130.13, 130.11, 129.51, 128.55, 127.65, 127.64, 127.60, 127.58, 126.90, 126.90, 119.87, 54.97, 21.76. HRMS (m/z) $(M+H)^{+}$: calculated 301.14477, found 301.14432.

4-(1-benzyl-1H-pyrazol-4-yl)-7-(phenylsulfonyl)-7H-pyrrolo[2,3-d]pyrimidine (22): Prepared according to general procedure A from 4-chloro-7-(phenylsulfonyl)-7h-pyrrolo[2,3-d]pyrimidine (294 mg, 1.00 mmol, 1.00 equiv) and 1-benzyl-4-(4,4,5,5-tetramethyl-1,3,2-dioxaborolan-2-yl)-1h-pyrazole (313 mg, 1.10 mmol, 1.10 equiv). 16 h reaction time. Isolated 381 mg (0.92 mmol, 92% yield) as a tan solid. ^{1}H NMR (400 MHz, DMSO-d_6) δ 8.87 (d, *J* = 0.8

Hz, 1H), 8.83 (s, 1H), 8.32 (d, $J = 0.7$ Hz, 1H), 8.20 – 8.12 (m, 2H), 8.06 (d, $J = 4.1$ Hz, 1H), 7.80 – 7.71 (m, 1H), 7.70 – 7.61 (m, 2H), 7.39 – 7.24 (m, 6H), 5.43 (s, 2H). ^{13}C NMR (151 MHz, DMSO-d_6) δ 152.90, 151.94, 151.32, 139.46, 137.07, 136.90, 135.15, 132.04, 129.78, 128.57, 127.80, 127.72, 127.66, 126.99, 119.78, 114.78, 104.77, 55.16. HRMS (m/z) (M+H)$^+$: calculated 416.11757, found 416.11703.

4-(1-(1-ethoxyethyl)-1H-pyrazol-4-yl)-7-((2-(trimethylsilyl)ethoxy)methyl)-7H-pyrrolo[2,3-d]pyrimidine (23)[21]: Prepared according to general procedure A from 1-(1-ethoxyethyl)-4-(4,4,5,5-tetramethyl-1,3,2-dioxaborolan-2-yl)-1h-pyrazole (293 mg, 1.10 mmol, 1.10 equiv) and 4-chloro-7-((2-(trimethylsilyl)ethoxy)methyl)-7h-pyrrolo[2,3-d]pyrimidine (284 mg, 1.00 equiv, 1.00 mmol). Reaction time 20 h. Isolated 355 mg (0.92 mmol, 92% yield) as a viscous light-yellow oil. ^{1}H NMR (400 MHz, DMSO-d_6) δ 8.82 (s, 1H), 8.76 (d, $J = 0.9$ Hz, 1H), 8.37 (s, 1H), 7.77 (d, $J = 3.7$ Hz, 1H), 7.14 (d, $J = 3.7$ Hz, 1H), 5.67 (q, $J = 5.9$ Hz, 1H), 5.63 (s, 2H), 3.53 (d, $J = 7.9$ Hz, 1H), 3.51 – 3.45 (m, 1H), 3.31 – 3.20 (m, 1H), 1.69 (d, $J = 6.0$ Hz, 3H), 1.06 (t, $J = 7.0$ Hz, 4H), 0.82 (t, $J = 8.0$ Hz, 2H), -0.11 (d, $J = 0.9$ Hz, 9H). ^{13}C NMR (101 MHz, DMSO-d_6) δ 151.62, 151.28, 150.38, 138.97, 129.95, 129.45, 120.87, 113.20, 100.50, 86.63, 72.28, 65.60, 63.20, 21.06, 17.10, 14.74, -1.41. MS (m/z) 388.1 (M+H)$^+$

4-(1-benzyl-1H-pyrazol-4-yl)thiazole (24): Prepared according to general procedure A from 4-bromothiazole (164 mg, 1.00 mmol, 1.00 equiv) and 1-benzyl-4-(4,4,5,5-tetramethyl-1,3,2-dioxaborolan-2-yl)-1h-pyrazole (284 mg, 1.00 mmol, 1.00 equiv). Isolated 171 mg (0.71 mmol, 71% yield) as a white solid. ^{1}H NMR (700 MHz, DMSO-d_6) δ 9.10 (d, $J = 2.0$ Hz, 1H), 8.25 (d, $J = 0.9$ Hz, 1H), 7.92 (d, $J = 0.8$ Hz, 1H), 7.73 (d, $J = 2.0$ Hz, 1H), 7.37 – 7.32 (m, 2H), 7.32 – 7.25 (m, 3H), 5.37 (s, 2H). ^{13}C NMR (176 MHz, DMSO-d_6) δ 154.06, 148.72, 137.39, 137.16, 128.52, 128.18, 127.64, 127.58, 117.77, 111.10, 54.89. HRMS (m/z) (M+H)$^+$: calculated 416.11757, found 416.11703.

1'-benzyl-1-methyl-1H,1'H-3,4'-bipyrazole (25): Prepared according to general procedure A from 3-bromo-1-methyl-1H-pyrazole (161 mg, 1.00 mmol, 1.00 equiv) and 1-benzyl-4-(4,4,5,5-tetramethyl-1,3,2-dioxaborolan-2-yl)-1h-pyrazole (284 mg, 1.00 mmol, 1.00 equiv). Reaction time 17 h. Isolated 178 mg (0.75 mmol, 75% yield) as a colorless oil. ^{1}H NMR (400 MHz, $CDCl_3$) δ 7.85 (d, $J = 0.8$ Hz, 1H), 7.73 (d, $J = 0.8$ Hz, 1H), 7.41 – 7.28 (m, 6H), 6.32 (d, $J = 2.3$ Hz, 1H), 5.35 (s, 2H), 3.92 (s, 3H). ^{13}C NMR (101 MHz, $CDCl_3$) δ 144.94, 137.47, 136.42, 131.19, 128.93, 128.21, 127.96, 126.55, 116.74, 102.91, 56.30, 38.90. HRMS (m/z) (M+H)$^+$: calculated 239.12912, found 239.12878.

Tert-butyl 4-(quinolin-5-yl)-5,6-dihydropyridine-1(2H)-carboxylate (26): Prepared according to general procedure A from 6-bromoquinoline (208 mg, 1.00 mmol, 1.00 equiv) and 1-benzyl-4-(4,4,5,5-tetramethyl-1,3,2-dioxaborolan-2-yl)-1h-pyrazole (340 mg, 1.10 mmol, 1.10 equiv). 16 h reaction time. Isolated 294 mg (0.95 mmol, 95% yield) as white solid. ^{1}H NMR (400 MHz, $CDCl_3$) δ 8.89 (dd, $J = 4.2, 1.7$ Hz, 1H), 8.29 (ddd, $J = 8.5, 1.7, 0.9$ Hz, 1H), 8.02 (dt, $J = 8.5, 1.1$ Hz, 1H), 7.65 (dd, $J = 8.5, 7.1$ Hz, 1H), 7.41 – 7.30 (m, 2H), 5.75 (s, 1H), 4.13 (q, $J = 2.9$ Hz, 2H), 3.71 (t, $J = 5.6$ Hz, 2H), 2.49 (app s, 2H), 1.51 (s, 9H). ^{13}C NMR (101 MHz, $CDCl_3$) δ 155.08, 150.33, 148.67, 141.26, 135.21, 133.92, 129.06, 128.83, 126.29, 125.41, 125.05, 120.98, 79.97, *43.62*, *40.02*, 31.14, 28.62. Peaks in italics were located by HSQC. HRMS (m/z) (M+H)$^+$: calculated 311.17540, found 311.17490.

1-(4'-methyl-[1,1'-biphenyl]-4-yl)ethenone (27)[22]: Prepared according to general procedure A from 4'-bromoacetophenone (199 mg, 1.00 mmol, 1.00 equiv) and 4,4,5,5-tetramethyl-2-(p-tolyl)-1,3,2-dioxaborolane (240 mg, 1.10 mmol, 1.10 equiv). 16 h reaction time. Isolated 188 mg (0.89 mmol, 89% yield) as white solid. ^{1}H NMR (400 MHz, DMSO-d_6) δ 8.06 – 7.98 (m, 2H), 7.83 – 7.76 (m, 2H), 7.68 – 7.61 (m, 2H), 7.35 – 7.27 (m, 2H), 2.60 (s, 3H), 2.36 (s, 3H). ^{13}C NMR (101 MHz, DMSO-d_6) δ 197.42, 144.44, 137.90, 135.98, 135.37, 129.69, 129.68, 128.88, 126.82, 126.80, 126.78, 126.51, 26.72, 20.71. MS (m/z) 211.4 $(M+H)^+$

4-(2-methoxypyrimidin-5-yl)benzonitrile (28)[23]: Prepared according to general procedure C from 4-bromobenzonitrile (1.00 g, 5.44 mmol, 1.00 equiv) and potassium 2-methoxypyrimidine-5-trifluoroborate (1.463 g, 5.98 mmol, 1.10 equiv). 16 h reaction time. Isolated 840 mg (3.98 mmol, 73% yield) as white solid. ^{1}H NMR (400 MHz, $CDCl_3$) δ 8.74 (s, 2H), 7.80 – 7.74 (m, 2H), 7.67 – 7.60 (m, 2H), 4.07 (s, 3H). ^{13}C NMR (101 MHz, $CDCl_3$) δ 165.81, 157.61, 139.11, 133.18, 127.14, 126.59, 118.50, 112.12, 55.42. MS (m/z) 212.2 $(M+H)^+$.

6-(pyridin-3-yl)quinoline (29): Prepared according to general procedure C from 6-bromoquinoline (208 mg, 1.00 mmol, 1.00 equiv) and potassium pyridine-3-trifluoroborate (277 mg, 1.50 mmol, 1.50 equiv) at 90 °C. 22 h reaction time. Isolated 184 mg (0.89 mmol, 89% yield) as white solid. Approximately 15% of an impurity was present by NMR. ^{1}H NMR (400 MHz, DMSO-d_6) δ 9.07 (dd, J = 2.4, 0.9 Hz, 1H), 8.95 (ddd, J = 5.9, 3.6, 1.9 Hz, 1H), 8.64 (dd, J = 4.7, 1.5 Hz, 1H), 8.45 (dd, J = 8.3, 1.7 Hz, 1H), 8.40 (d, J = 1.9 Hz, 1H), 8.29 – 8.22 (m, 1H), 8.20 – 8.10 (m, 2H), 7.64 – 7.53 (m, 2H). ^{13}C NMR (176 MHz, DMSO-d_6) δ 150.98, 148.84, 148.00, 147.29, 136.36, 134.96, 134.79, 134.47, 129.75, 128.42, 128.12, 126.05, 123.95, 122.00. HRMS (m/z) $(M+H)^+$: calculated 207.09167, found 207.09134.

6-vinylquinoline (30)[24]: Prepared according to general procedure C from 6-bromoquinoline (208 mg, 1.00 mmol, 1.00 equiv) and potassium vinyltrifluoroborate (201 mg, 1.50 mmol, 1.50 equiv). 20 h reaction time. Isolated 112 mg (0.72 mmol, 72% yield) as a yellow oil. ^{1}H NMR (400 MHz, $CDCl_3$) δ 8.89 – 8.82 (m, 1H), 8.11 (dt, J = 8.2, 1.4 Hz, 1H), 8.05 (d, J = 8.8 Hz, 1H), 7.86 (dd, J = 8.8, 1.8 Hz, 1H), 7.71 (s, 1H), 7.37 (ddd, J = 8.3, 4.3, 1.1 Hz, 1H), 6.89 (ddd, J = 17.6, 10.9, 1.3 Hz, 1H), 5.90 (dd, J = 17.5, 1.0 Hz, 1H), 5.39 (dd, J = 10.8, 0.9 Hz, 1H). ^{13}C NMR (101 MHz, $CDCl_3$) δ 150.35, 148.32, 136.30, 136.18, 135.86, 129.78, 128.52, 127.05, 125.93, 121.56, 115.54.

References

1. Standley, E. A.; Smith, S. J.; Müller, P.; Jamison, T. F., A Broadly Applicable Strategy for Entry into Homogeneous Nickel(0) Catalysts from Air-Stable Nickel(II) Complexes. *Organometallics* **2014,** *33* (8), 2012-2018.
2. Tolman, C. A.; Seidel, W. C.; Gosser, L. W., Formation of three-coordinate nickel(0) complexes by phosphorus ligand dissociation from NiL4. *J. Am. Chem. Soc.* **1974,** *96* (1), 53-60.
3. Fisher, K. J.; Alyea, E. C., Metal vapour synthesis of zero-valent nickel phosphine complexes and their characterization by 31P NMR spectroscopy. *Polyhedron* **1989,** *8* (1), 13-15.
4. Nakamura, Y.; Maruya, K.-I.; Mizoroki, T., 31P NMR study on phosphorus ligand exchange on bis (triphenylphosphine)-o-tolylnickel (II) bromide. *J. Organomet. Chem.* **1976,** *104* (1), C5-C8.
5. 中村，義.; 丸谷，建.; 溝呂木，勉., ³¹P-NMRによる<I>trans</I>-ハロゲノ(o-トリル)ビス(トリフェニルホスフィン)金属(II)とリン配位子との置換反応. *日本化学会誌(化学と工業化学)* **1978,** *1978* (11), 1486-1491.
6. Smeets, A.; Van den Bergh, K.; De Winter, J.; Gerbaux, P.; Verbiest, T.; Koeckelberghs, G., Incorporation of Different End Groups in Conjugated Polymers Using Functional Nickel Initiators. *Macromolecules* **2009,** *42* (20), 7638-7641.
7. Netherton, M. R.; Fu, G. C., Air-stable trialkylphosphonium salts: Simple, practical, and versatile replacements for air-sensitive trialkylphosphines. Applications in stoichiometric and catalytic processes. *Org. Lett.* **2001,** *3* (26), 4295-4298.
8. Li, T.; Lough, A. J.; Morris, R. H., An Acidity Scale of Tetrafluoroborate Salts of Phosphonium and Iron Hydride Compounds in [D2]Dichloromethane. *Chemistry – A European Journal* **2007,** *13* (13), 3796-3803.
9. Zoltán, N.; Réka, A.; János T., C.; Ferenc, B.; Regina, G.; Bálint, V.; Bálint, N.; Zoltán, M.; János, D.; Zsombor, G.; Gergely L., T., *Curse or Blessing? Influence of Impurities on Cross-Coupling—Guideline for Elucidating Catalysts*. 2021.
10. Arvela, R. K.; Leadbeater, N. E.; Sangi, M. S.; Williams, V. A.; Granados, P.; Singer, R. D., A Reassessment of the Transition-Metal Free Suzuki-Type Coupling Methodology. *The Journal of Organic Chemistry* **2005,** *70* (1), 161-168.
11. Stankevič, M.; Pisklak, J.; Włodarczyk, K., Aryl group – a leaving group in arylphosphine oxides. *Tetrahedron* **2016,** *72* (6), 810-824.
12. LaBerge, N.; Love, J., Nickel-Catalyzed Decarbonylative Cou-pling of Aryl Esters and Arylboronic Acids. *Eur. J. Org. Chem.* **2015,** *2015* (25), 5546-5553.
13. Sase, S.; Jaric, M.; Metzger, A.; Malakhov, V.; Knochel, P., One-Pot Negishi Cross-Coupling Reactions of In Situ Generated Zinc Reagents with Aryl Chlorides, Bromides, and Triflates. *The Journal of Organic Chemistry* **2008,** *73* (18), 7380-7382.
14. Wilson, K.; Murray, J.; Jamieson, C.; Watson, A., Cyrene as a Bio-Based Solvent for the Suzuki–Miyaura Cross-Coupling. *Synlett* **2017,** *29* (05), 650-654.
15. Zhou, X.-X.; Shao, L.-X., N-Heterocyclic Carbene/Pd(II)/1-Methylimidazole Complex Catalyzed Suzuki-Miyaura Coupling Reaction of Aryl Chlorides in Water. *Synthesis* **2011,** *2011* (19), 3138-3142.
16. Points, G.; Stout, K.; Beaudry, C., Regioselective Formation of Substituted Indoles: Formal Synthesis of Lysergic Acid. *Chemistry - A European Journal* **2020,** *26* (70), 16655-16658.
17. Kuriyama, M.; Matsuo, S.; Shinozawa, M.; Onomura, O., Ether-Imidazolium Carbenes for Suzuki–Miyaura Cross-Coupling of Heteroaryl Chlorides with Aryl/Heteroarylboron Reagents. *Org. Lett.* **2013,** *15* (11), 2716-2719.
18. Wang, L.; Cui, X.; Li, J.; Wu, Y.; Zhu, Z.; Wu, Y., Synthesis of Biaryls through a One-Pot Tandem Borylation/Suzuki–Miyaura Cross-Coupling Reaction Catalyzed by a Palladacycle. *Eur. J. Org. Chem.* **2012,** *2012* (3), 595-603.
19. Bratt, E.; Verho, O.; Johansson, M.; Bäckvall, J.-E., A General Suzuki Cross-Coupling Reaction of Heteroaromatics Catalyzed by Nanopalladium on Amino-Functionalized Siliceous Mesocellular Foam. *The Journal of Organic Chemistry* **2014,** *79* (9), 3946-3954.
20. Oberli, M.; Buchwald, S., A General Method for Suzuki–Miyaura Coupling Reactions Using Lithium Triisopropyl Borates. *Org. Lett.* **2012,** *14* (17), 4606-4609.

21. Lin, Q.; Meloni, D.; Pan, Y.; Xia, M.; Rodgers, J.; Shepard, S.; Li, M.; Galya, L.; Metcalf, B.; Yue, T.-Y.; Liu, P.; Zhou, J., Enantioselective Synthesis of Janus Kinase Inhibitor INCB018424 via an Organocatalytic Aza-Michael Reaction. *Org. Lett.* **2009,** *11* (9), 1999-2002.
22. Jiang, Z.-J.; Li, Z.-H.; Yu, J.-B.; Su, W.-K., Liquid-Assisted Grinding Accelerating: Suzuki–Miyaura Reaction of Aryl Chlorides under High-Speed Ball-Milling Conditions. *The Journal of Organic Chemistry* **2016,** *81* (20), 10049-10055.
23. Molander, G. A.; Canturk, B.; Kennedy, L. E., Scope of the Suzuki−Miyaura Cross-Coupling Reactions of Potassium Heteroaryltrifluoroborates. *The Journal of Organic Chemistry* **2009,** *74* (3), 973-980.
24. Cong, F.; Wei, Y.; Tang, P., Combining photoredox and silver catalysis for azidotrifluoromethoxylation of styrenes. *Chem. Commun.* **2018,** *54* (35), 4473-4476.

NMR Spectra

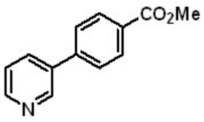

3

1H NMR (400 MHz, CDCl3)

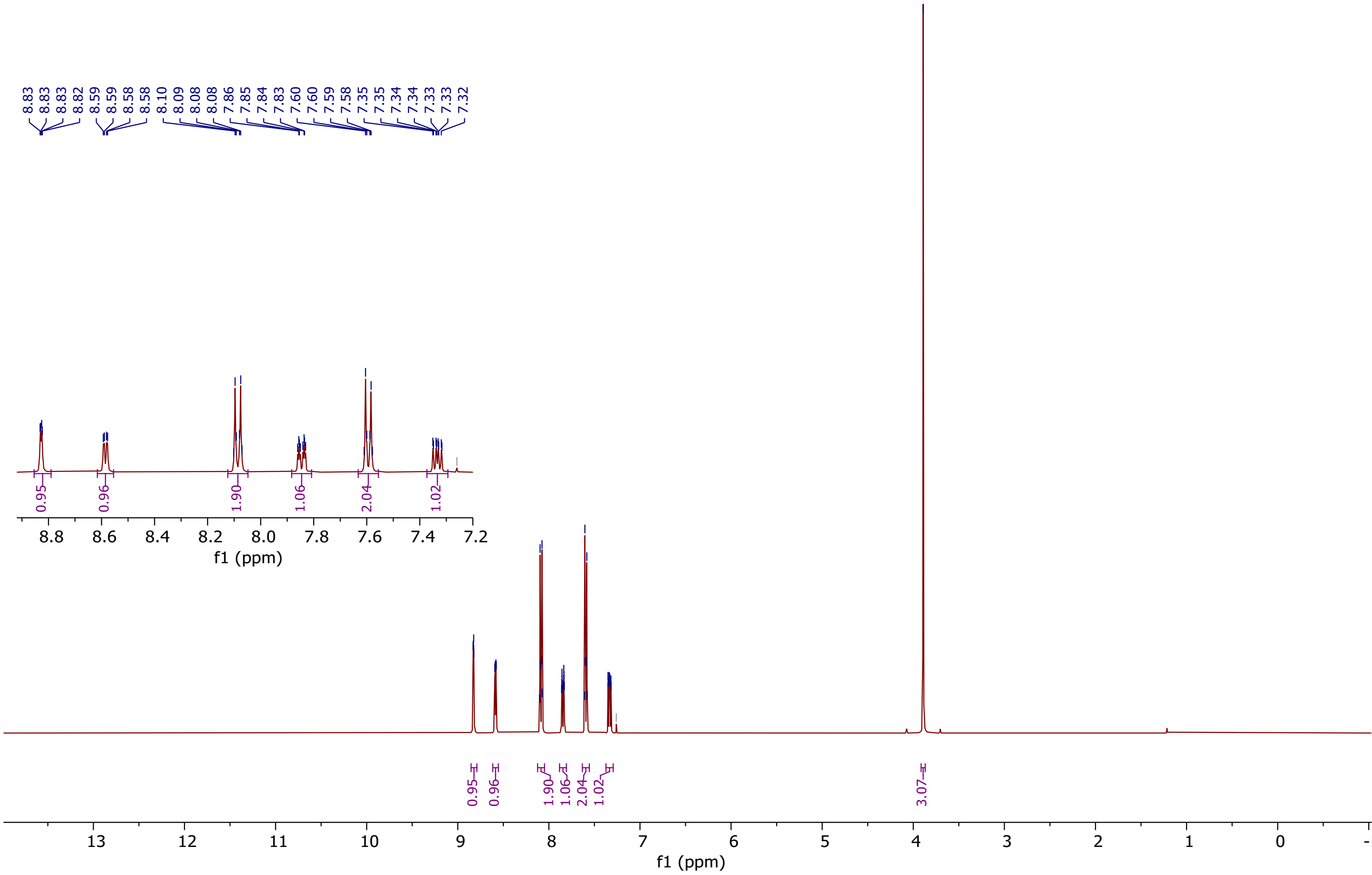

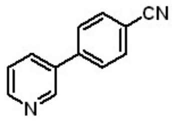

4

1H NMR (400 MHz, CDCl3)

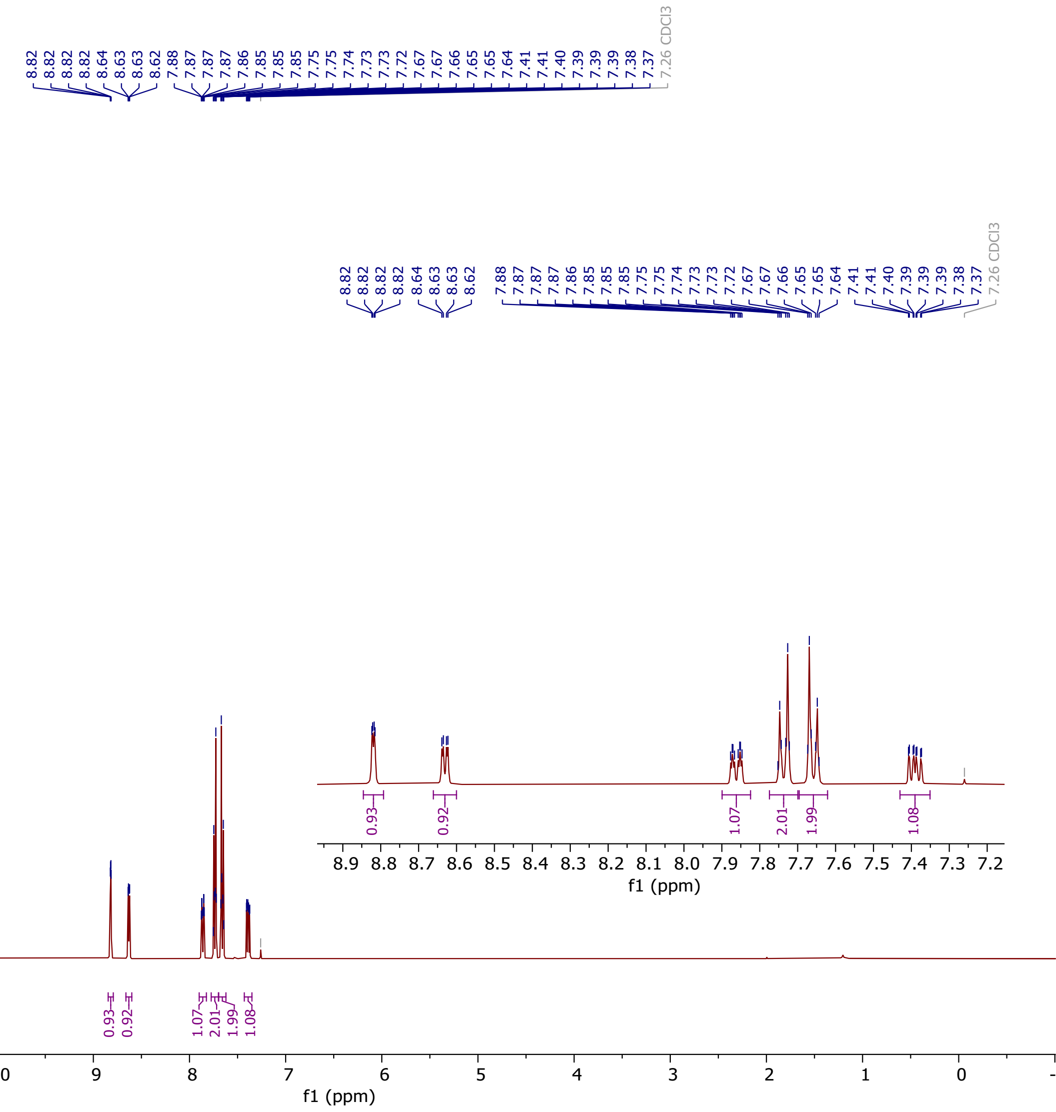

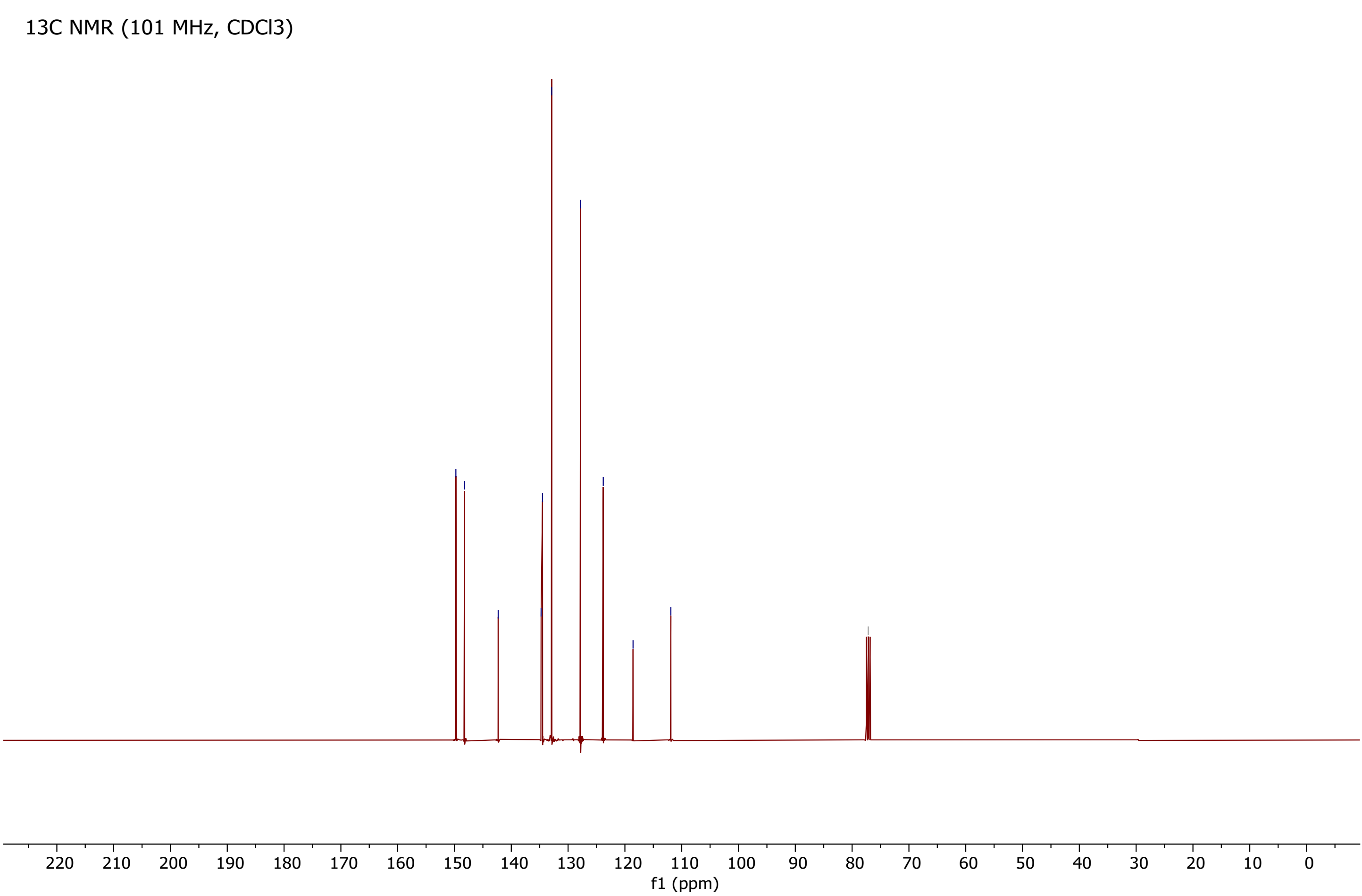

13C NMR (101 MHz, CDCl3)
149.75
148.23
142.31
134.75
134.50
132.87
127.80
123.81
118.57
111.91
77.16 CDCl3
220 210 200 190 180 170 160 150 140 130 120 110 100 90 80 70 60 50 40 30 20 10 0
f1 (ppm)

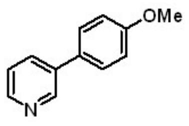

5

1H NMR (400 MHz, CDCl3)

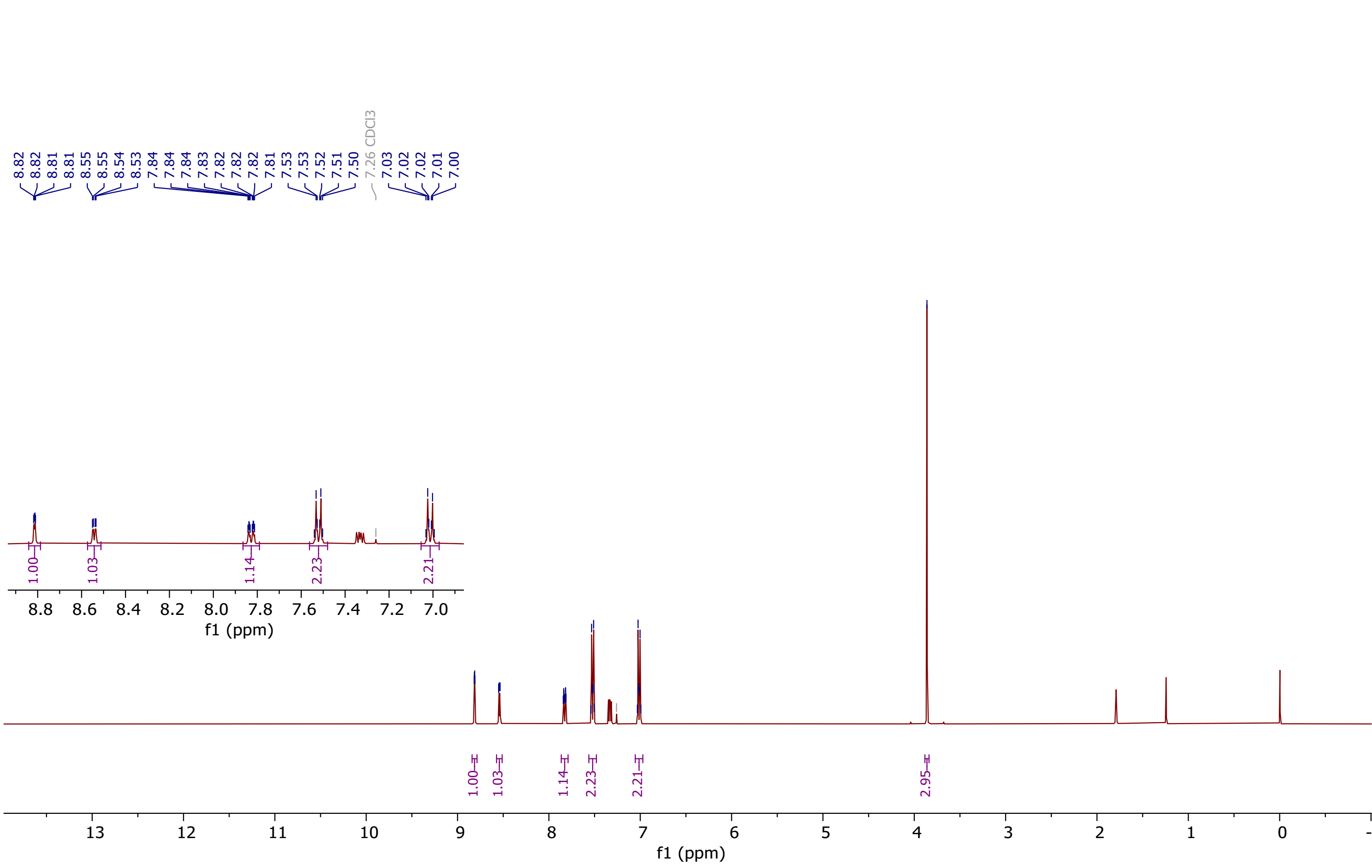

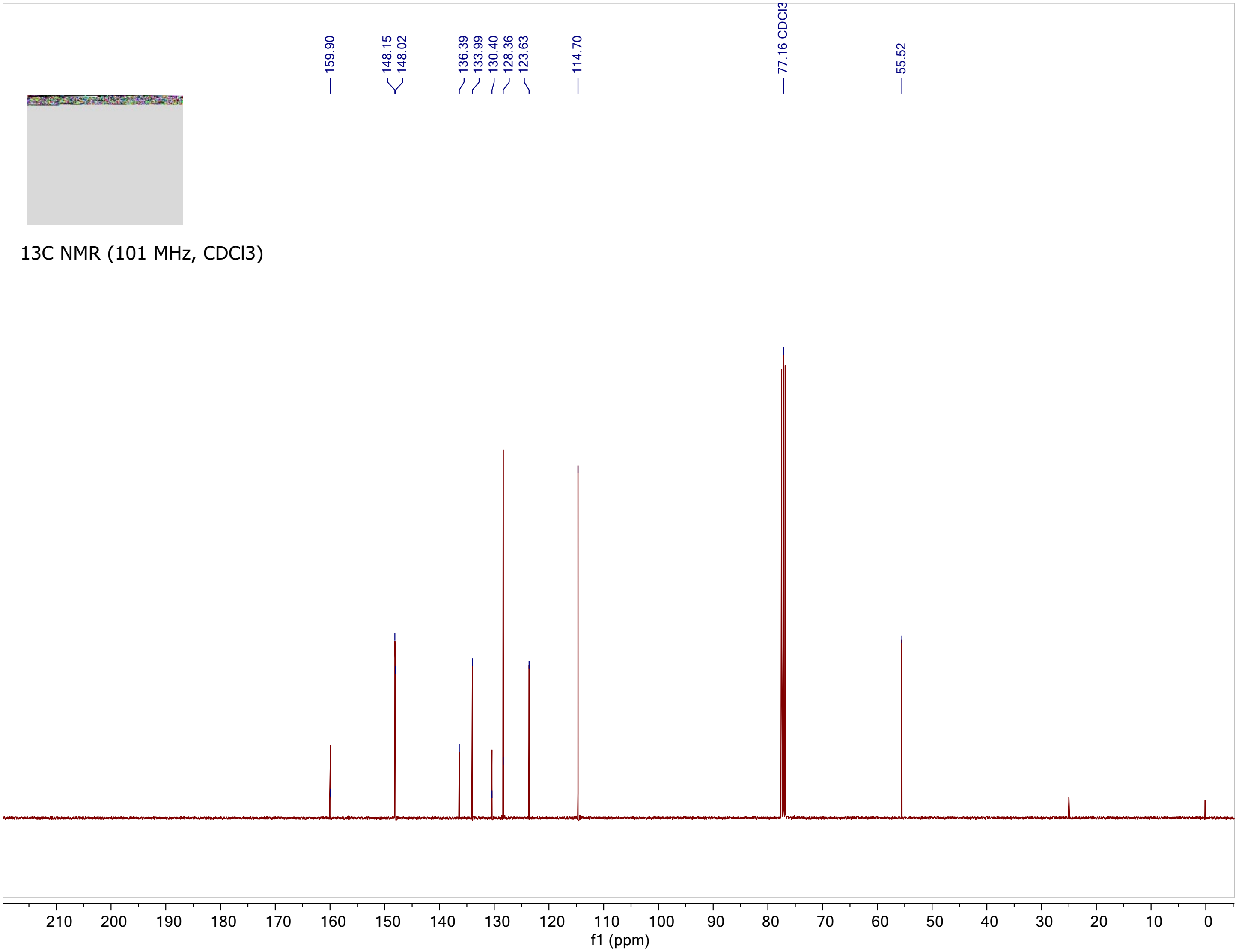
159.90
148.15
148.02
136.39
133.99
130.40
128.36
123.63
114.70
77.16 CDCl3
55.52
13C NMR (101 MHz, CDCl3)
210
200
190
180
170
160
150
140
130
120
110
100
90
80
70
60
50
40
30
20
10
0
f1 (ppm)

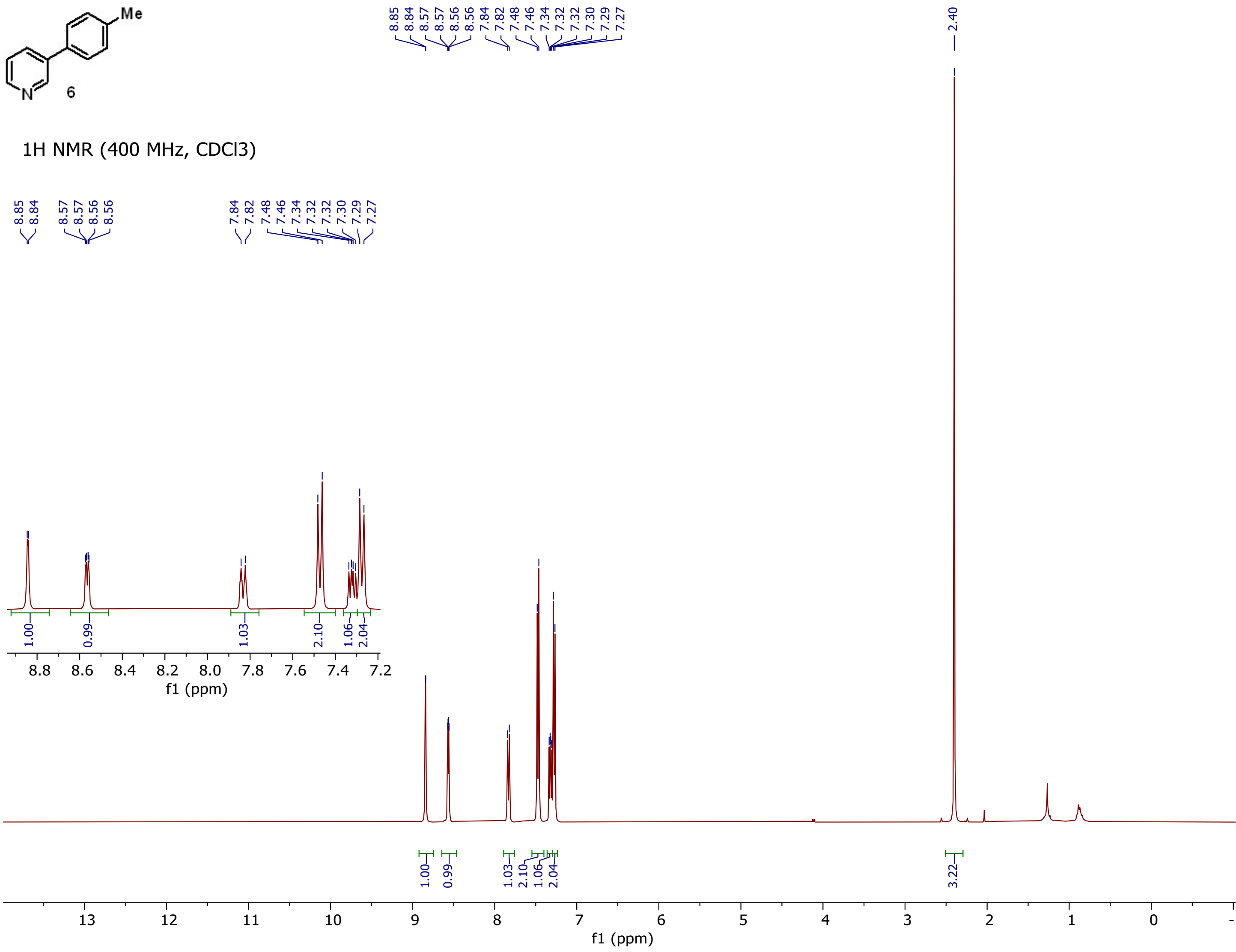
Me
6
1H NMR (400 MHz, CDCl3)
8.85
8.84
8.57
8.57
8.56
8.56
7.84
7.82
7.48
7.46
7.34
7.32
7.32
7.30
7.29
7.27
2.40
1.00
0.99
1.03
2.10
1.06
2.04
3.22
f1 (ppm)

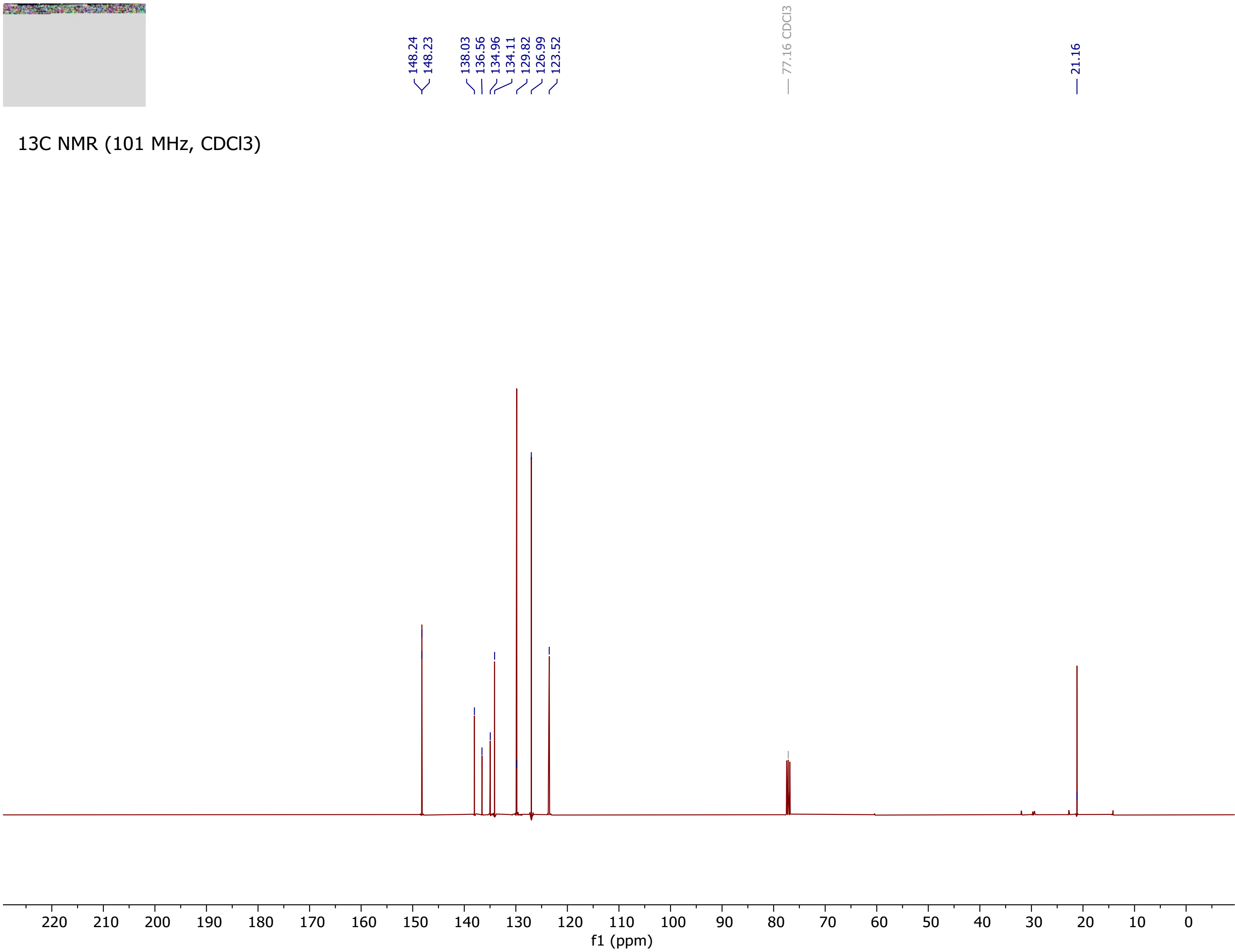
13C NMR (101 MHz, CDCl3)
148.24
148.23
138.03
136.56
134.96
134.11
129.82
126.99
123.52
77.16 CDCl3
21.16
220 210 200 190 180 170 160 150 140 130 120 110 100 90 80 70 60 50 40 30 20 10 0
f1 (ppm)

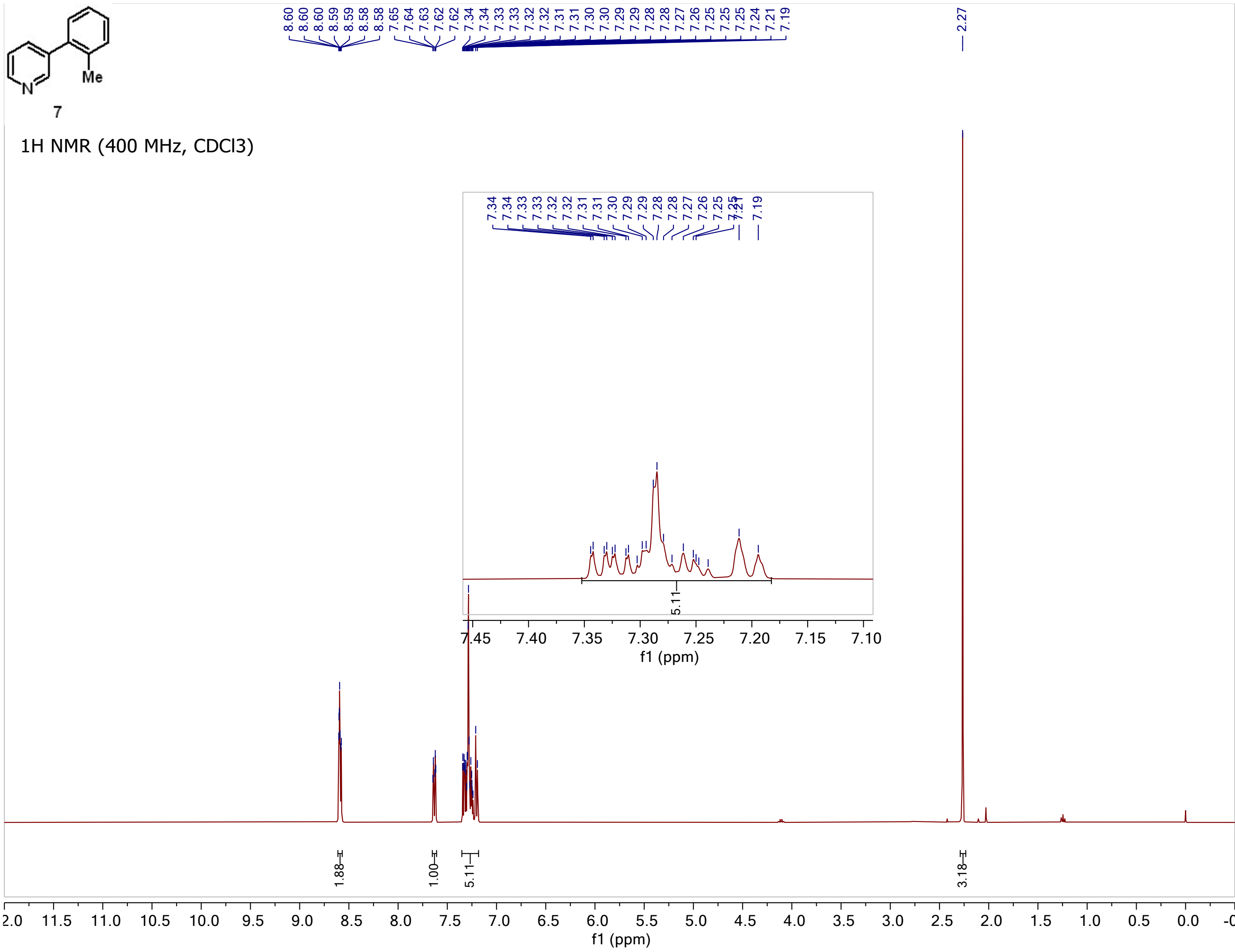

Me
7
1H NMR (400 MHz, CDCl3)
8.60
8.60
8.60
8.59
8.59
8.58
8.58
7.65
7.64
7.63
7.62
7.62
7.34
7.34
7.33
7.33
7.32
7.32
7.31
7.31
7.30
7.29
7.29
7.28
7.28
7.27
7.26
7.25
7.25
7.24
7.21
7.19
2.27
1.88
1.00
5.11
3.18
f1 (ppm)

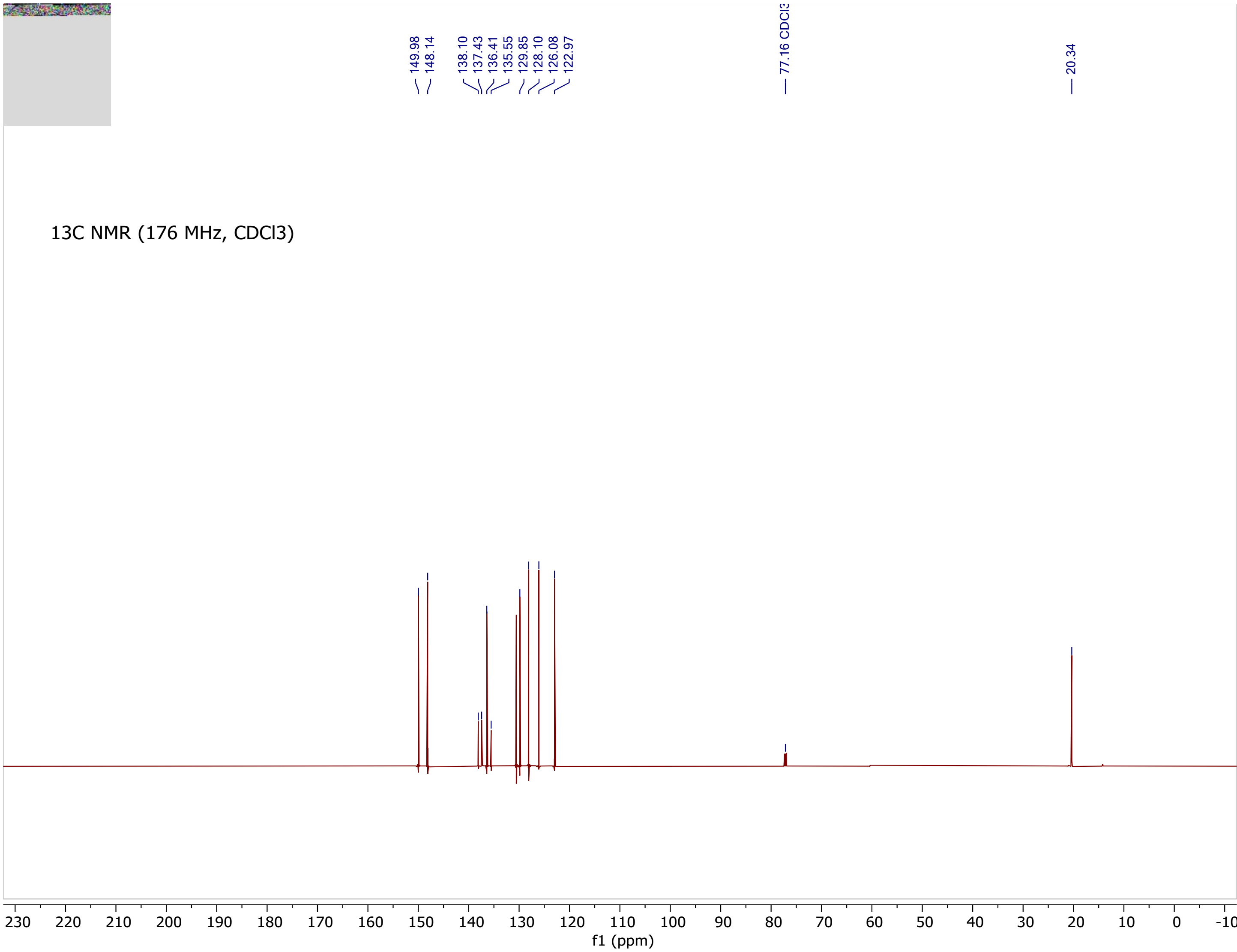

149.98
148.14
138.10
137.43
136.41
135.55
129.85
128.10
126.08
122.97
77.16 CDCl3
20.34
13C NMR (176 MHz, CDCl3)
230 220 210 200 190 180 170 160 150 140 130 120 110 100 90 80 70 60 50 40 30 20 10 0 -10
f1 (ppm)

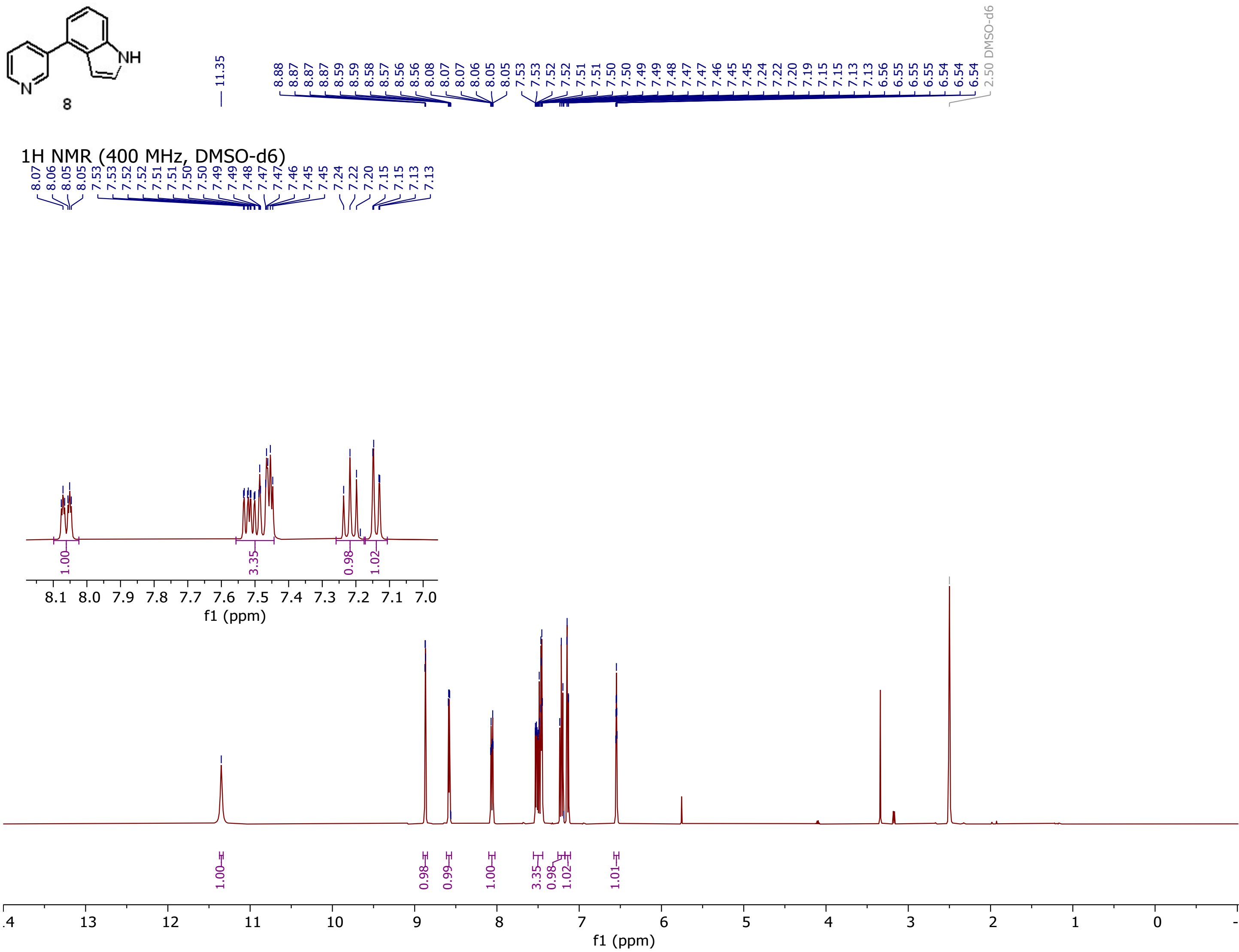

NH
N
8
11.35
8.88 8.87 8.87 8.87 8.59 8.59 8.58 8.57 8.56 8.56 8.08 8.07 8.06 8.05 8.05 7.53 7.53 7.52 7.52 7.51 7.51 7.50 7.50 7.49 7.49 7.48 7.47 7.47 7.46 7.45 7.45 7.24 7.22 7.20 7.19 7.15 7.15 7.13 7.13 6.56 6.55 6.55 6.54 6.54 6.54
2.50 DMSO-d6
1H NMR (400 MHz, DMSO-d6)
8.07 8.06 8.05 8.05 7.53 7.53 7.52 7.52 7.51 7.51 7.50 7.50 7.49 7.49 7.48 7.47 7.47 7.46 7.45 7.45 7.24 7.22 7.20 7.15 7.15 7.13 7.13
1.00 3.35 0.98 1.02
8.1 8.0 7.9 7.8 7.7 7.6 7.5 7.4 7.3 7.2 7.1 7.0
f1 (ppm)
1.00 0.98 0.99 1.00 3.35 0.98 1.02 1.01
.4 13 12 11 10 9 8 7 6 5 4 3 2 1 0 -
f1 (ppm)

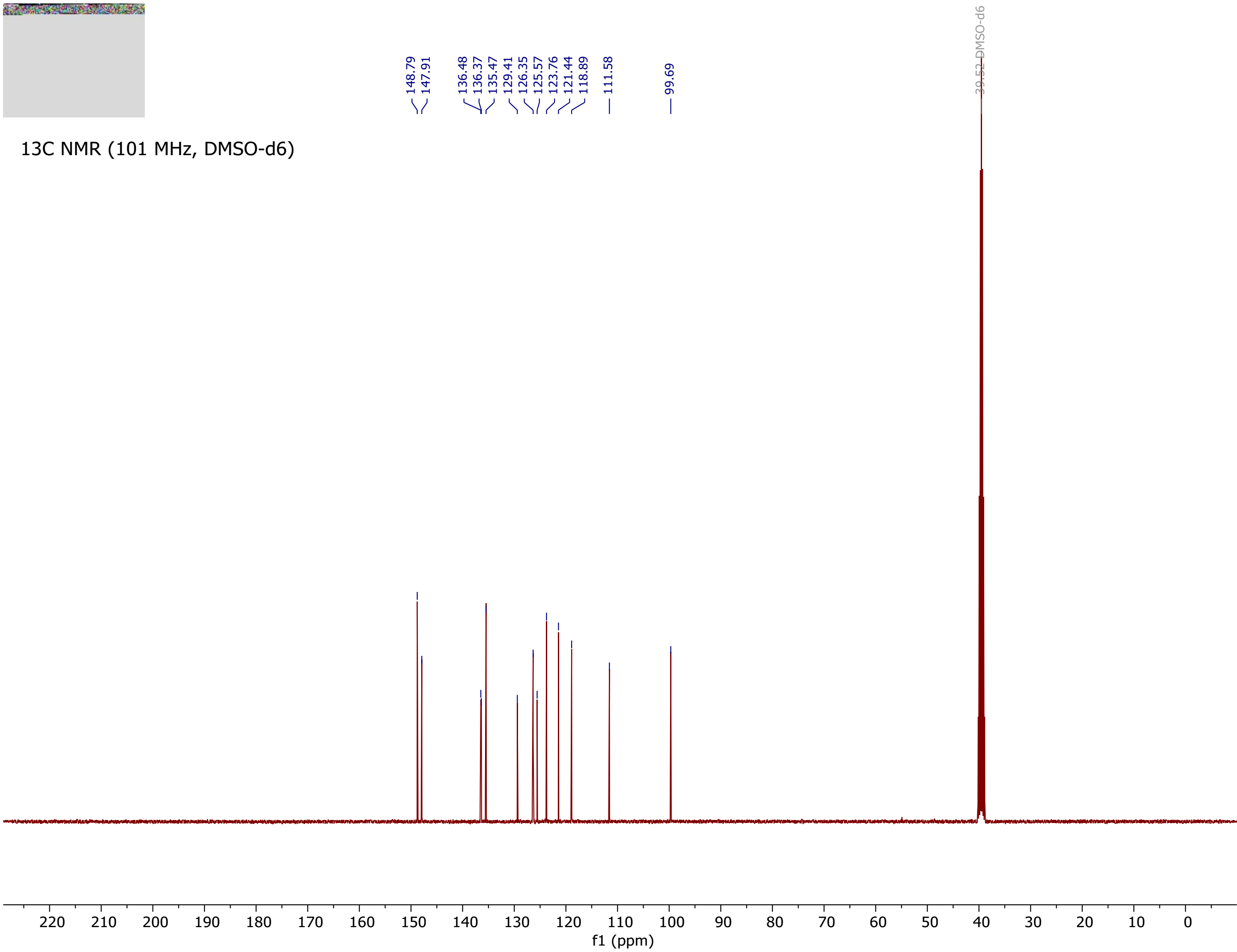
13C NMR (101 MHz, DMSO-d6)
148.79
147.91
136.48
136.37
135.47
129.41
126.35
125.57
123.76
121.44
118.89
111.58
99.69
39.52 DMSO-d6
220 210 200 190 180 170 160 150 140 130 120 110 100 90 80 70 60 50 40 30 20 10 0
f1 (ppm)

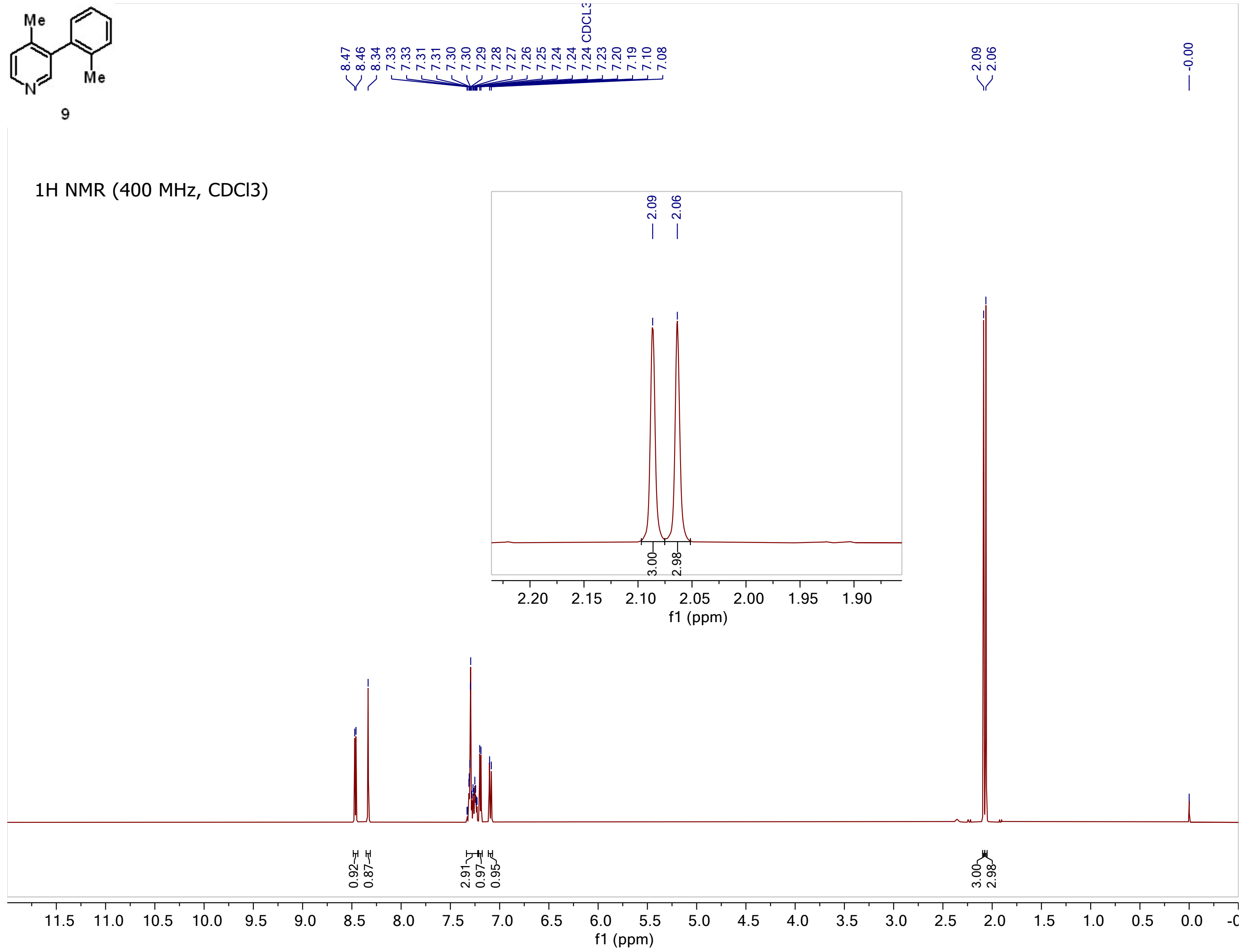
9
1H NMR (400 MHz, CDCl3)
f1 (ppm)

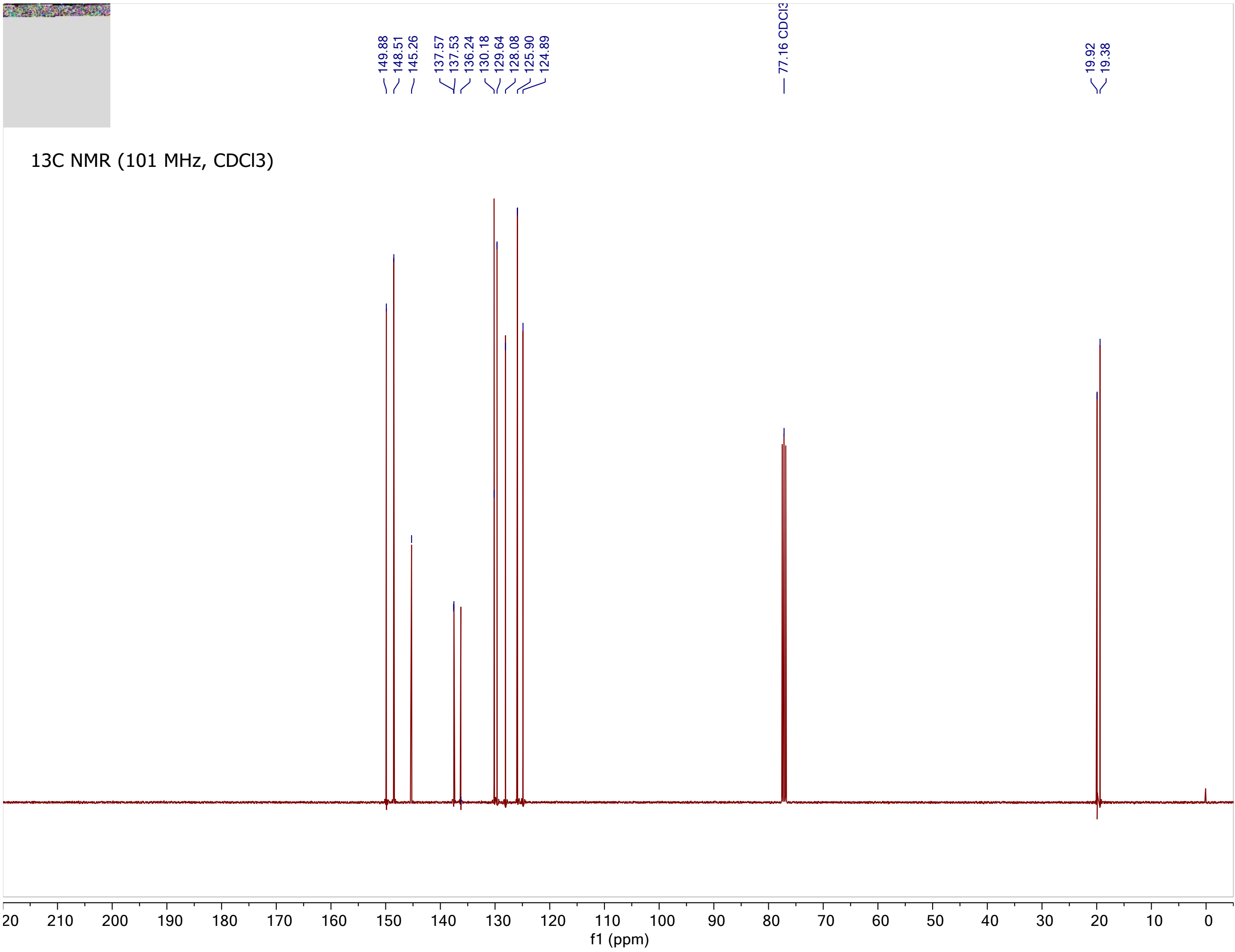

149.88
148.51
145.26
137.57
137.53
136.24
130.18
129.64
128.08
125.90
124.89
77.16 CDCl3
19.92
19.38
13C NMR (101 MHz, CDCl3)
f1 (ppm)

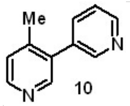

10

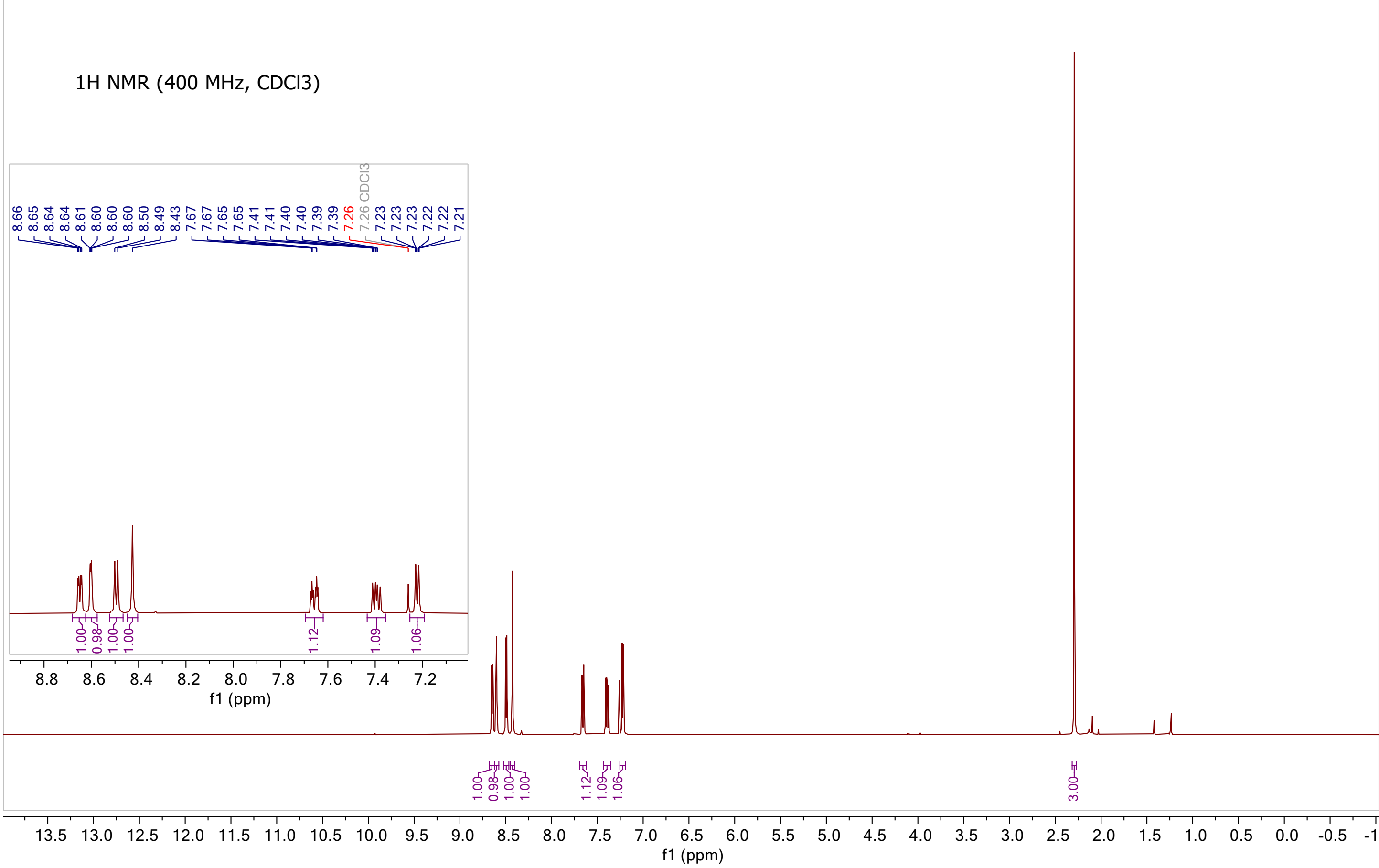

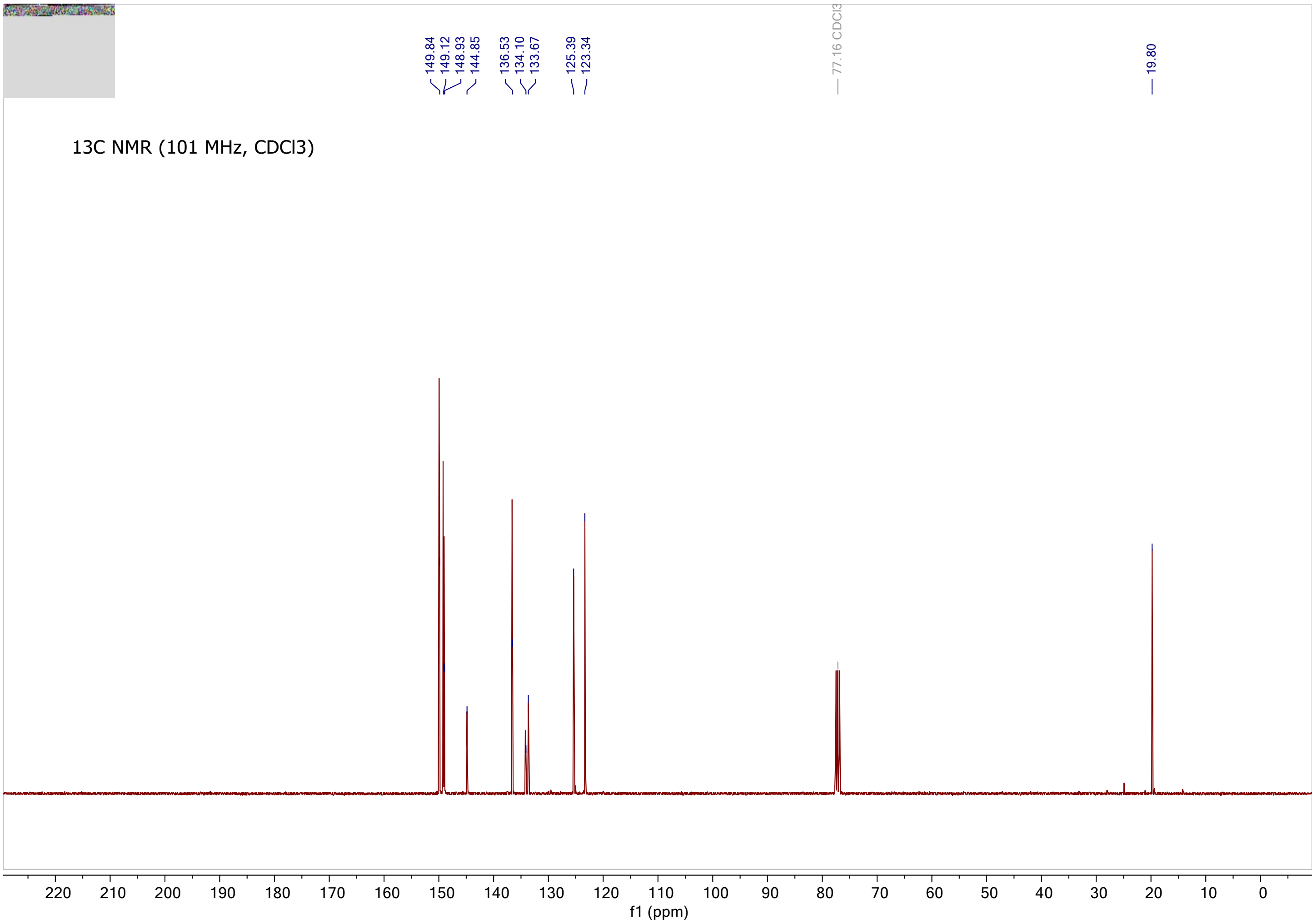

149.84
149.12
148.93
144.85
136.53
134.10
133.67
125.39
123.34
77.16 CDCl3
19.80
13C NMR (101 MHz, CDCl3)
220 210 200 190 180 170 160 150 140 130 120 110 100 90 80 70 60 50 40 30 20 10 0
f1 (ppm)

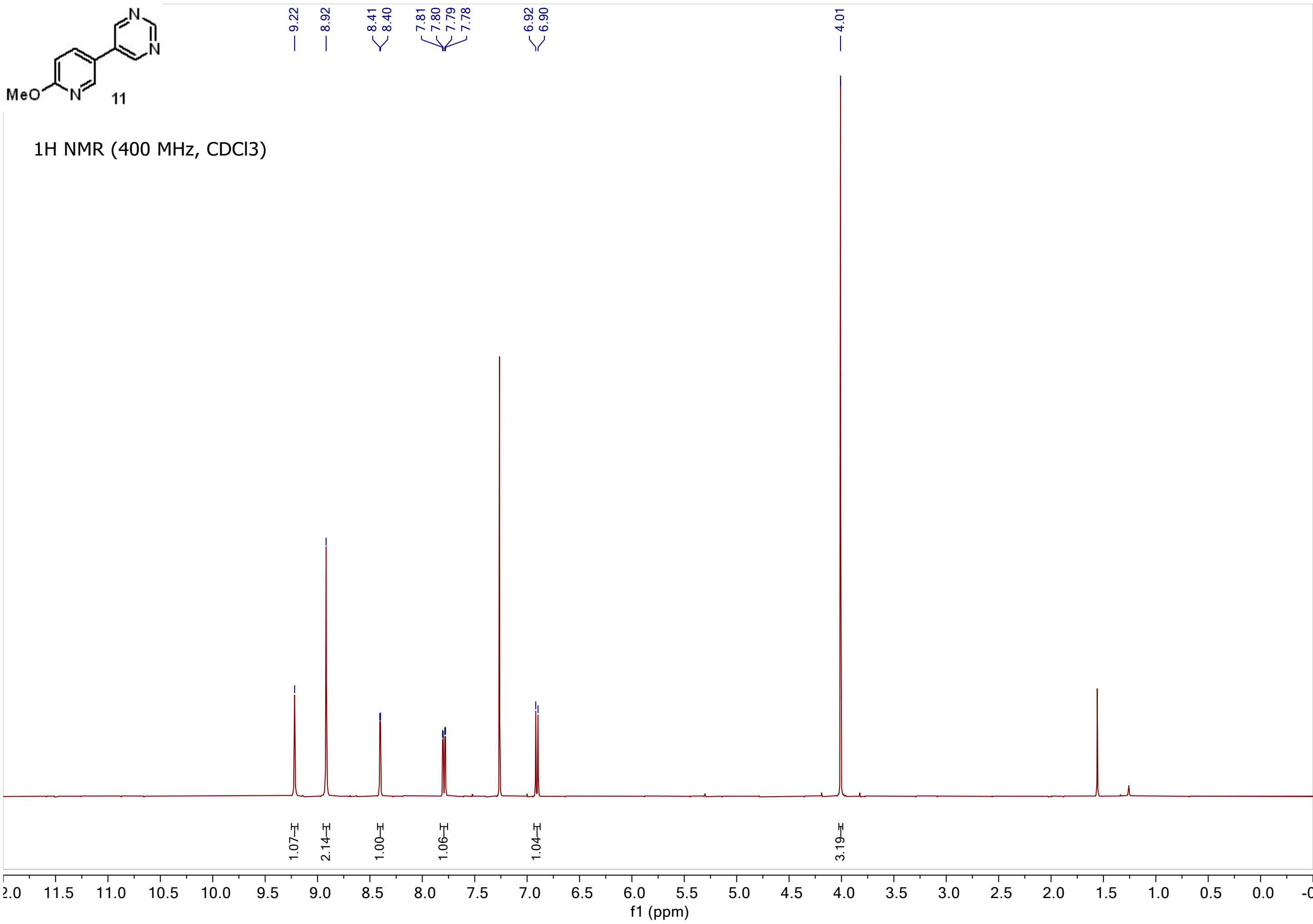
11
1H NMR (400 MHz, CDCl3)
9.22
8.92
8.41
8.40
7.81
7.80
7.79
7.78
6.92
6.90
4.01
1.07
2.14
1.00
1.06
1.04
3.19
f1 (ppm)

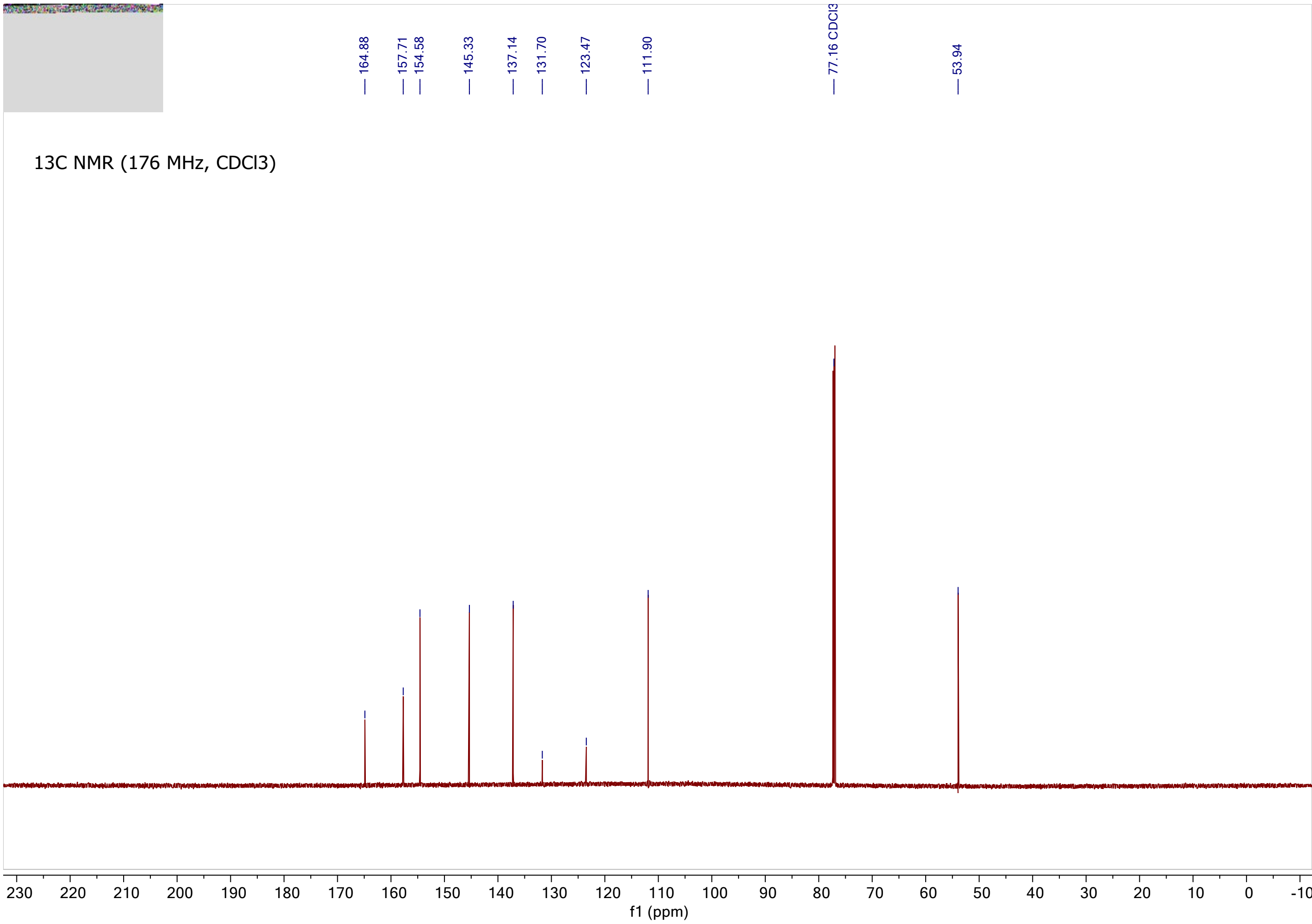

164.88
157.71
154.58
145.33
137.14
131.70
123.47
111.90
77.16 CDCl3
53.94
13C NMR (176 MHz, CDCl3)
230 220 210 200 190 180 170 160 150 140 130 120 110 100 90 80 70 60 50 40 30 20 10 0 -10
f1 (ppm)

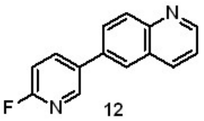

12

1H NMR (400 MHz, CDCl3)

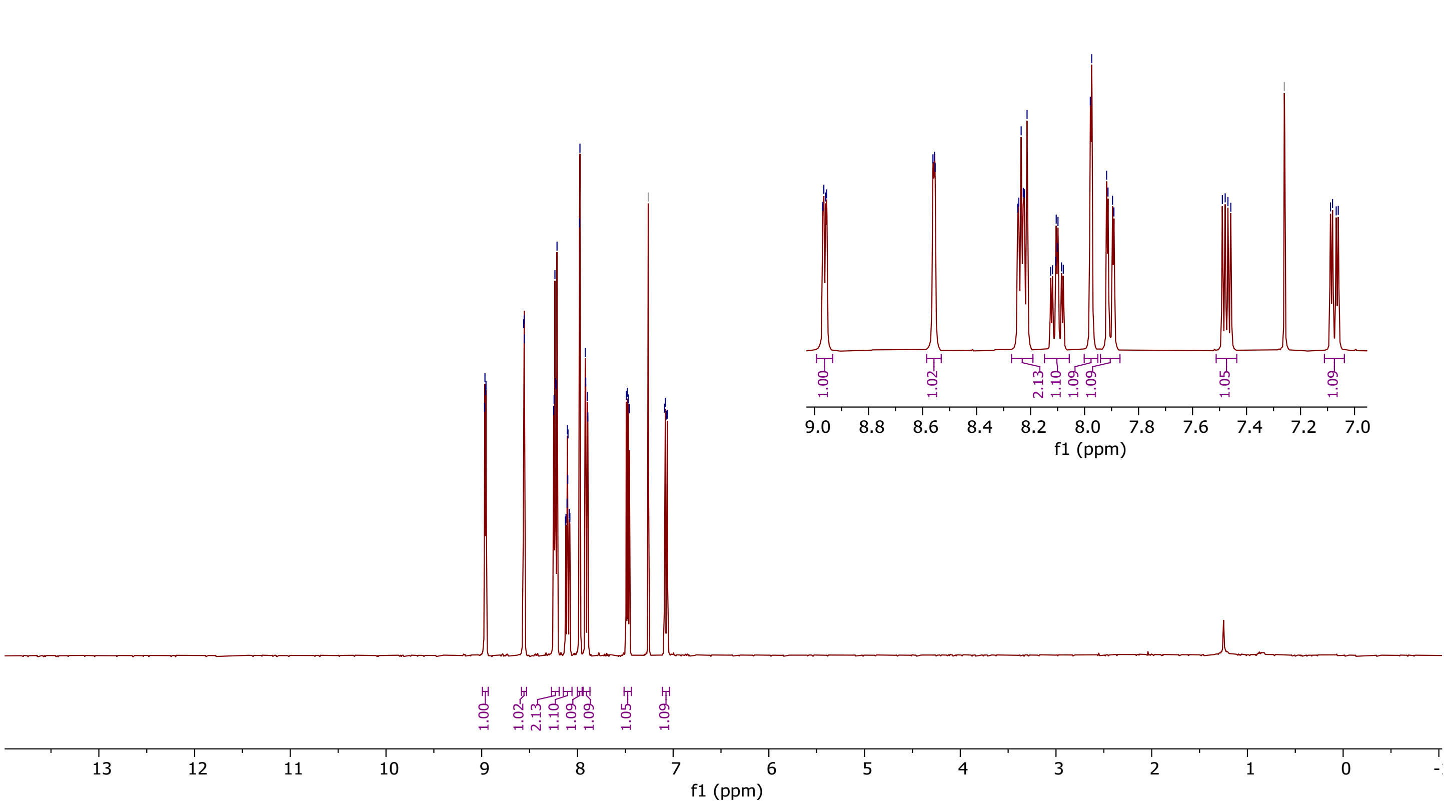

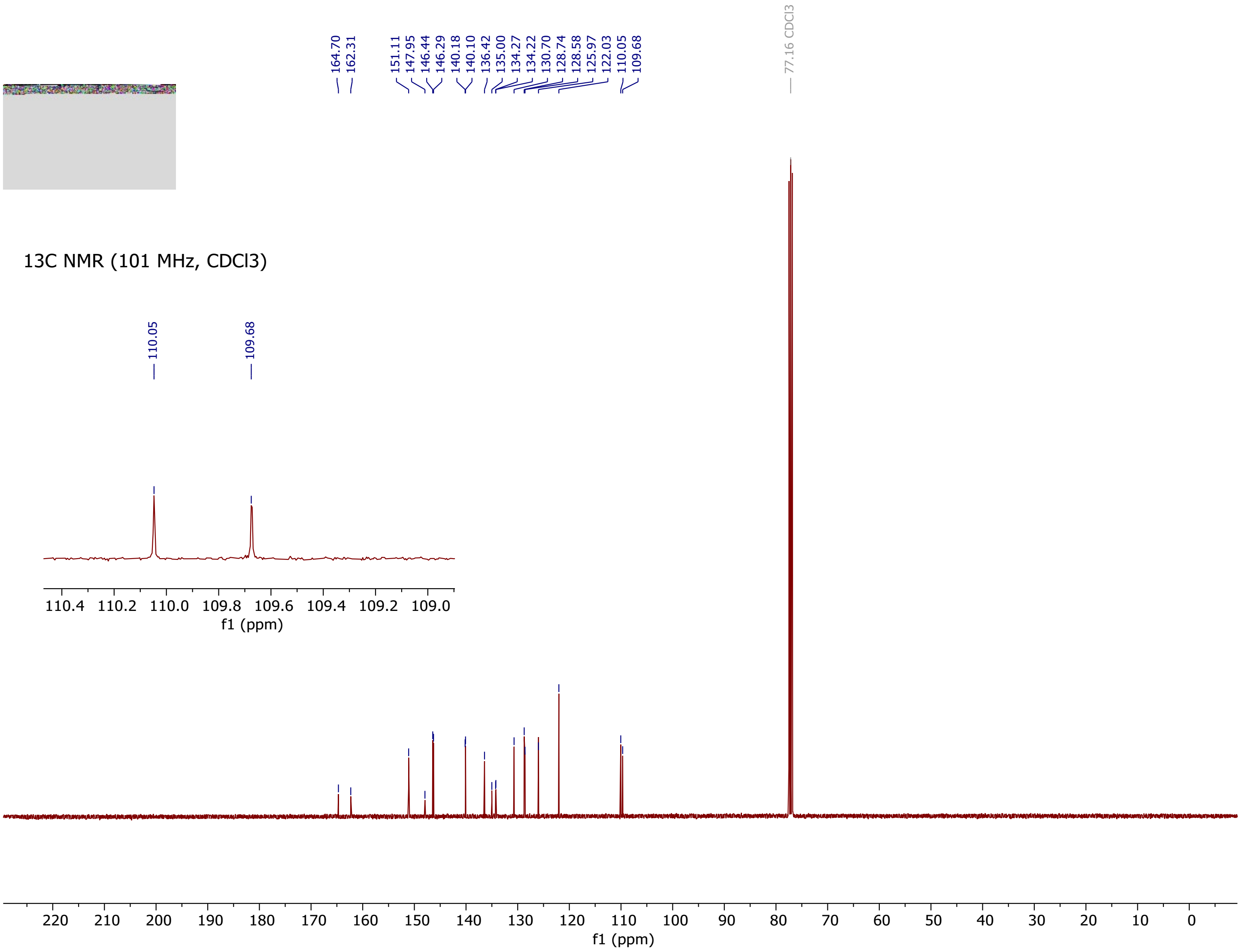
164.70
162.31
151.11
147.95
146.44
146.29
140.18
140.10
136.42
135.00
134.27
134.22
130.70
128.74
128.58
125.97
122.03
110.05
109.68
77.16 CDCl3
13C NMR (101 MHz, CDCl3)
110.05
109.68
110.4 110.2 110.0 109.8 109.6 109.4 109.2 109.0
f1 (ppm)
220 210 200 190 180 170 160 150 140 130 120 110 100 90 80 70 60 50 40 30 20 10 0
f1 (ppm)

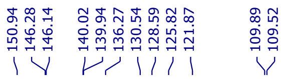

13C NMR, DEPT-135 (101 MHz, CDCl3)

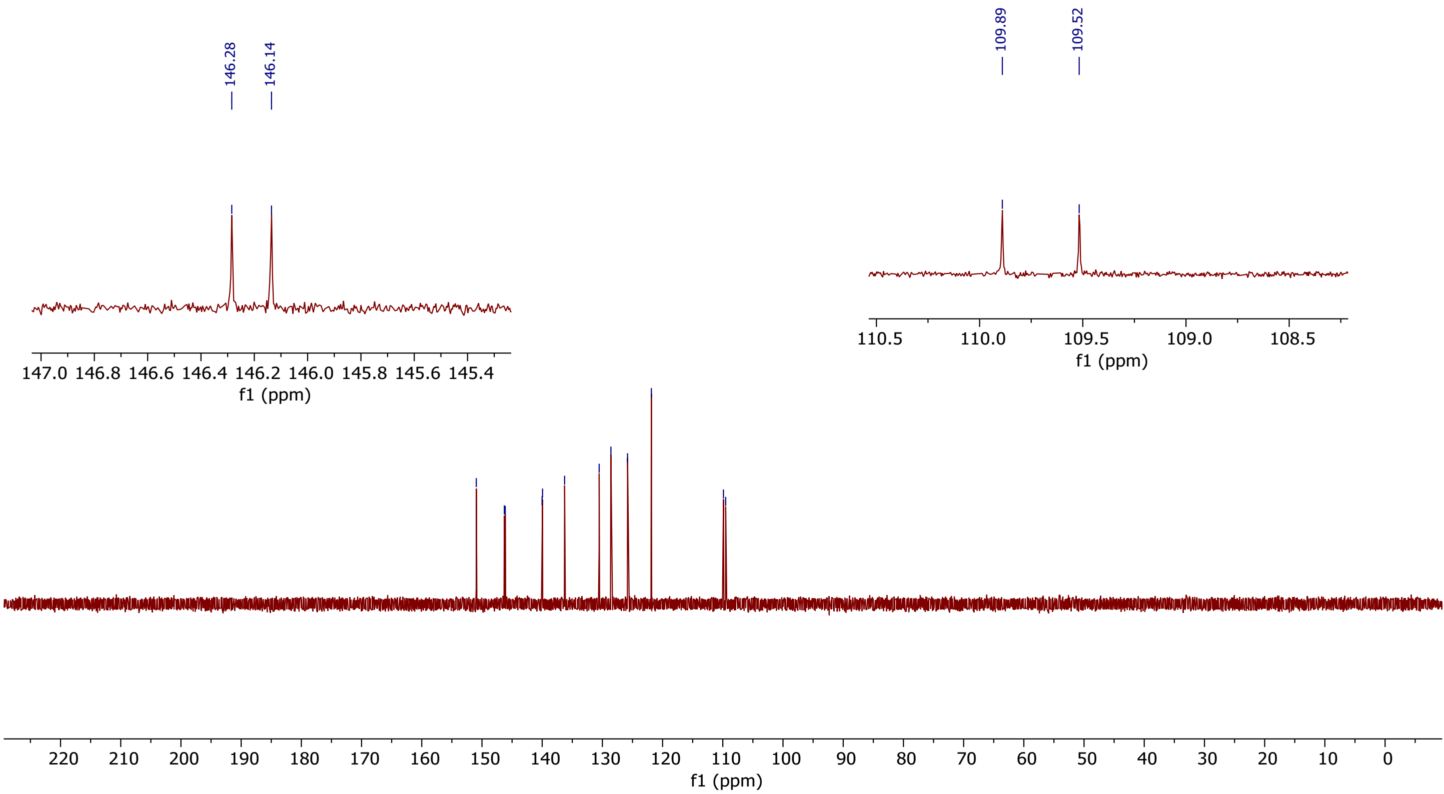

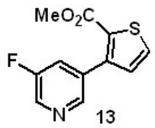

13

1H NMR (400 MHz, DMSO-d6)

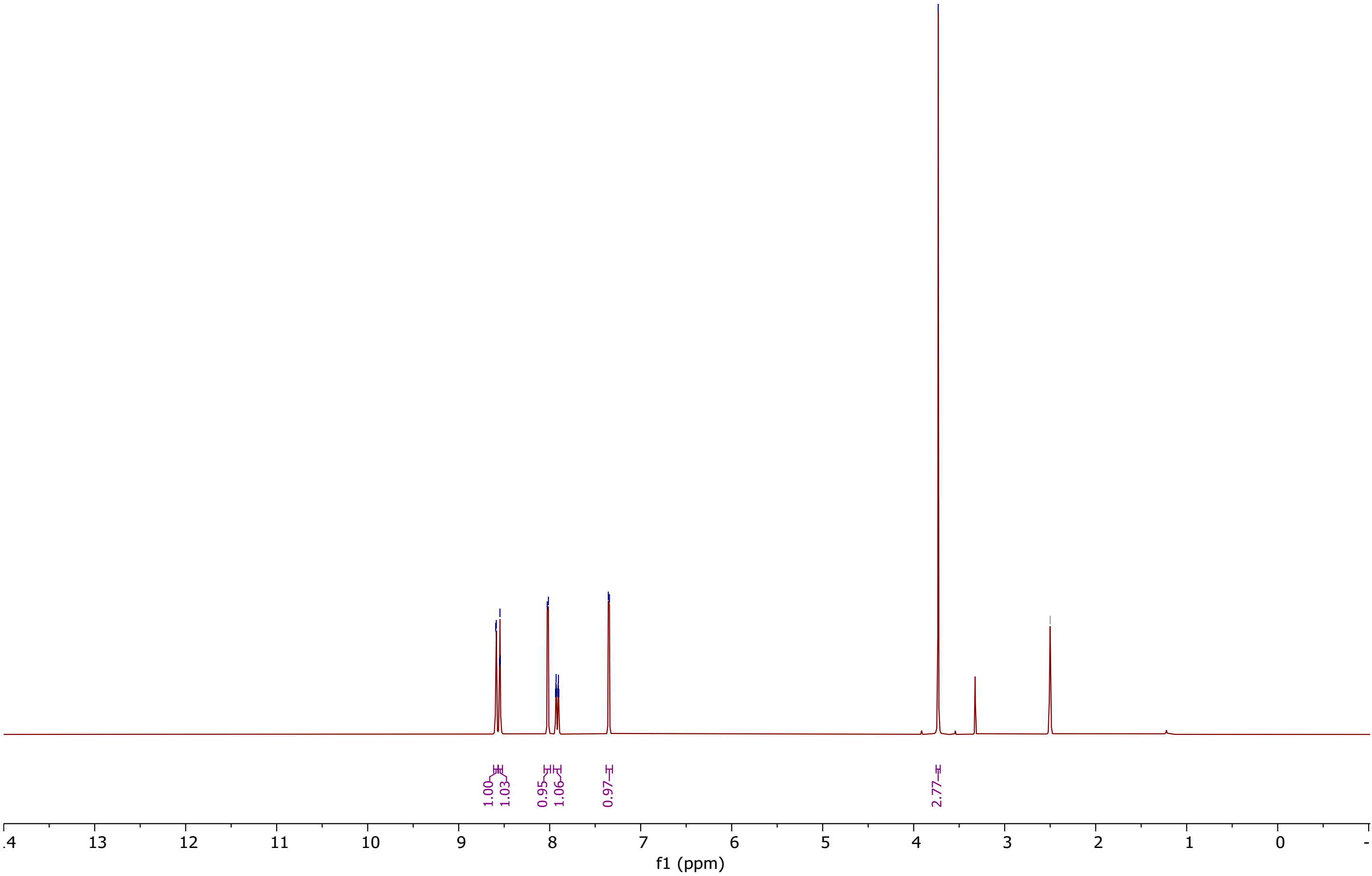

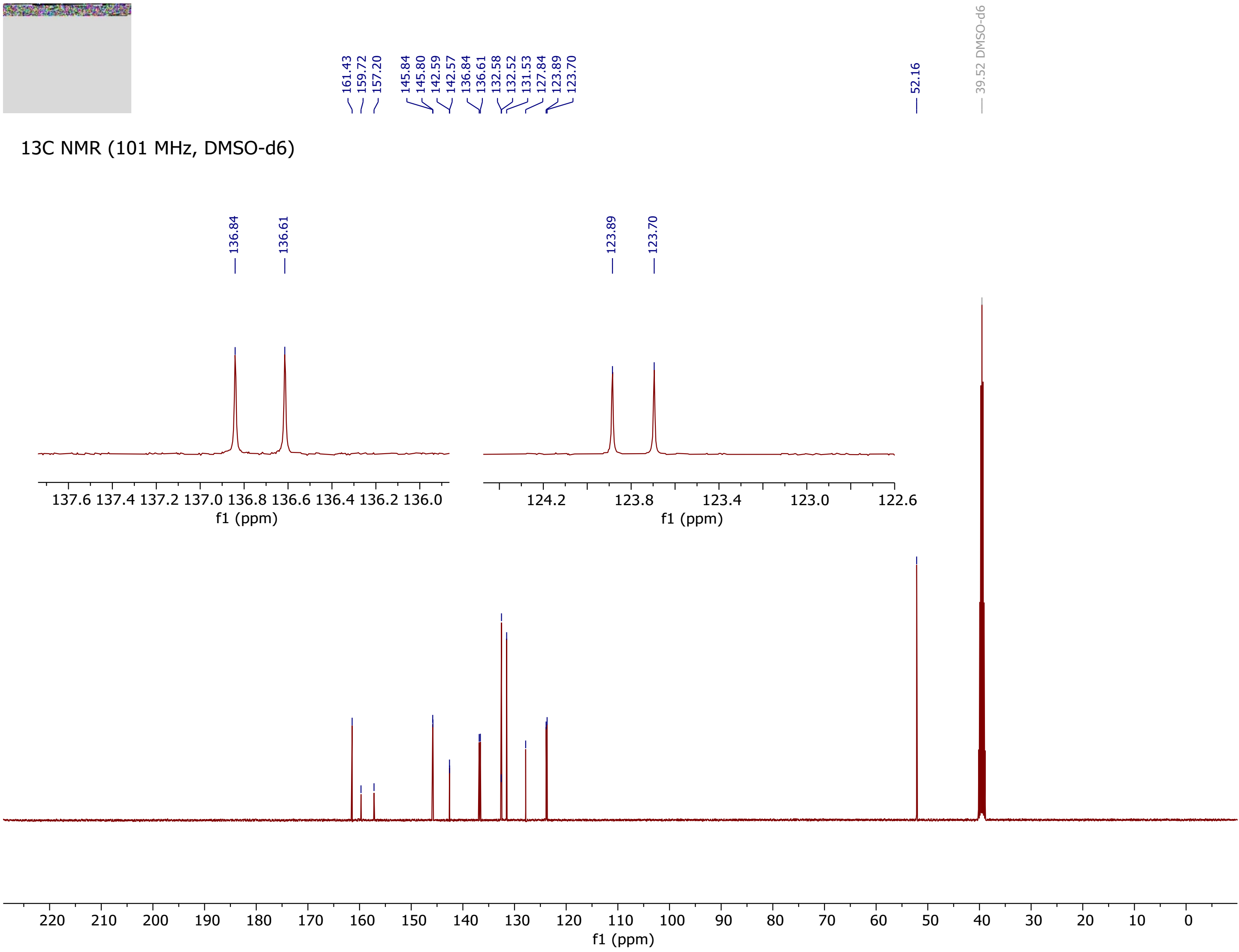
13C NMR (101 MHz, DMSO-d6)
161.43
159.72
157.20
145.84
145.80
142.59
142.57
136.84
136.61
132.58
132.52
131.53
127.84
123.89
123.70
52.16
39.52 DMSO-d6
f1 (ppm)

1H NMR (400 MHz, CDCl3)

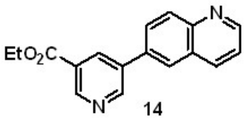

14

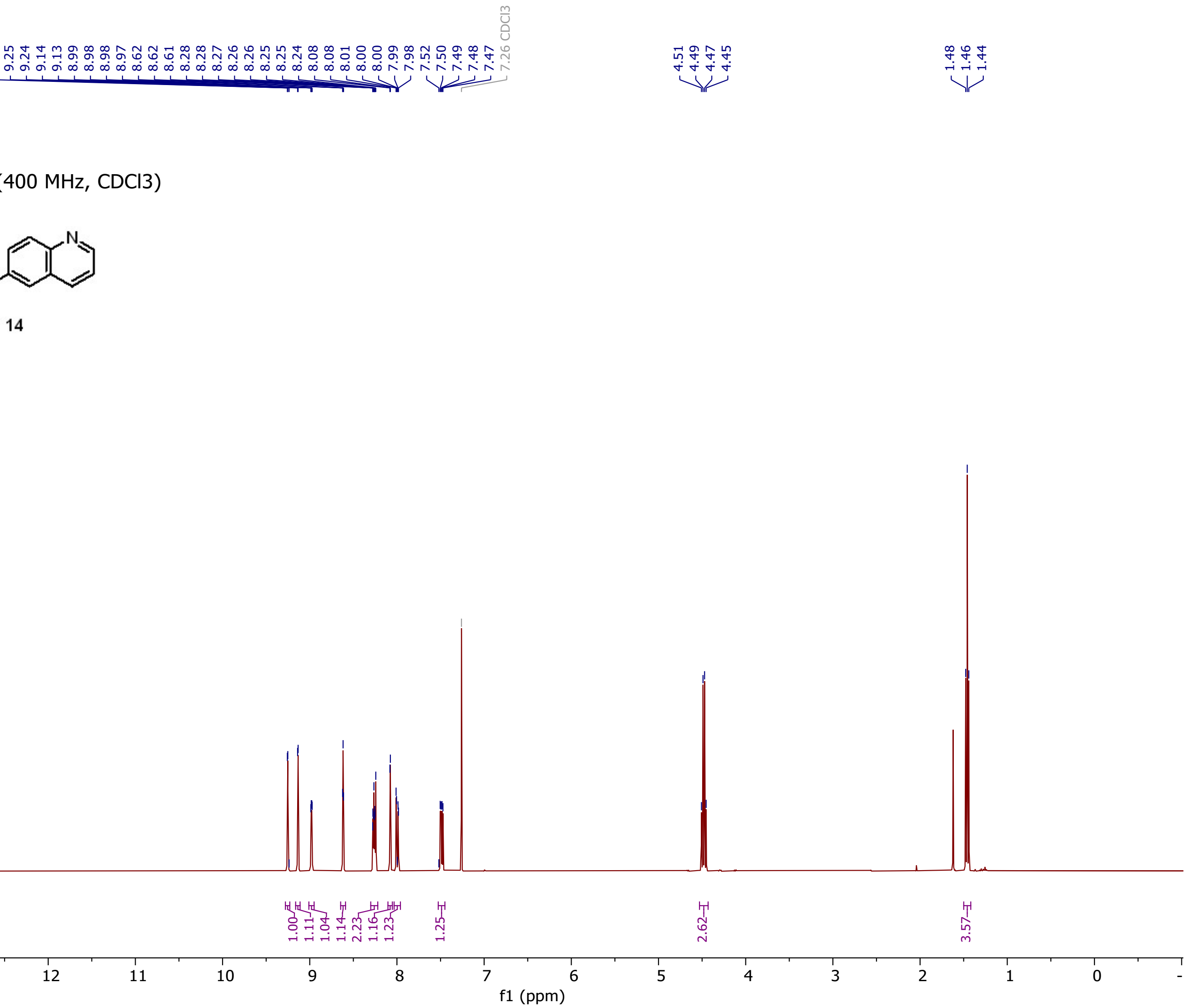

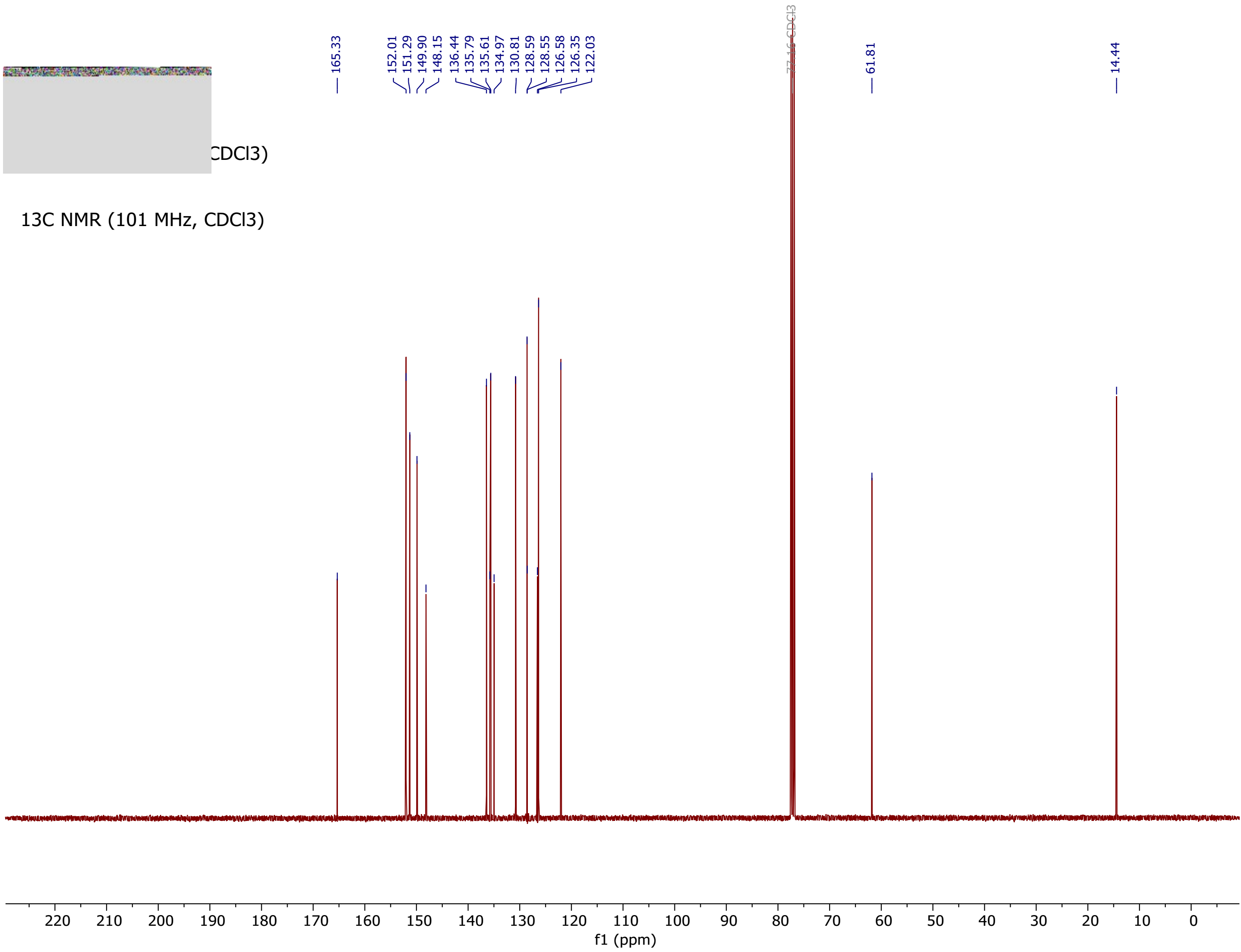
CDCl3)
13C NMR (101 MHz, CDCl3)
165.33
152.01
151.29
149.90
148.15
136.44
135.79
135.61
134.97
130.81
128.59
128.55
126.58
126.35
122.03
77.16 CDCl3
61.81
14.44
220 210 200 190 180 170 160 150 140 130 120 110 100 90 80 70 60 50 40 30 20 10 0
f1 (ppm)

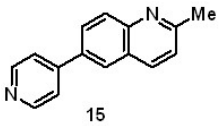

15

1H NMR (400 MHz, DMSO-d6)

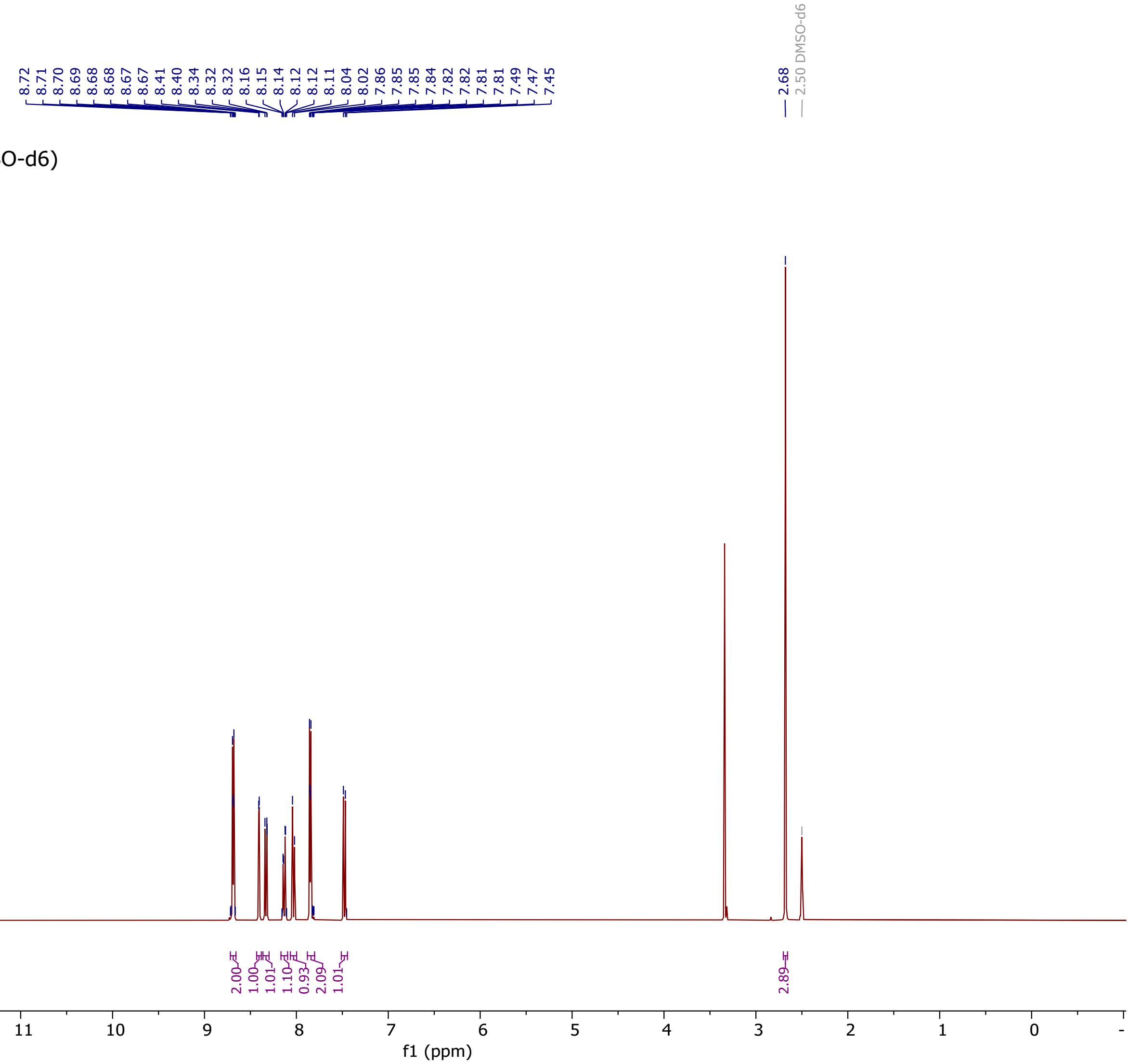

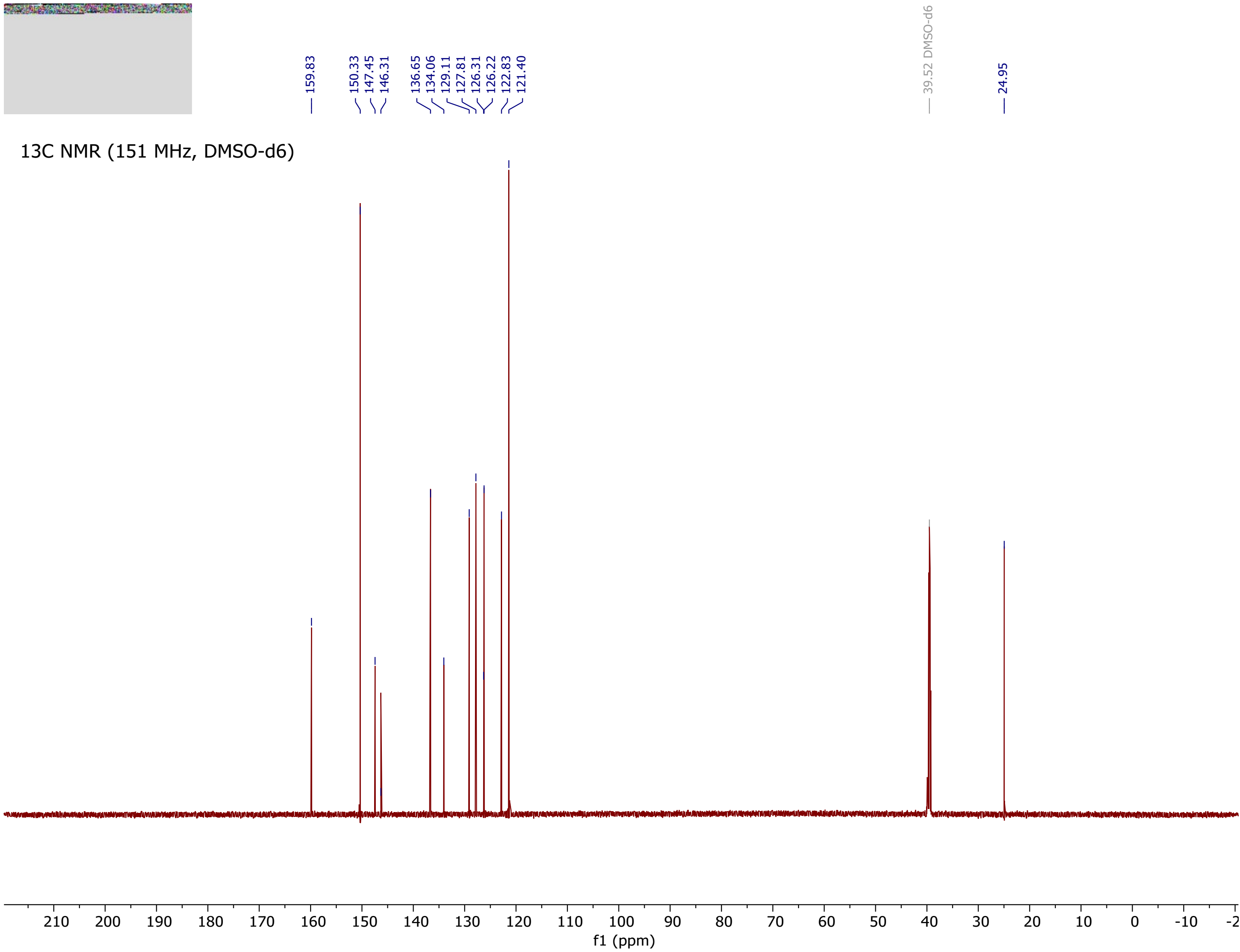
13C NMR (151 MHz, DMSO-d6)
159.83
150.33
147.45
146.31
136.65
134.06
129.11
127.81
126.31
126.22
122.83
121.40
39.52 DMSO-d6
24.95
210 200 190 180 170 160 150 140 130 120 110 100 90 80 70 60 50 40 30 20 10 0 -10
f1 (ppm)

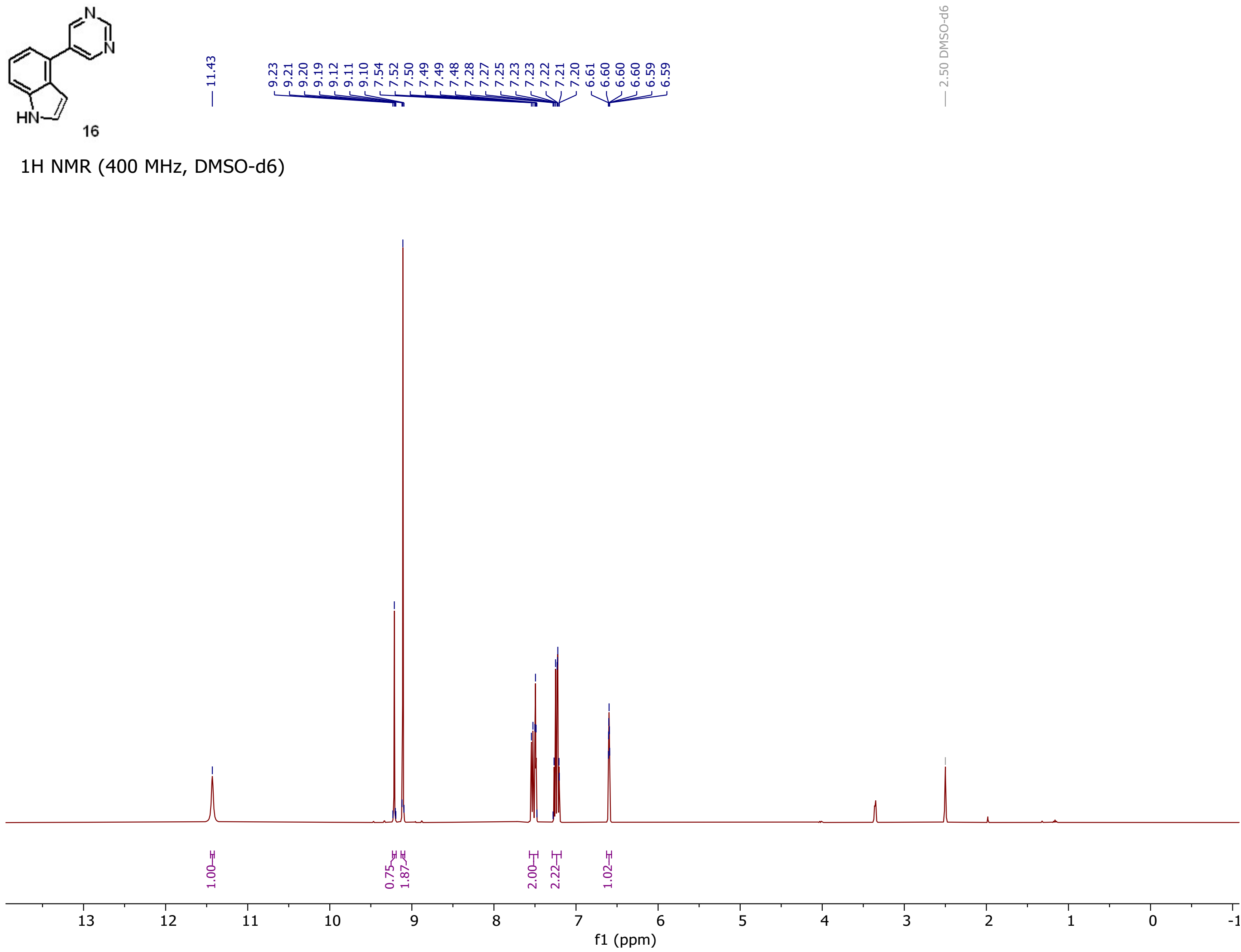

1H NMR (400 MHz, DMSO-d6)

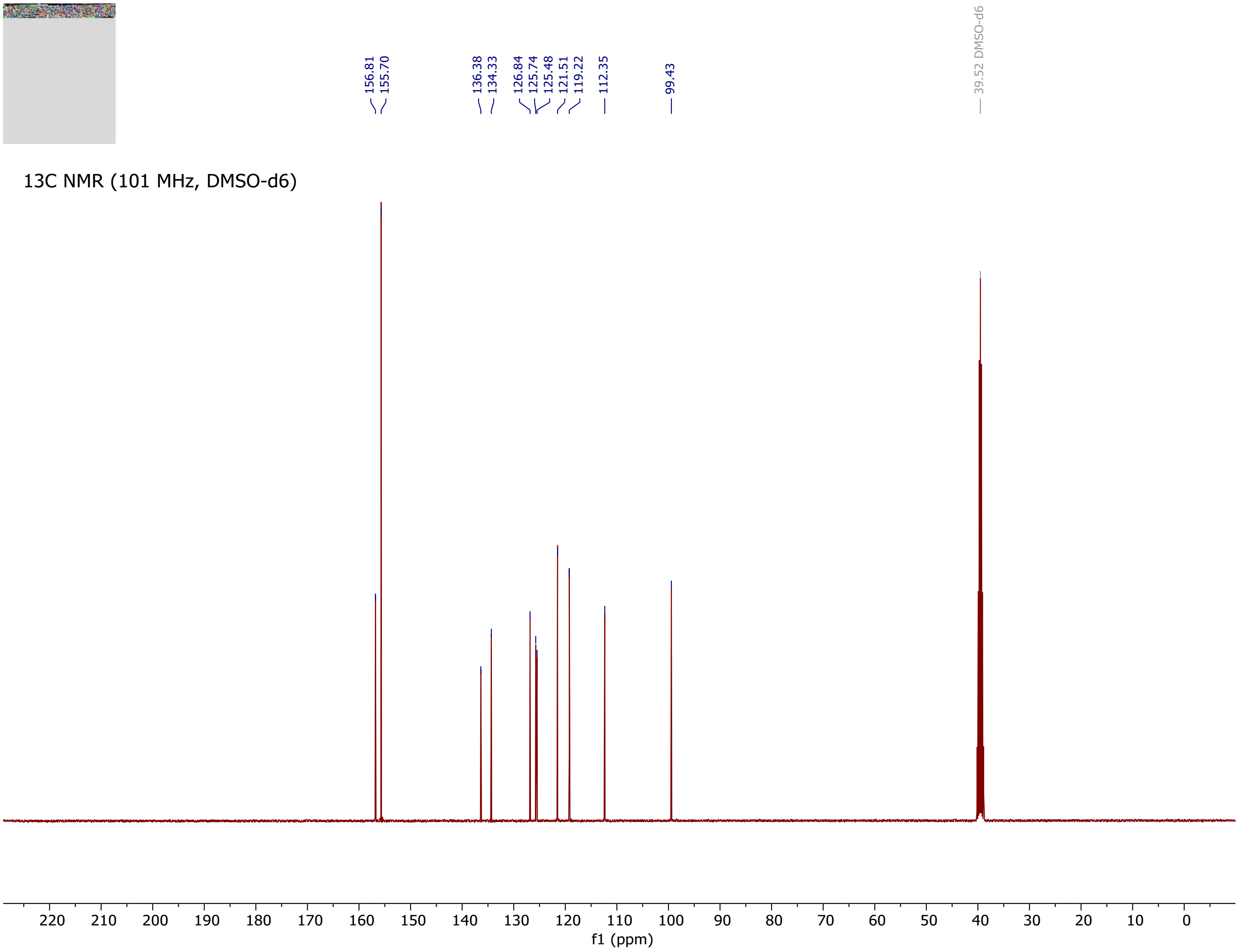
13C NMR (101 MHz, DMSO-d6)
156.81
155.70
136.38
134.33
126.84
125.74
125.48
121.51
119.22
112.35
99.43
39.52 DMSO-d6
220 210 200 190 180 170 160 150 140 130 120 110 100 90 80 70 60 50 40 30 20 10 0
f1 (ppm)

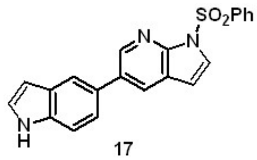

17

1H NMR (400 MHz, DMSO-d6)

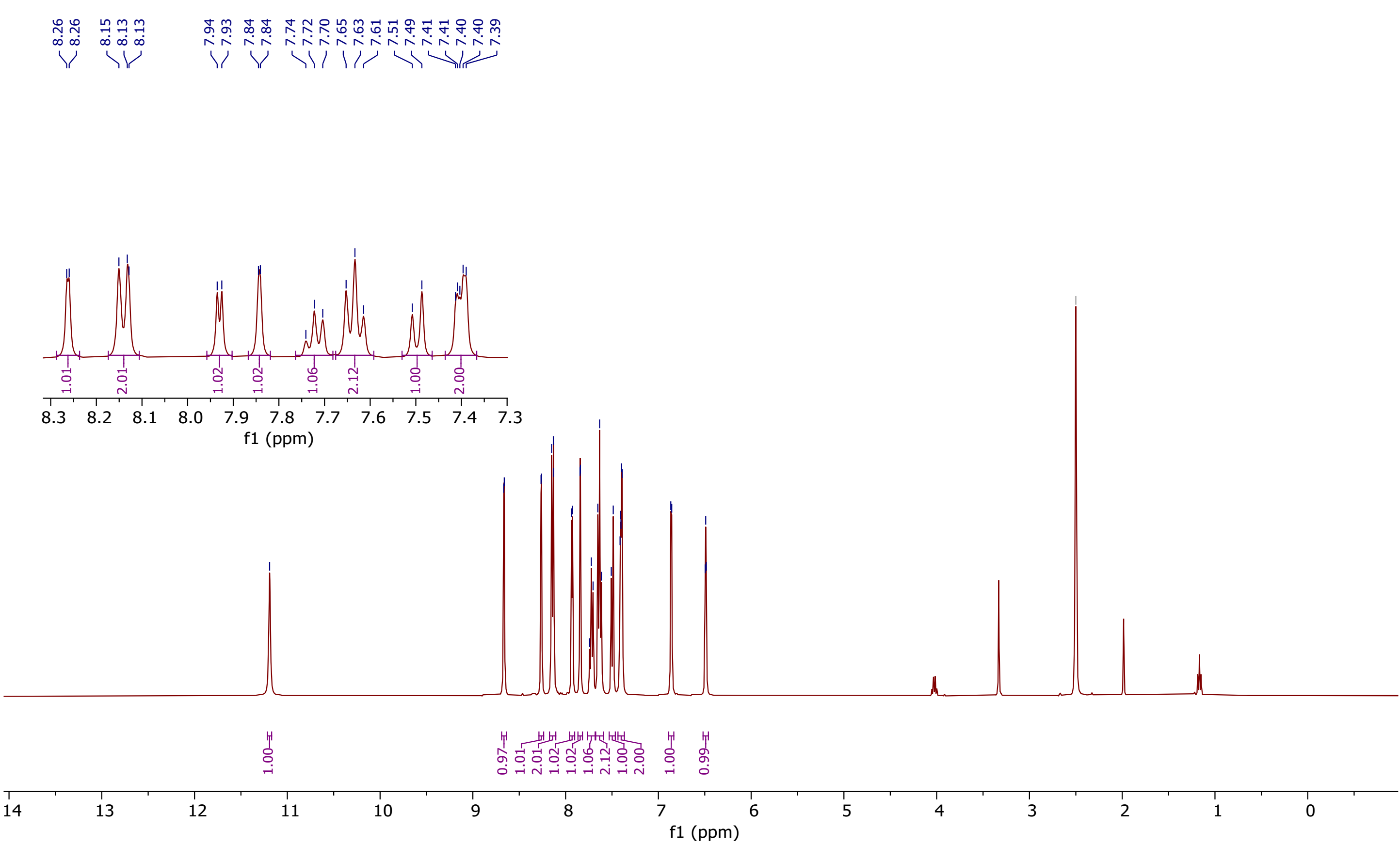

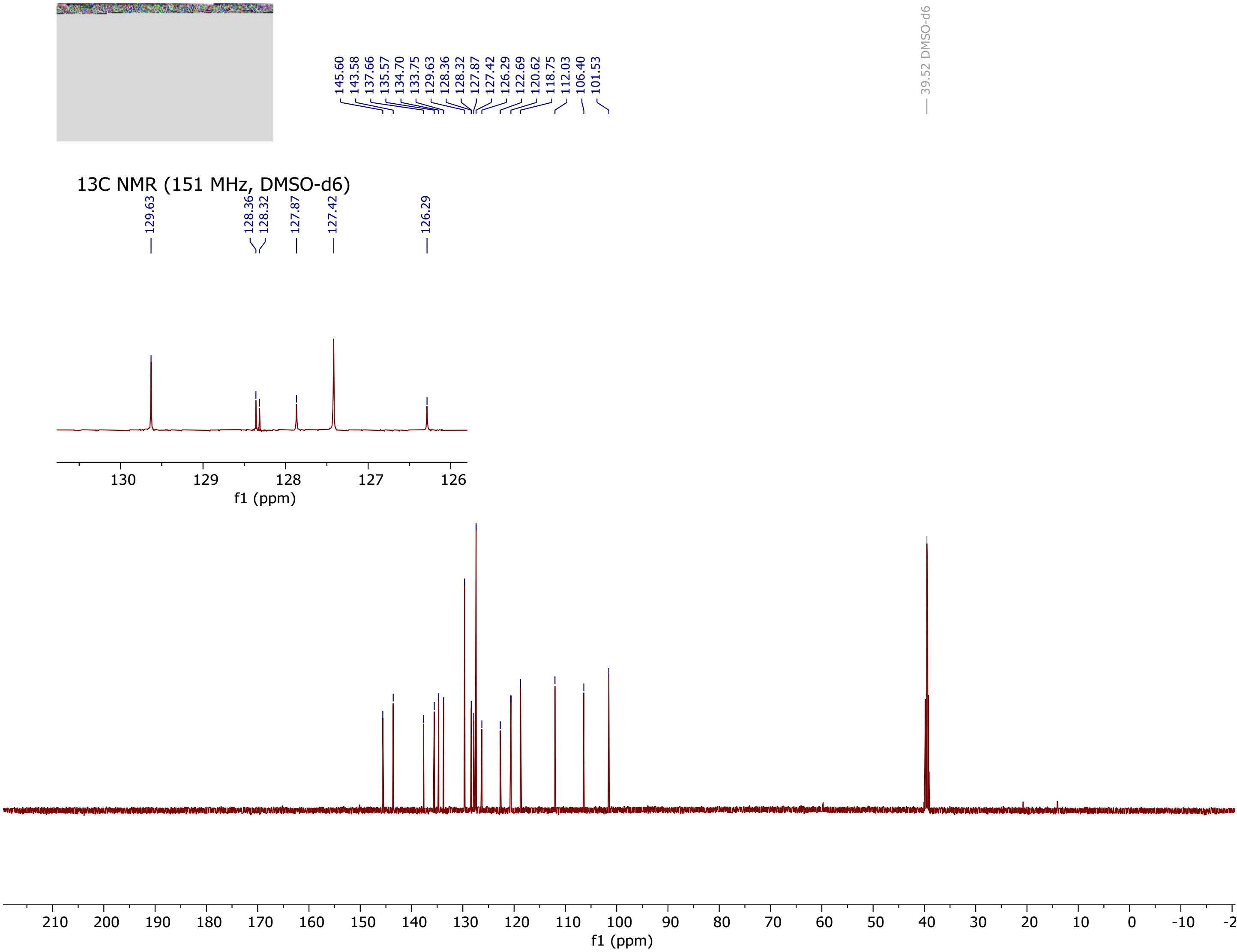
13C NMR (151 MHz, DMSO-d6)
145.60
143.58
137.66
135.57
134.70
133.75
129.63
128.36
128.32
127.87
127.42
126.29
122.69
120.62
118.75
112.03
106.40
101.53
39.52 DMSO-d6
129.63
128.36
128.32
127.87
127.42
126.29
f1 (ppm)
f1 (ppm)

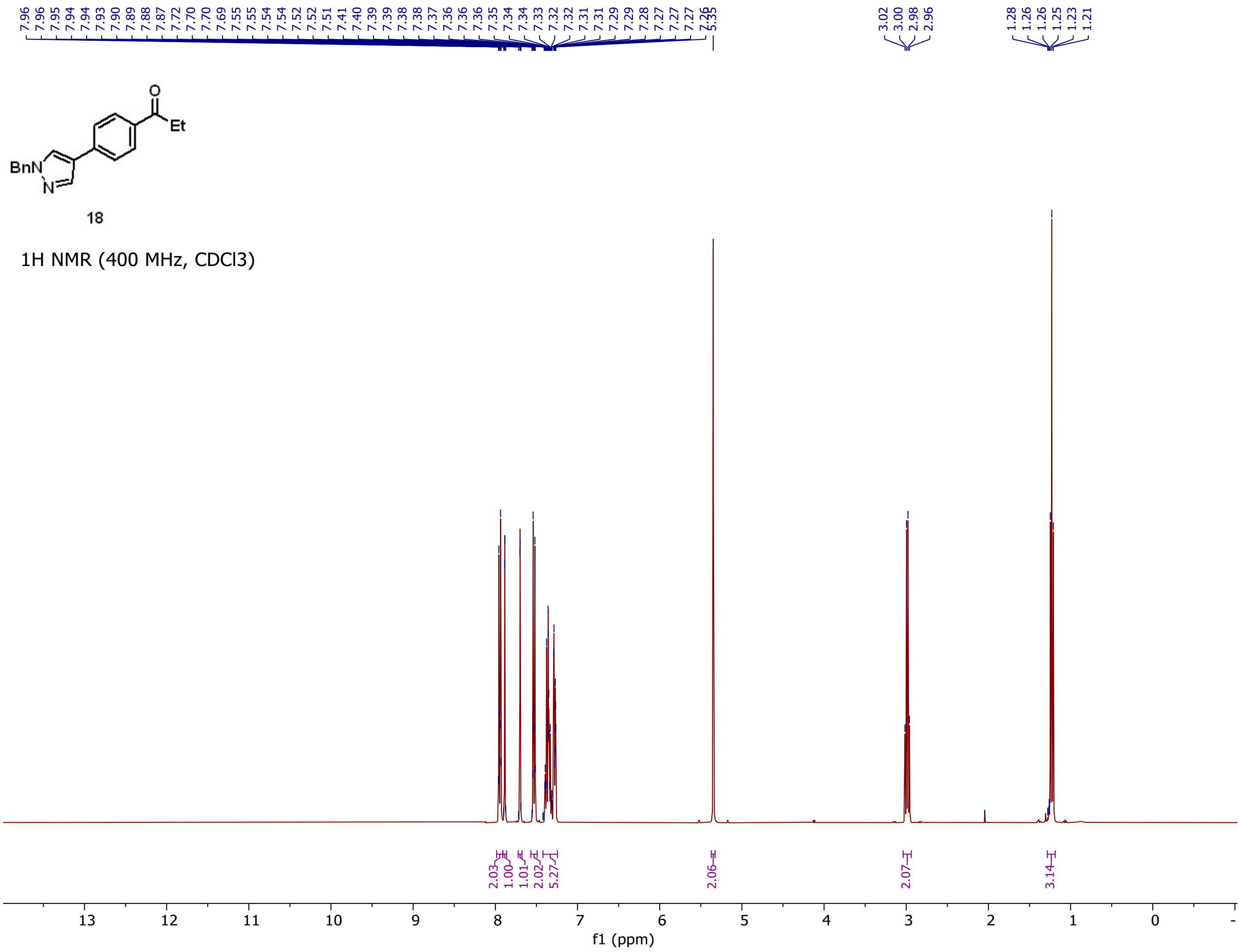
7.96 7.96 7.95 7.94 7.94 7.93 7.90 7.89 7.88 7.87 7.72 7.70 7.69 7.55 7.55 7.54 7.54 7.52 7.52 7.51 7.41 7.40 7.39 7.39 7.38 7.38 7.37 7.36 7.36 7.36 7.35 7.34 7.34 7.33 7.32 7.32 7.31 7.31 7.29 7.29 7.28 7.27 7.27 7.27 7.26 5.35
3.02 3.00 2.98 2.96
1.28 1.26 1.25 1.23 1.21
O
Et
BnN
N
18
1H NMR (400 MHz, CDCl3)
2.03
1.00
1.01
2.02
5.27
2.06
2.07
3.14
13 12 11 10 9 8 7 6 5 4 3 2 1 0
f1 (ppm)

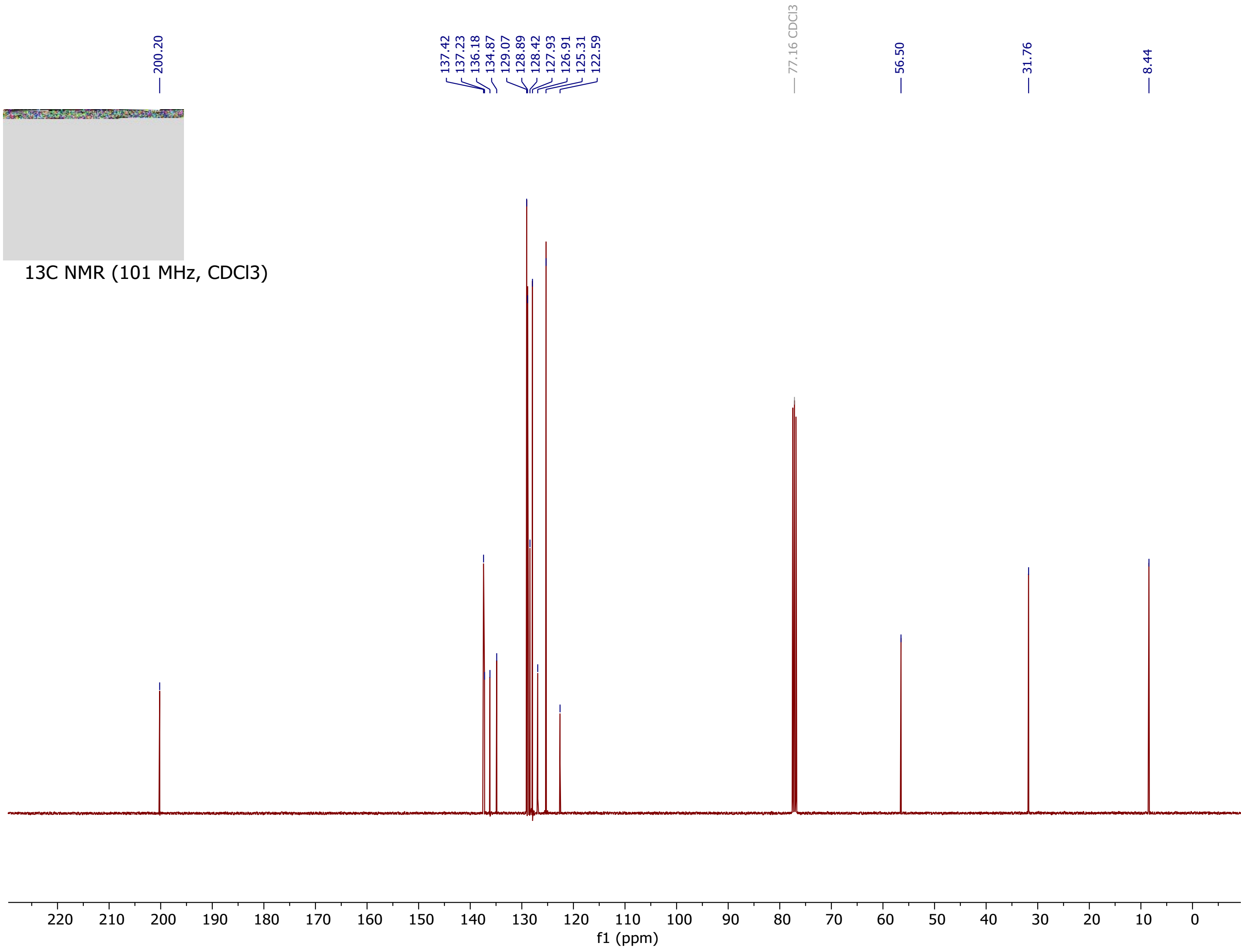
200.20
137.42
137.23
136.18
134.87
129.07
128.89
128.42
127.93
126.91
125.31
122.59
77.16 CDCl3
56.50
31.76
8.44
13C NMR (101 MHz, CDCl3)
220 210 200 190 180 170 160 150 140 130 120 110 100 90 80 70 60 50 40 30 20 10 0
f1 (ppm)

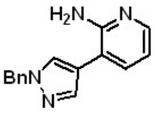

19

1H NMR (400 MHz, DMSO-d6)

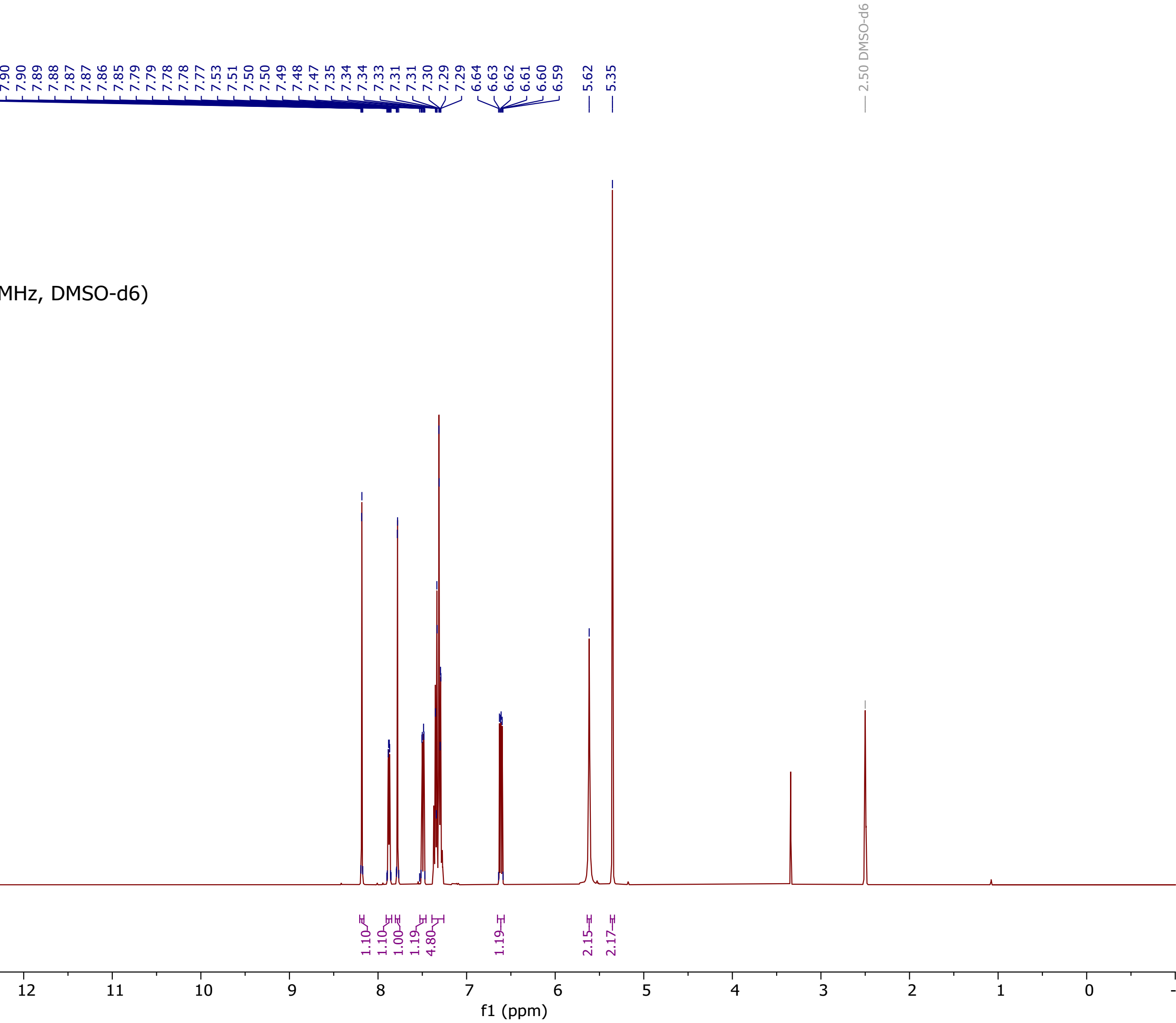

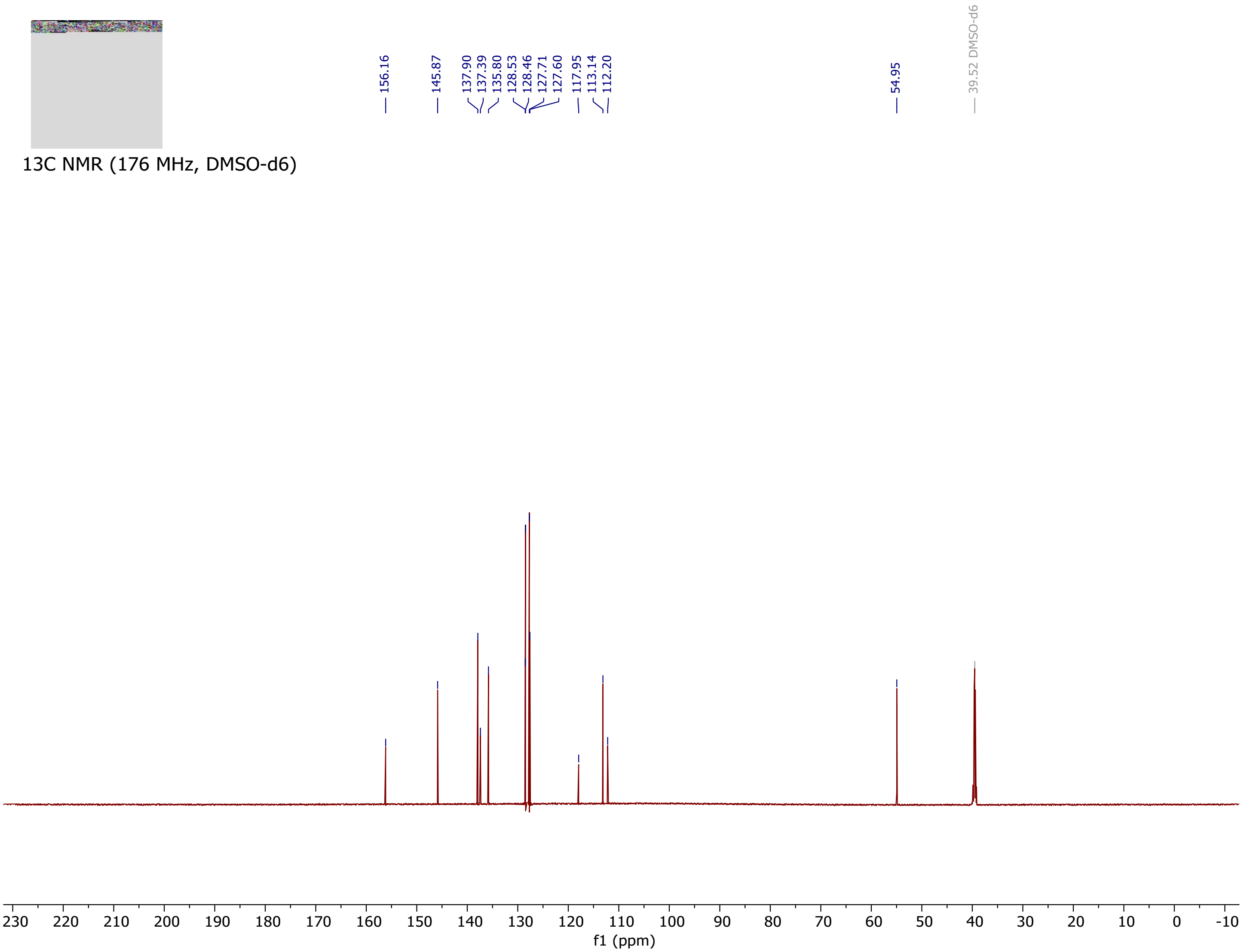

13C NMR (176 MHz, DMSO-d6)

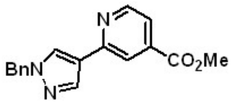

20

1H NMR (400 MHz, CDCl3)

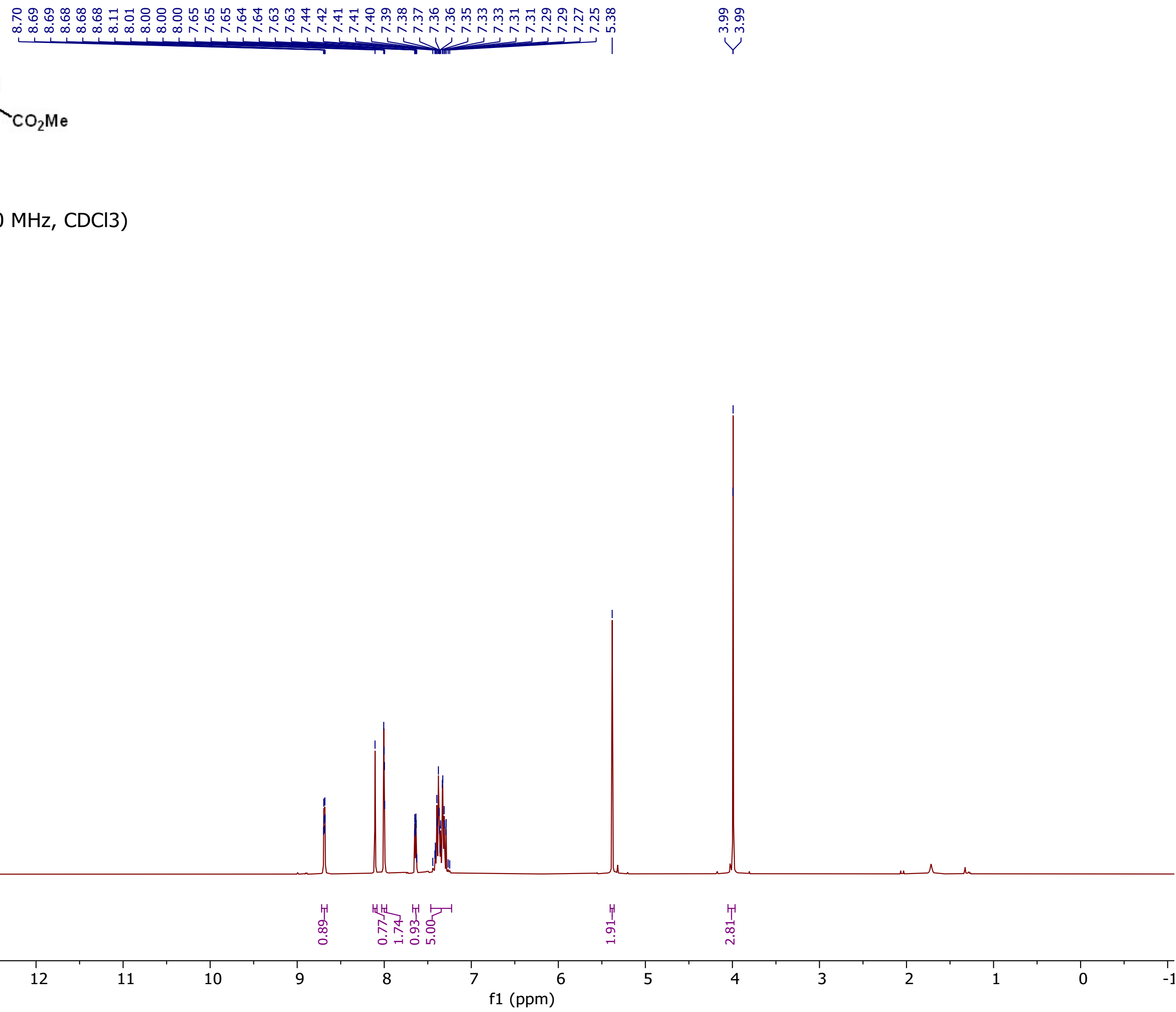

13C NMR (176 MHz, CDCl3)

13C NMR (176 MHz, CDCl3)

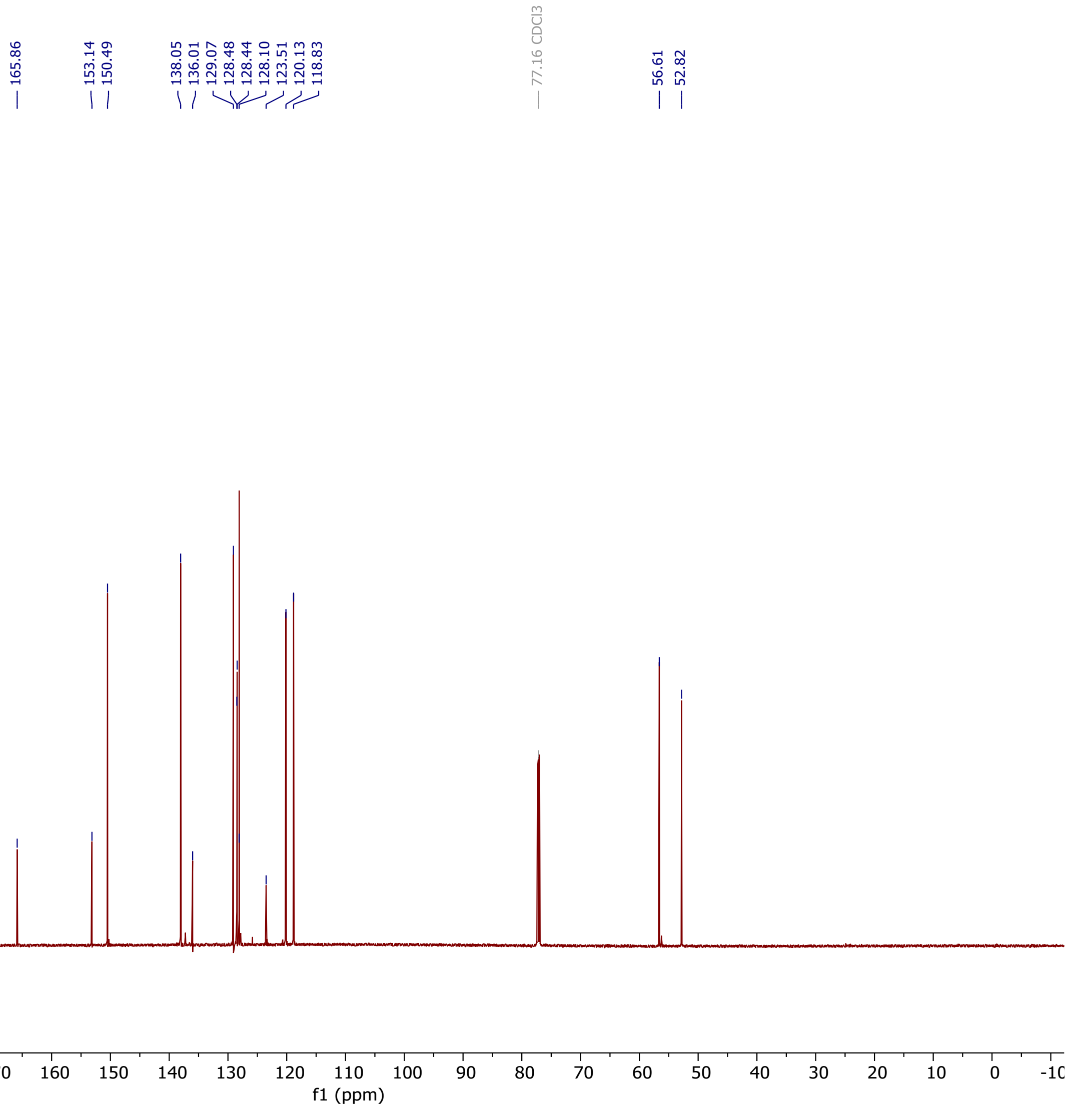

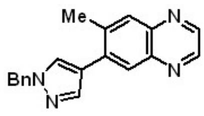

21

1H NMR (400 MHz, DMSO-d6)

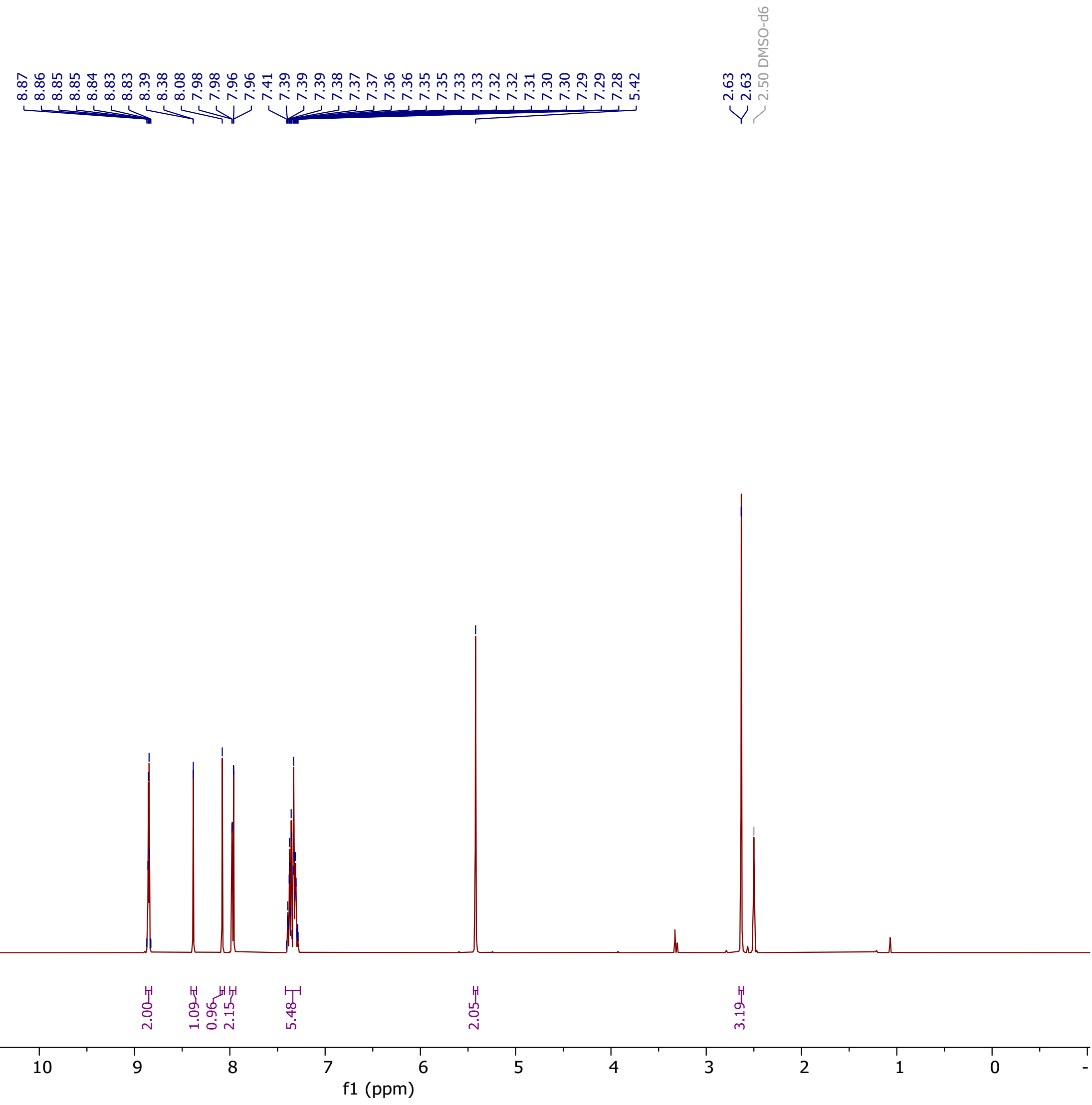

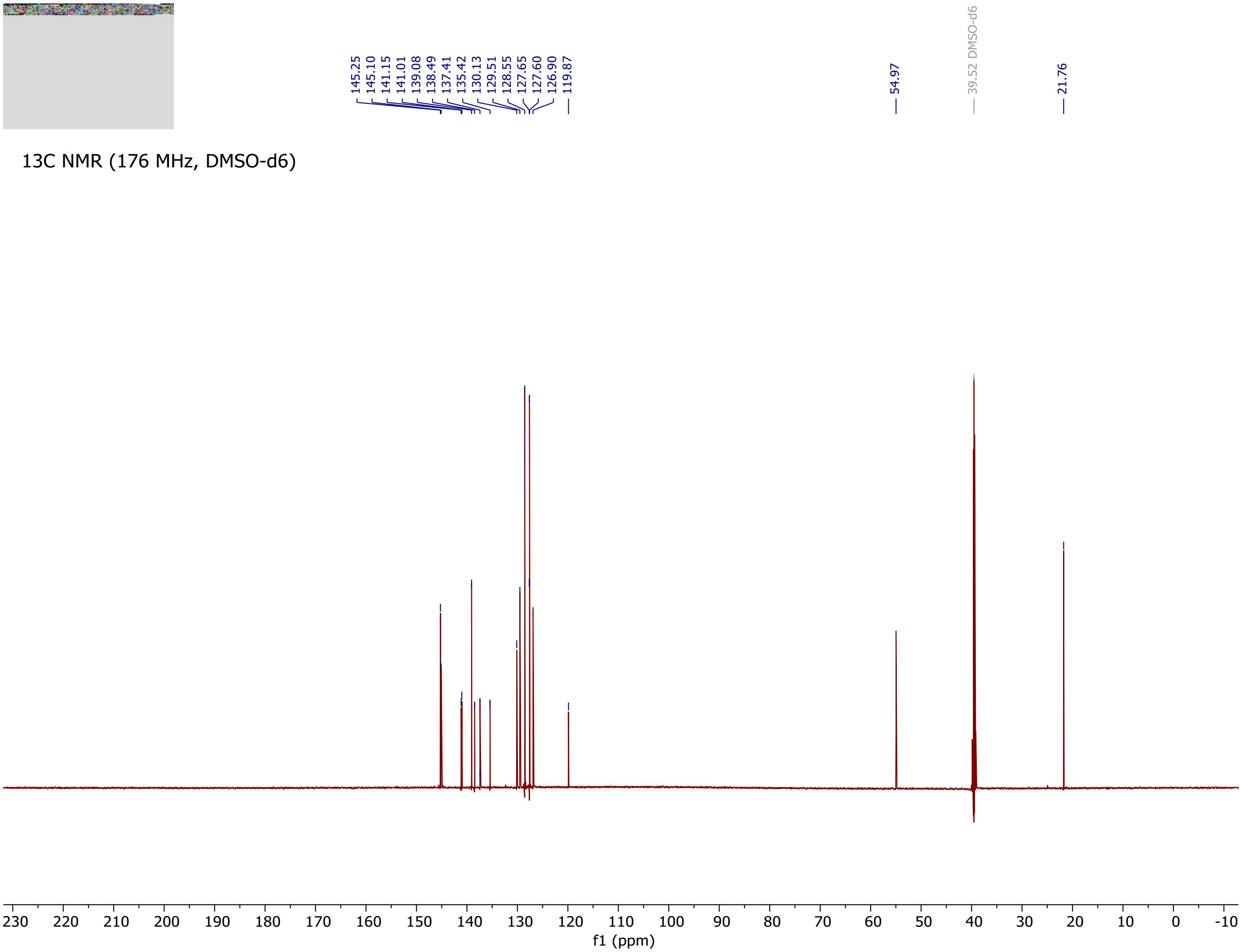

13C NMR (176 MHz, DMSO-d6)

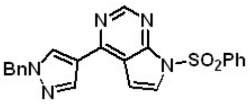

22

1H NMR (400 MHz, DMSO-d6)

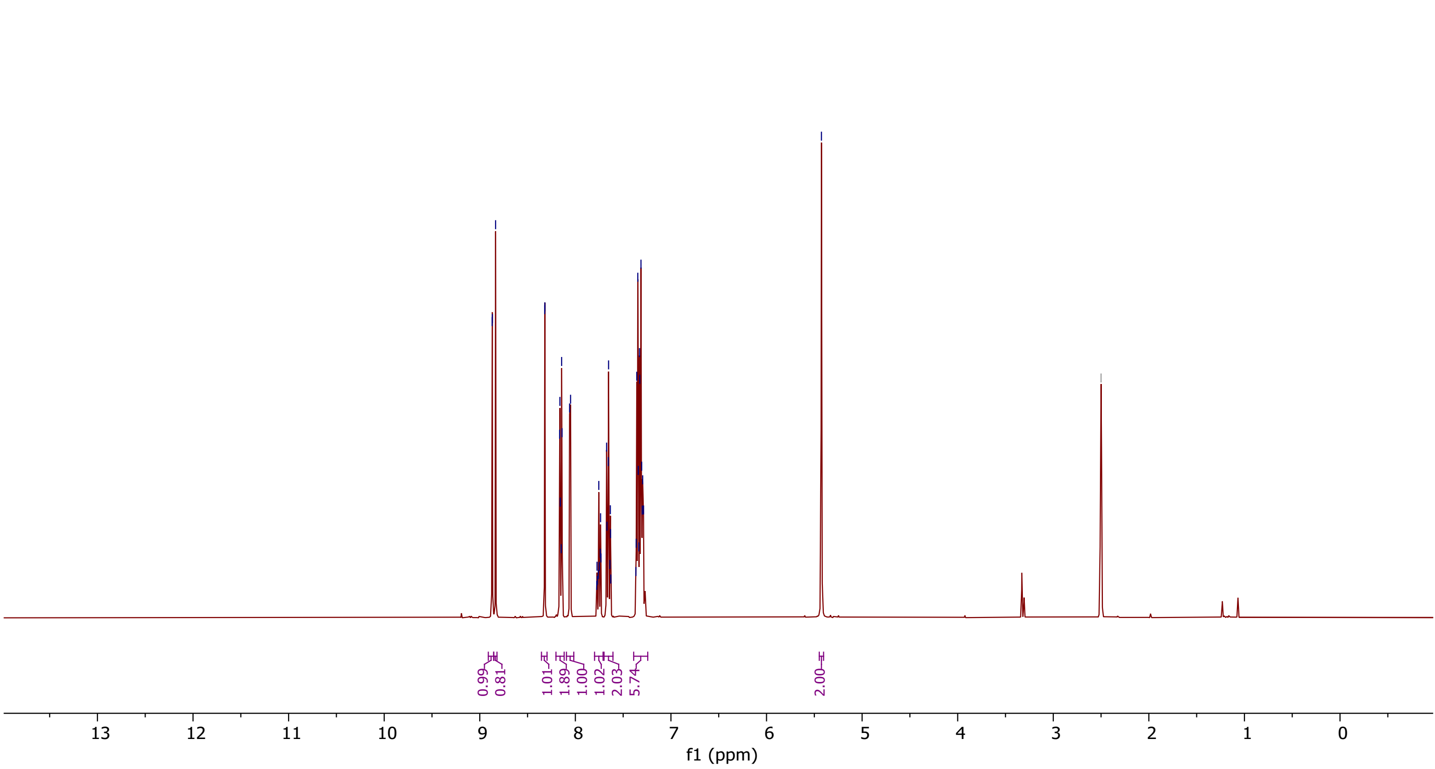

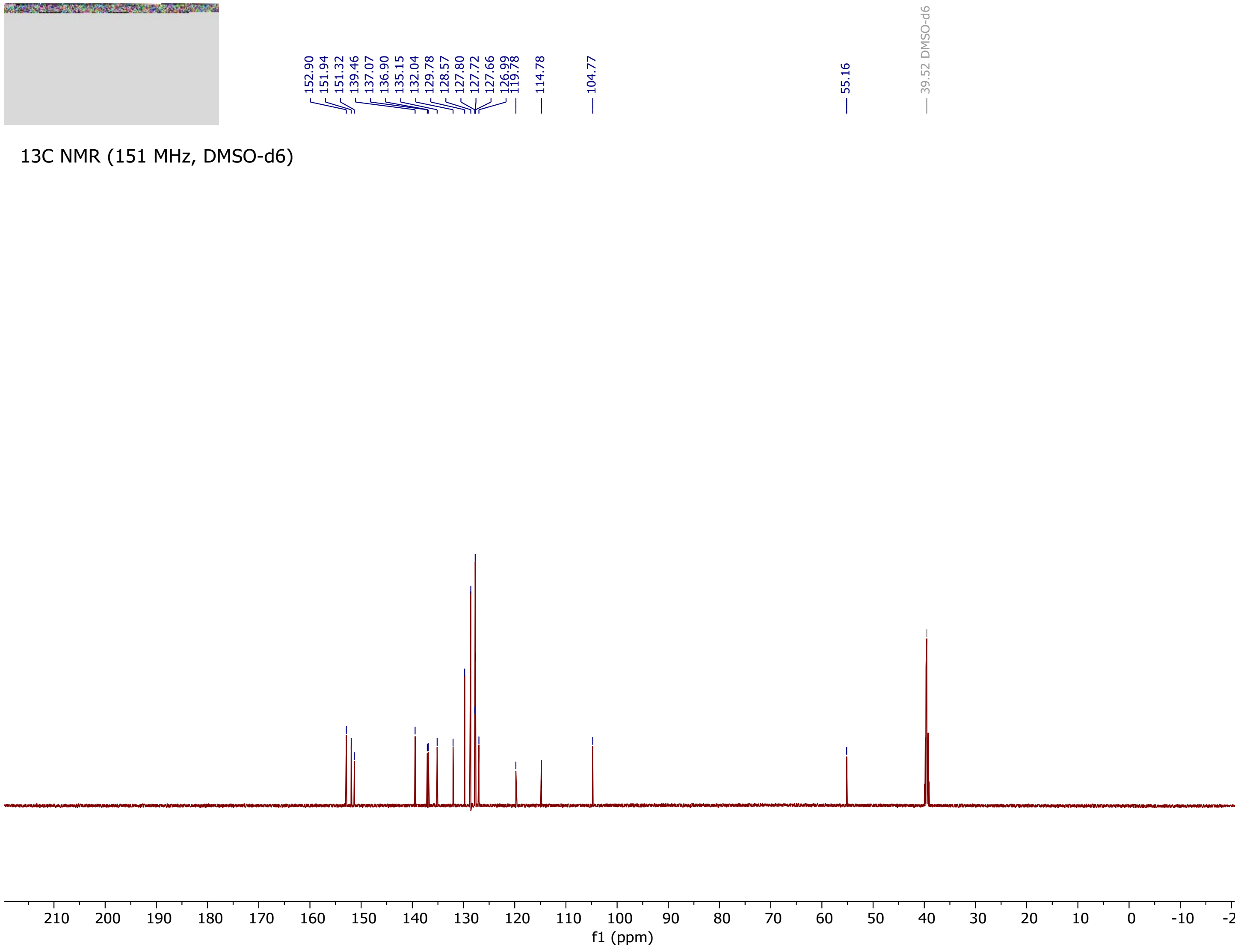
13C NMR (151 MHz, DMSO-d6)
152.90
151.94
151.32
139.46
137.07
136.90
135.15
132.04
129.78
128.57
127.80
127.72
127.66
126.99
119.78
114.78
104.77
55.16
39.52 DMSO-d6
210 200 190 180 170 160 150 140 130 120 110 100 90 80 70 60 50 40 30 20 10 0 -10
f1 (ppm)

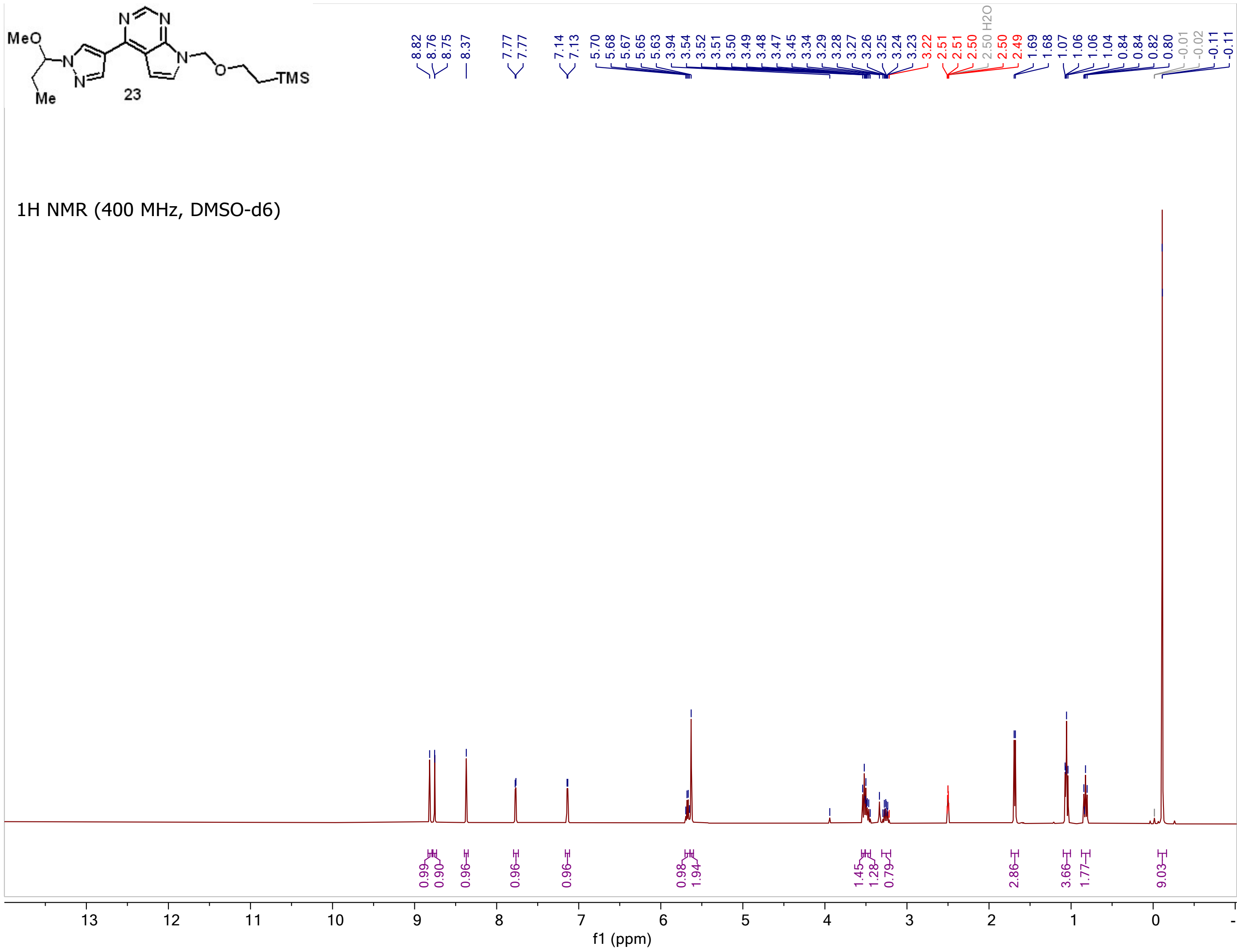

MeO
N
N
N
N
N
O
TMS
Me
23
1H NMR (400 MHz, DMSO-d6)
f1 (ppm)

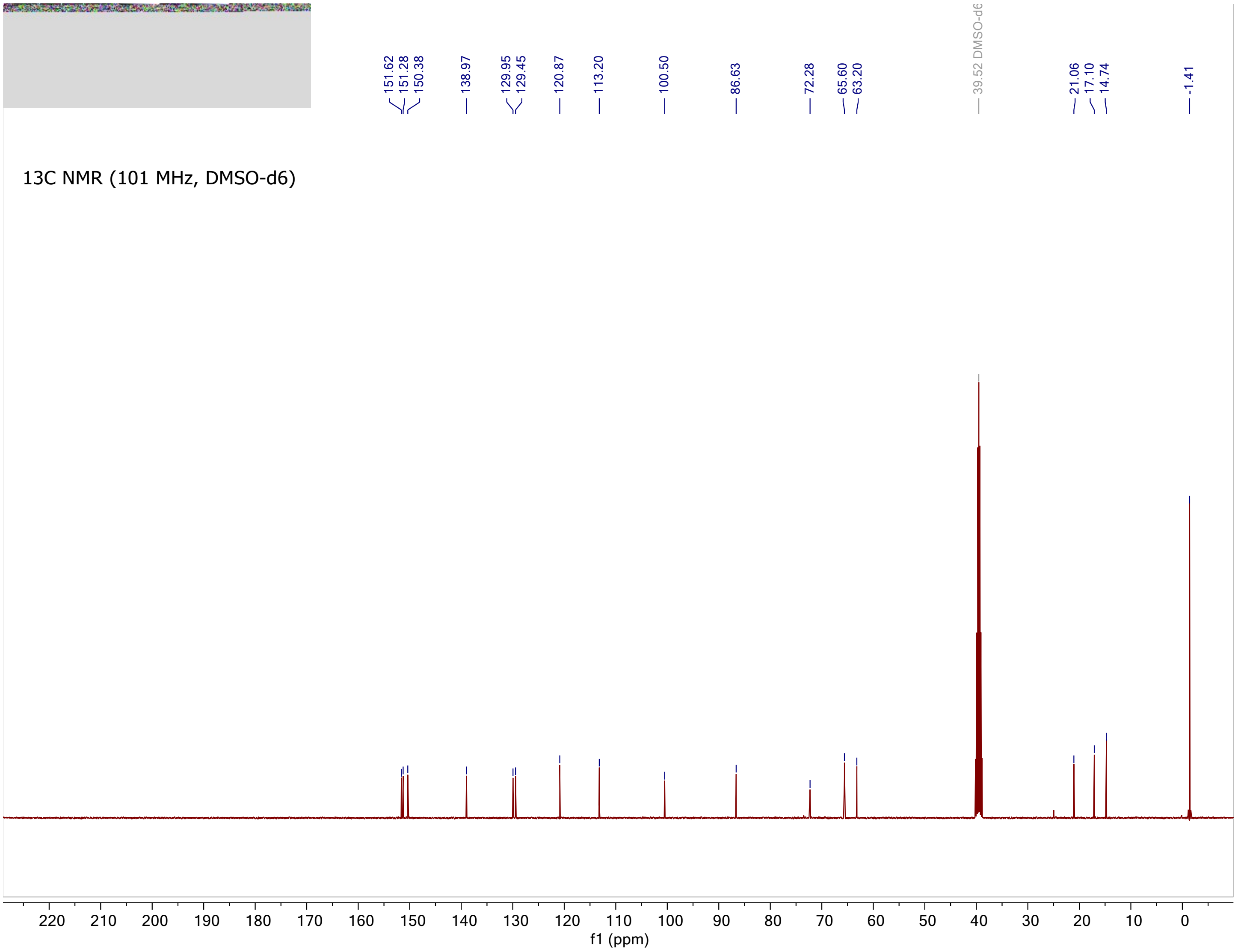
13C NMR (101 MHz, DMSO-d6)
151.62
151.28
150.38
138.97
129.95
129.45
120.87
113.20
100.50
86.63
72.28
65.60
63.20
39.52 DMSO-d6
21.06
17.10
14.74
-1.41
220 210 200 190 180 170 160 150 140 130 120 110 100 90 80 70 60 50 40 30 20 10 0
f1 (ppm)

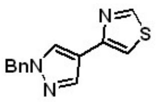

24

1H NMR (700 MHz, DMSO-d6)

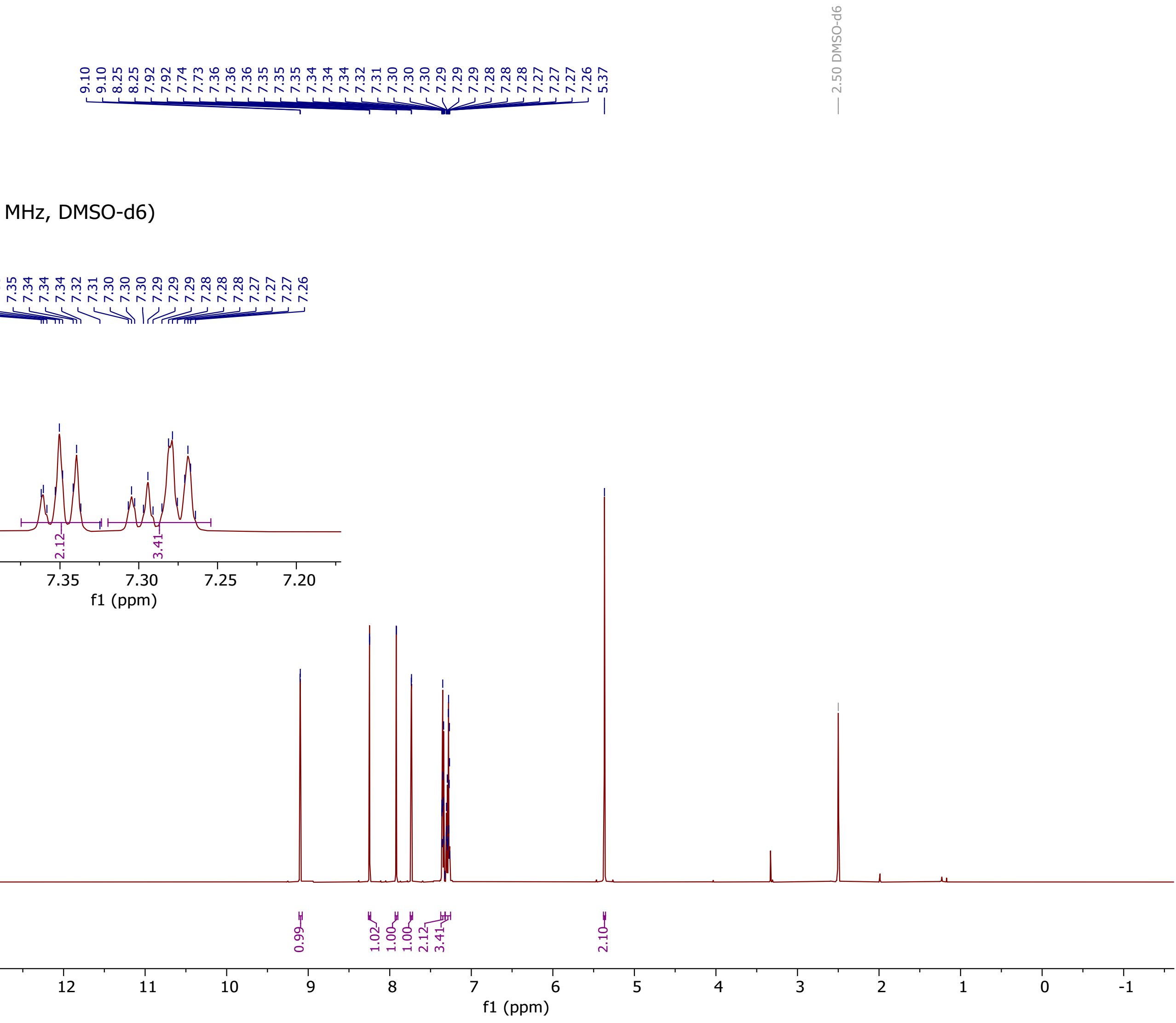

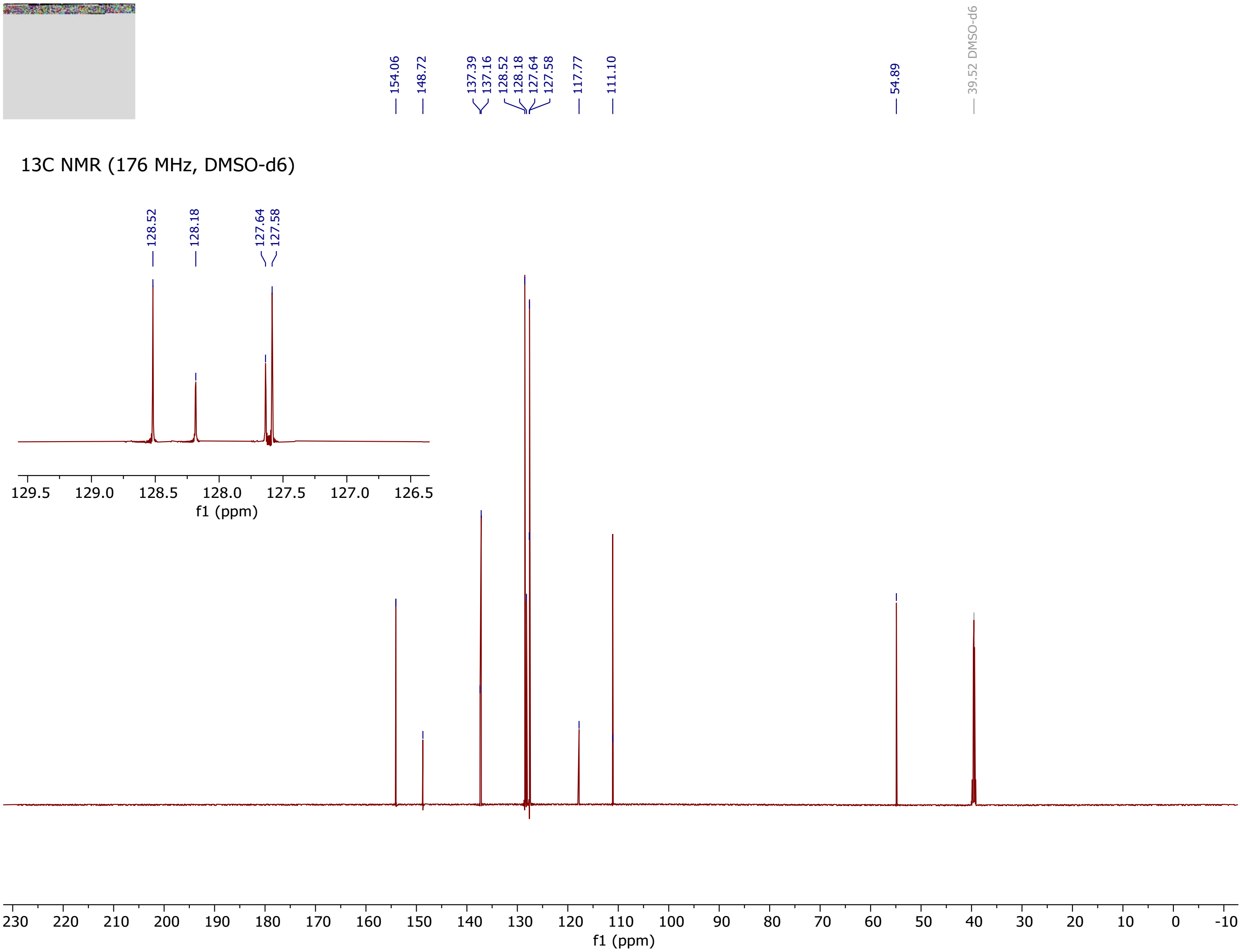
13C NMR (176 MHz, DMSO-d6)
154.06
148.72
137.39
137.16
128.52
128.18
127.64
127.58
117.77
111.10
54.89
39.52 DMSO-d6
f1 (ppm)
f1 (ppm)

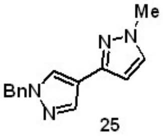

25

1H NMR (400 MHz, CDCl3)

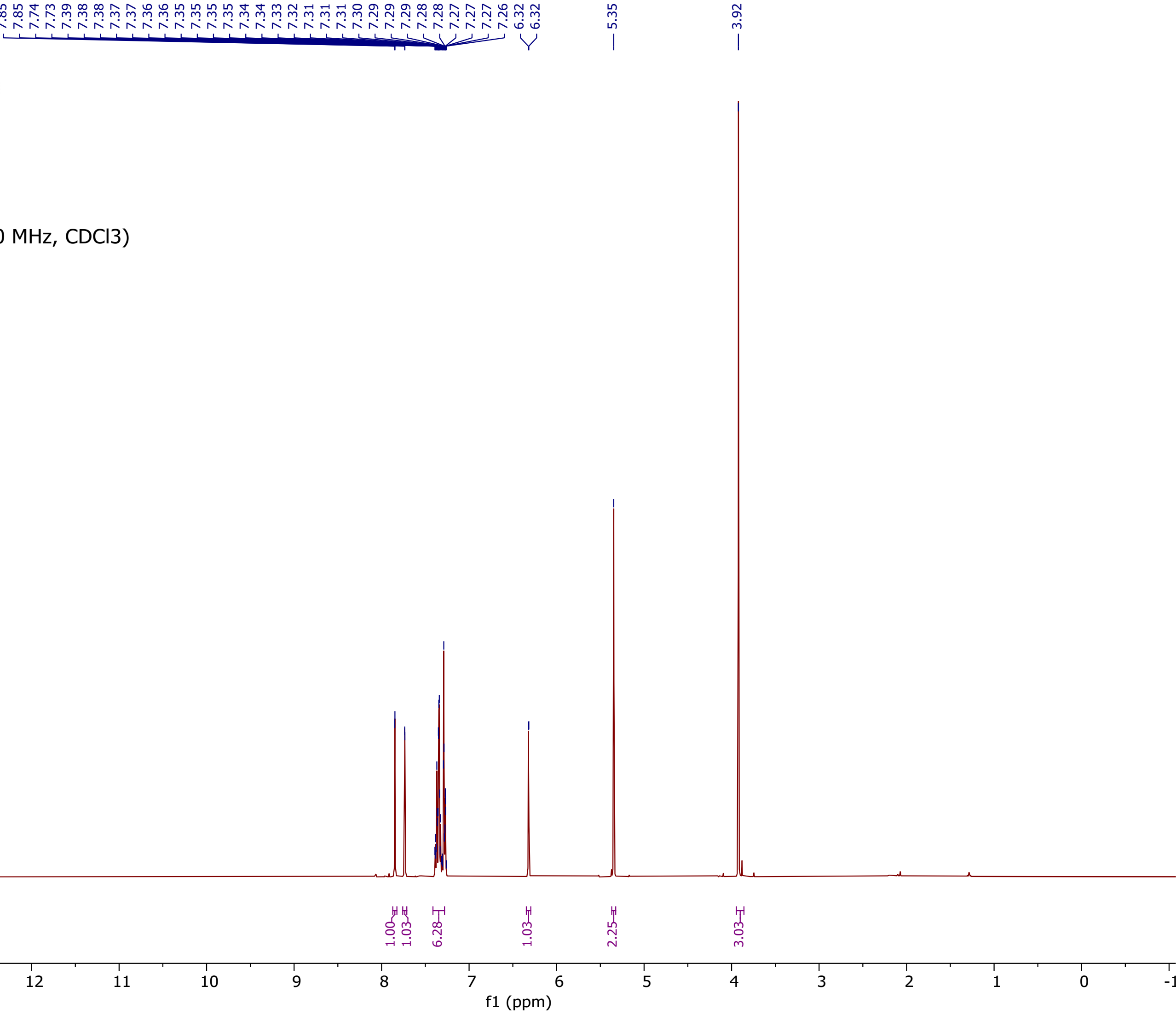

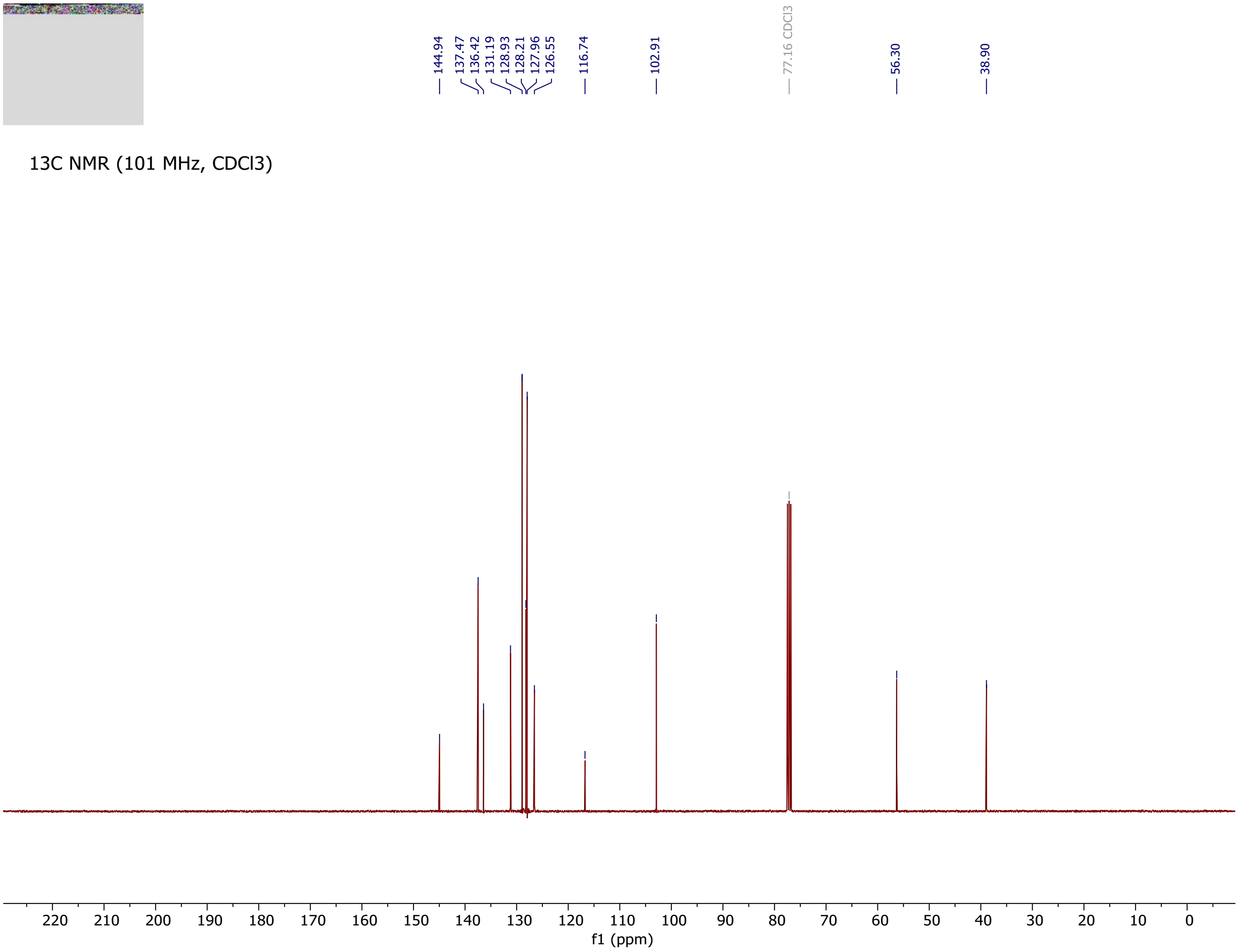
13C NMR (101 MHz, CDCl3)
144.94
137.47
136.42
131.19
128.93
128.21
127.96
126.55
116.74
102.91
77.16 CDCl3
56.30
38.90
220 210 200 190 180 170 160 150 140 130 120 110 100 90 80 70 60 50 40 30 20 10 0
f1 (ppm)

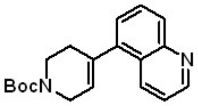

26

1H NMR (400 MHz, CDCl3)

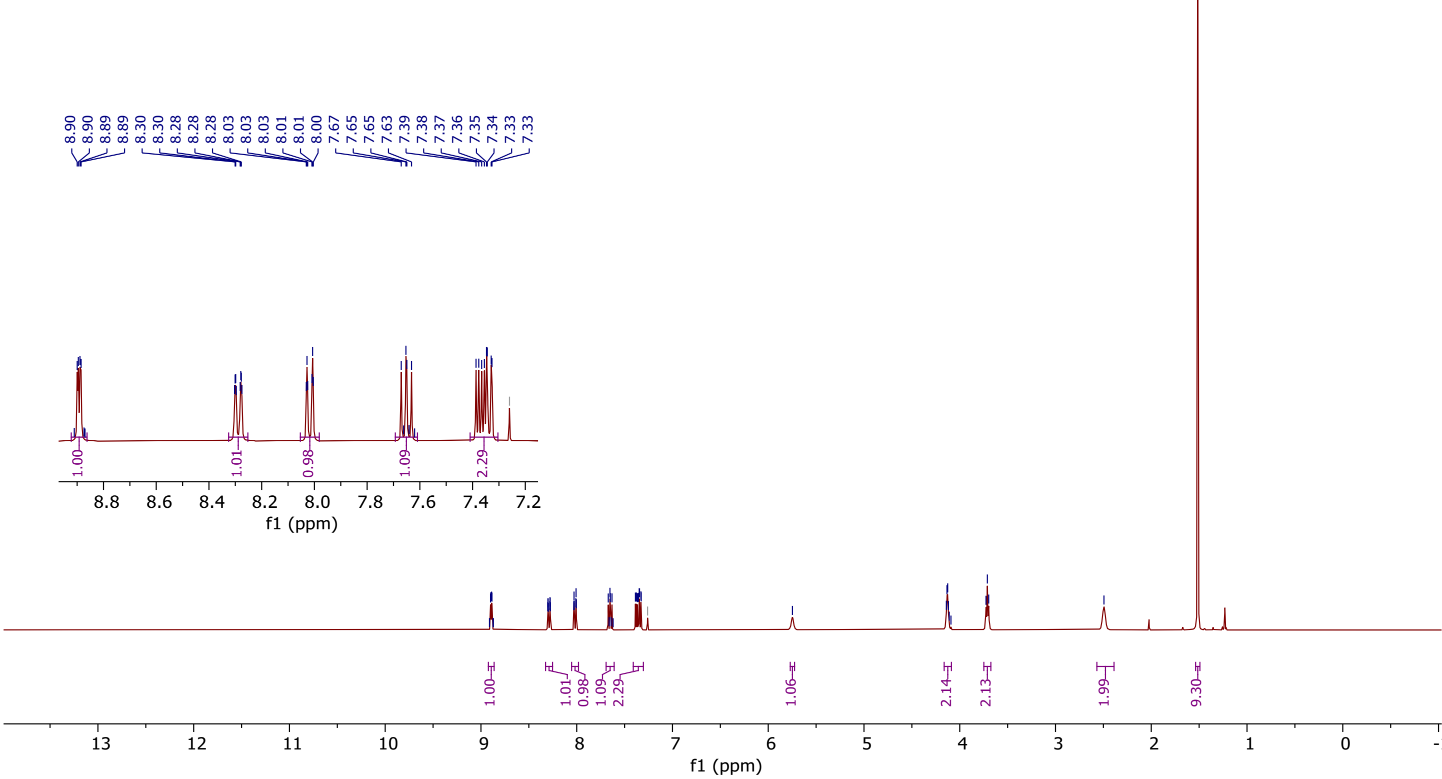

13C NMR (101 MHz, CDCl3)

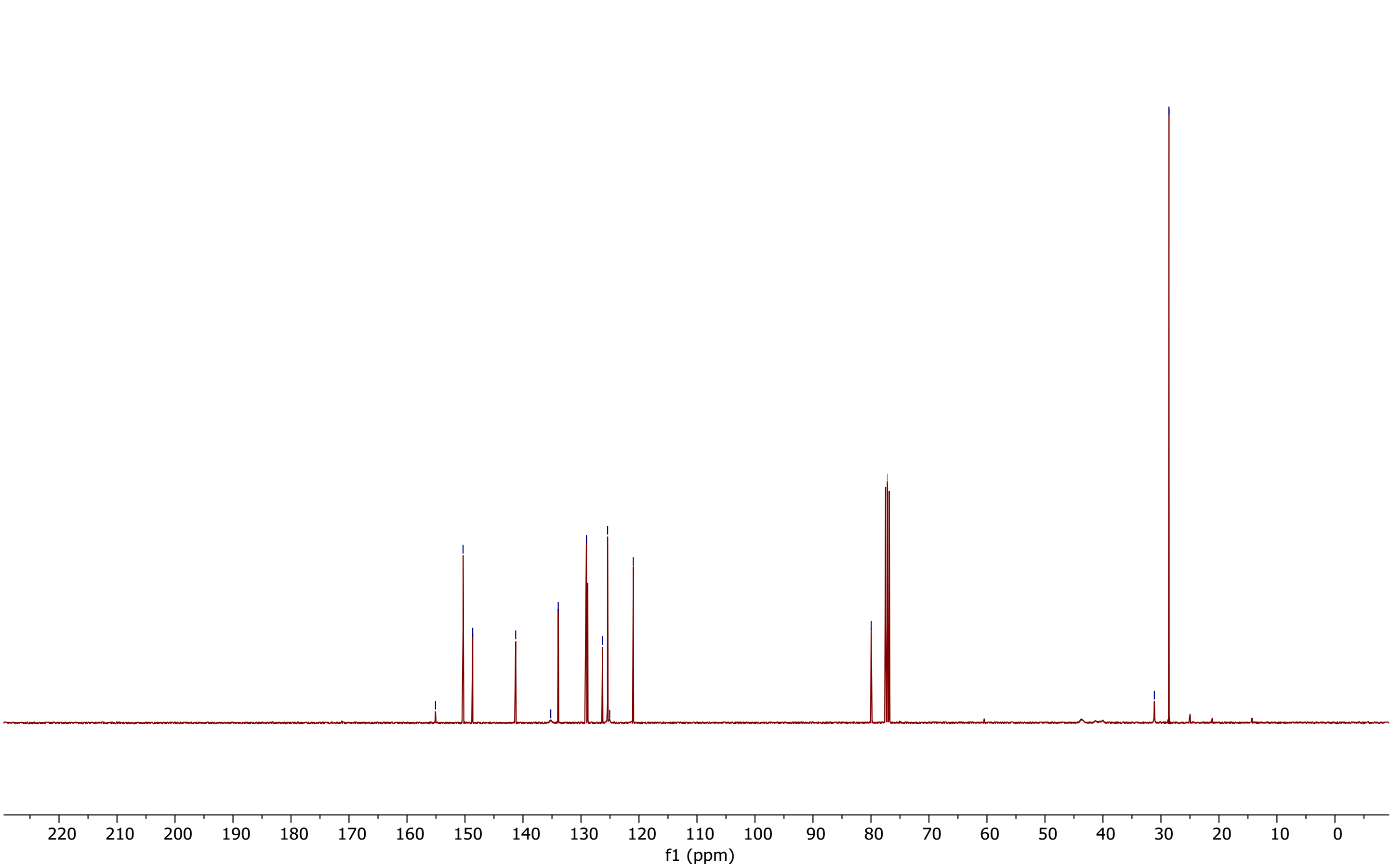

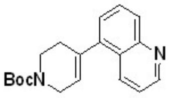

26

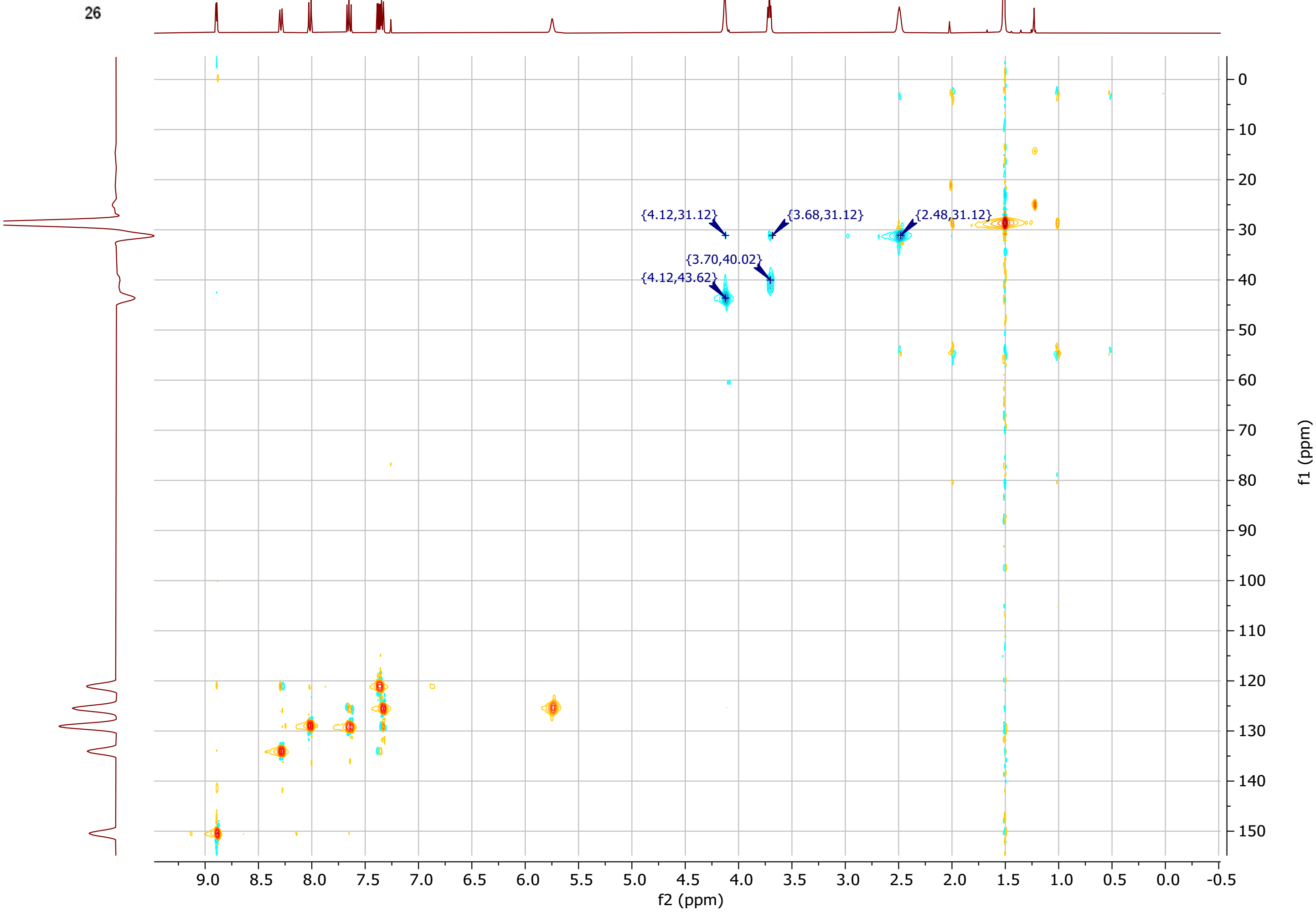
HSQC Spectrum
{4.12,31.12}
{3.68,31.12}
{2.48,31.12}
{3.70,40.02}
{4.12,43.62}
f2 (ppm)
f1 (ppm)

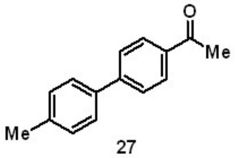

27

1H NMR (400 MHz, DMSO-d6)

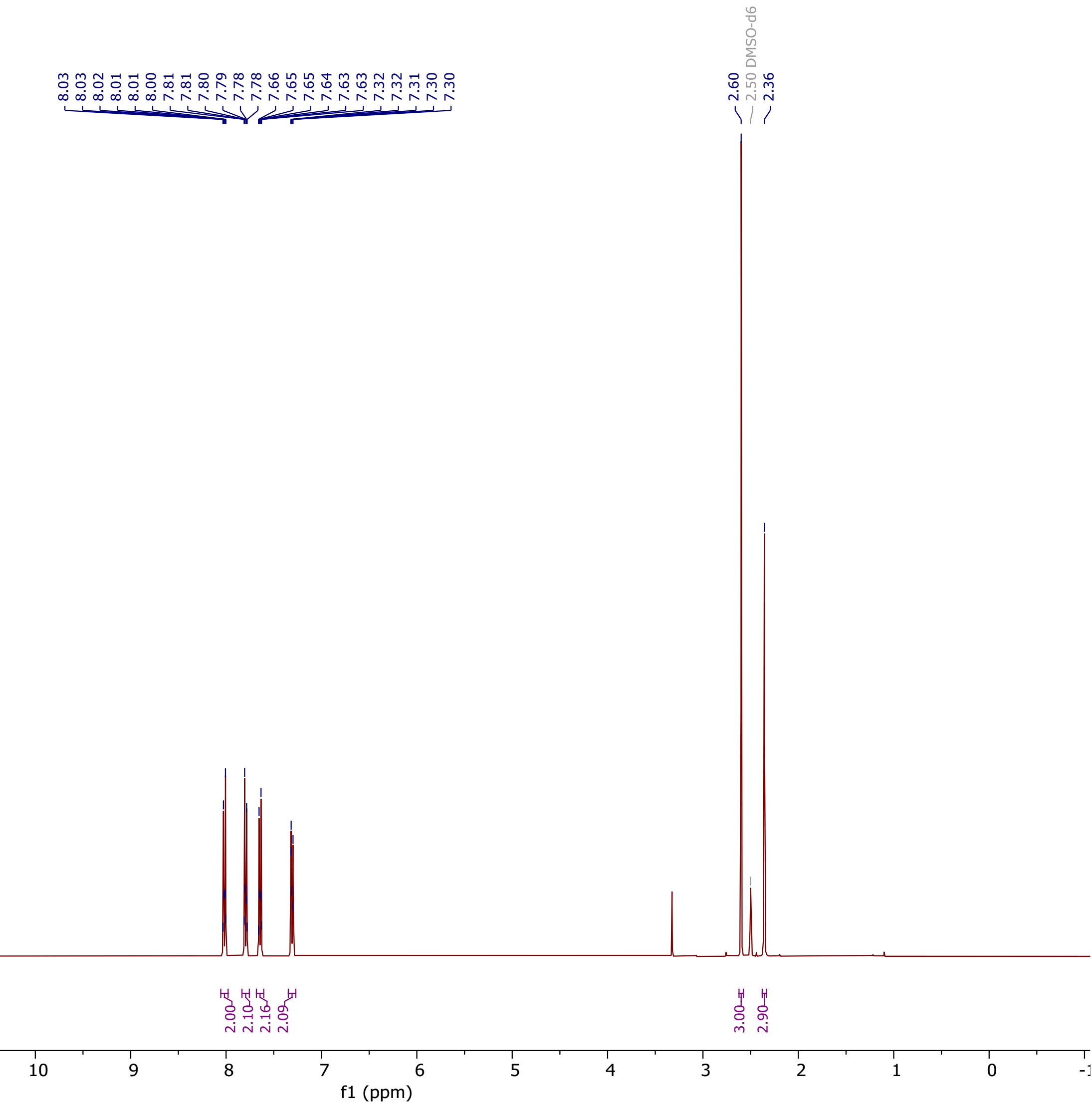

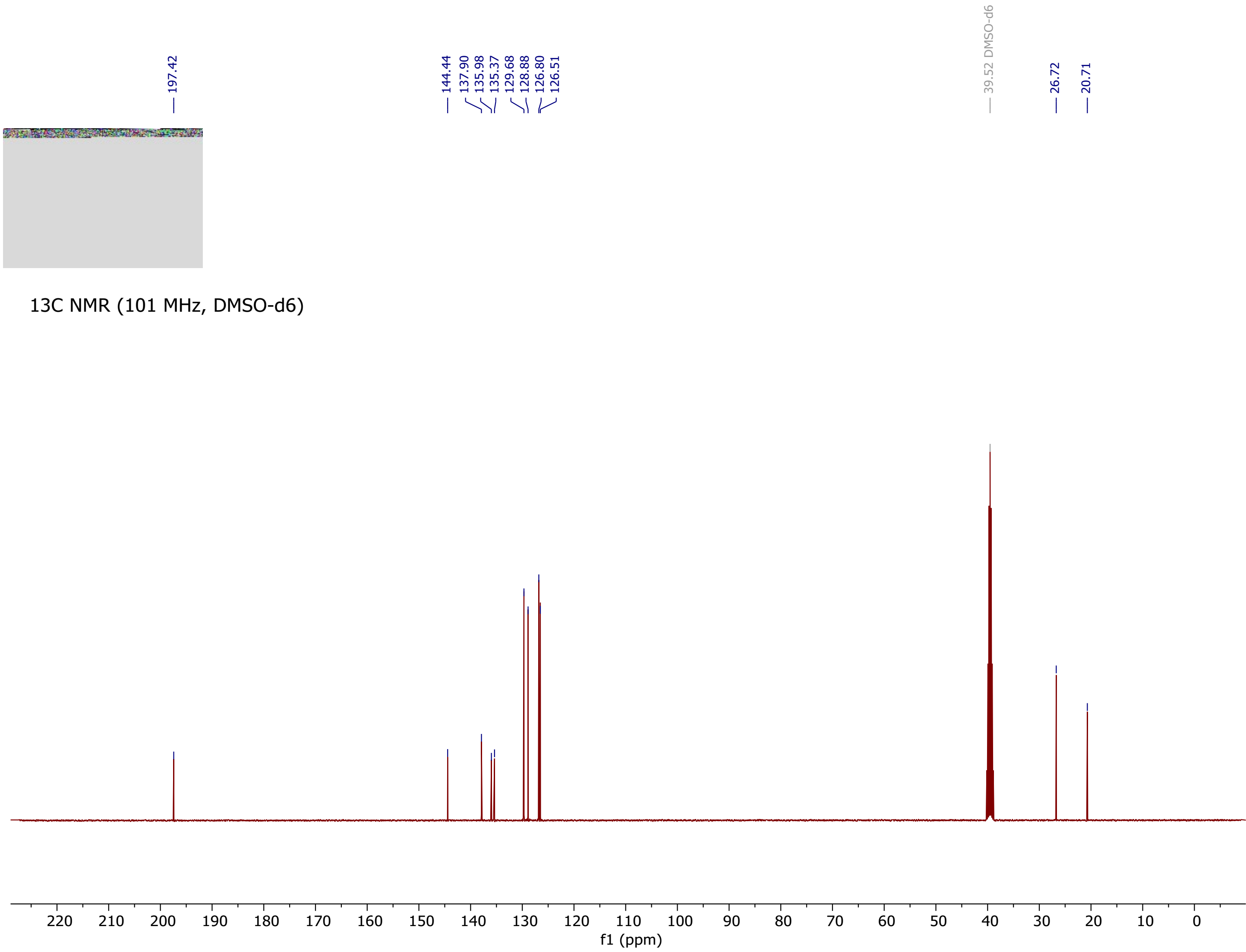
197.42
144.44
137.90
135.98
135.37
129.68
128.88
126.80
126.51
39.52 DMSO-d6
26.72
20.71
13C NMR (101 MHz, DMSO-d6)
220 210 200 190 180 170 160 150 140 130 120 110 100 90 80 70 60 50 40 30 20 10 0
f1 (ppm)

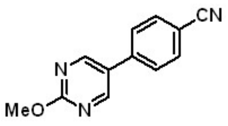

28

1H NMR (400 MHz, CDCl3)

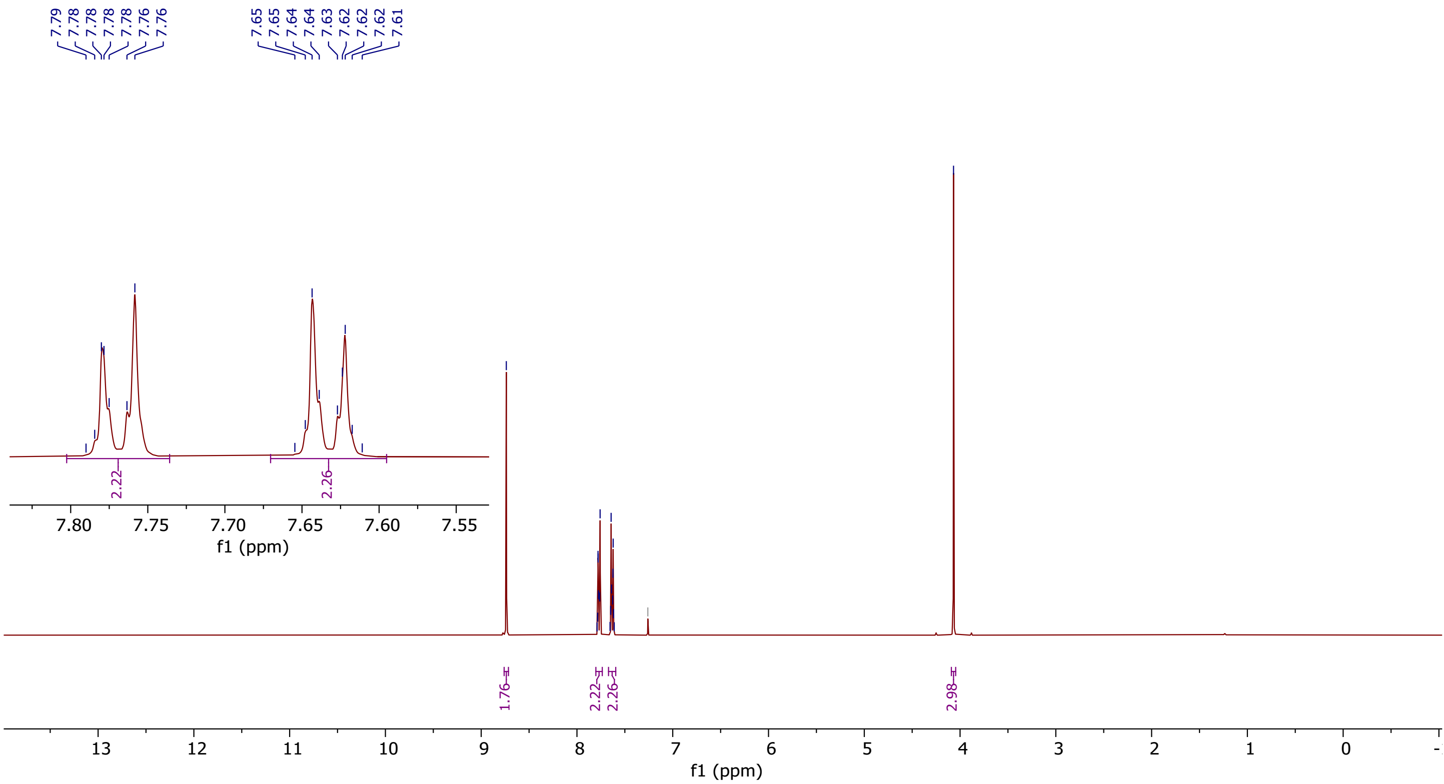

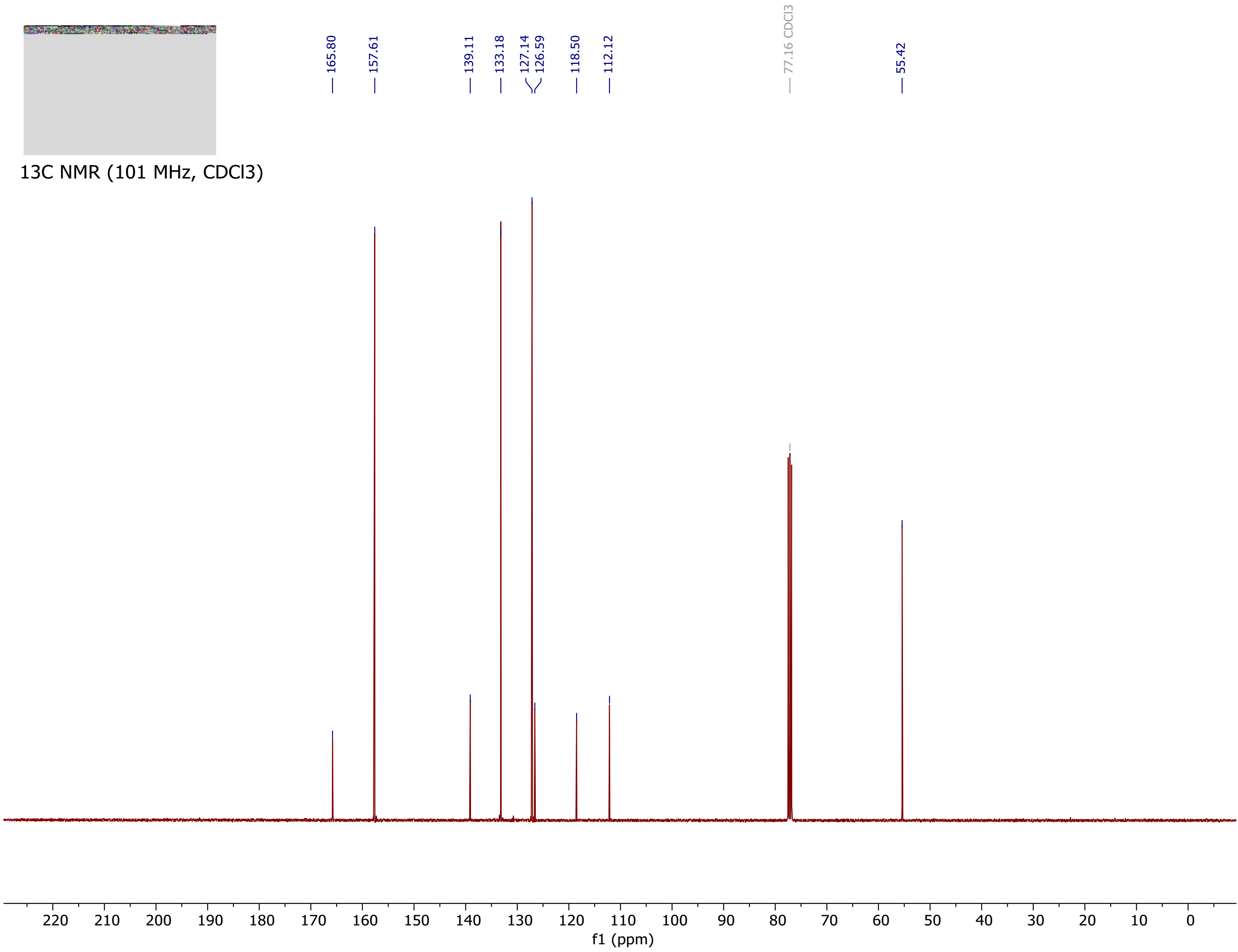
13C NMR (101 MHz, CDCl3)
165.80
157.61
139.11
133.18
127.14
126.59
118.50
112.12
77.16 CDCl3
55.42
220 210 200 190 180 170 160 150 140 130 120 110 100 90 80 70 60 50 40 30 20 10 0
f1 (ppm)

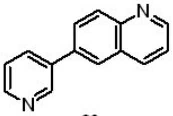

29

1H NMR (400 MHz, DMSO-d6)

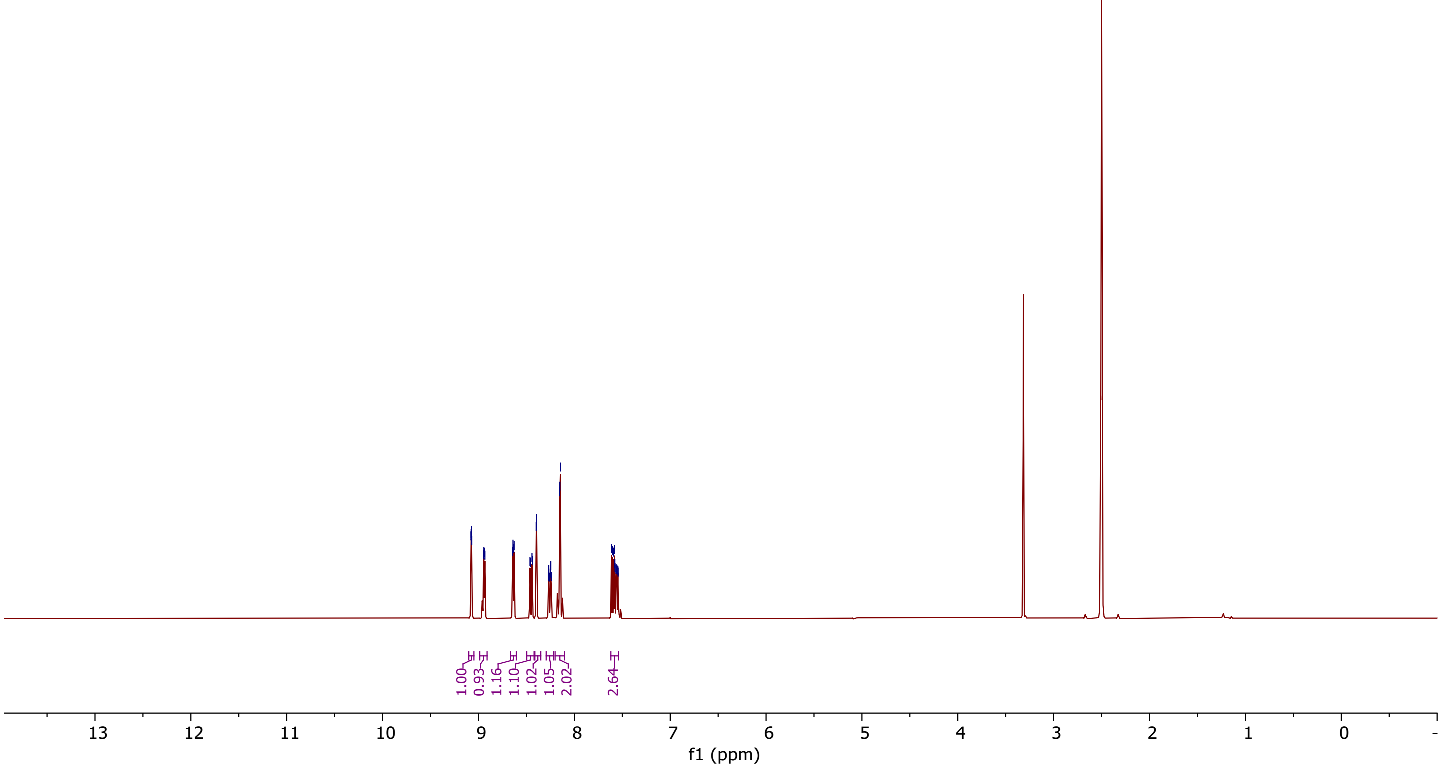

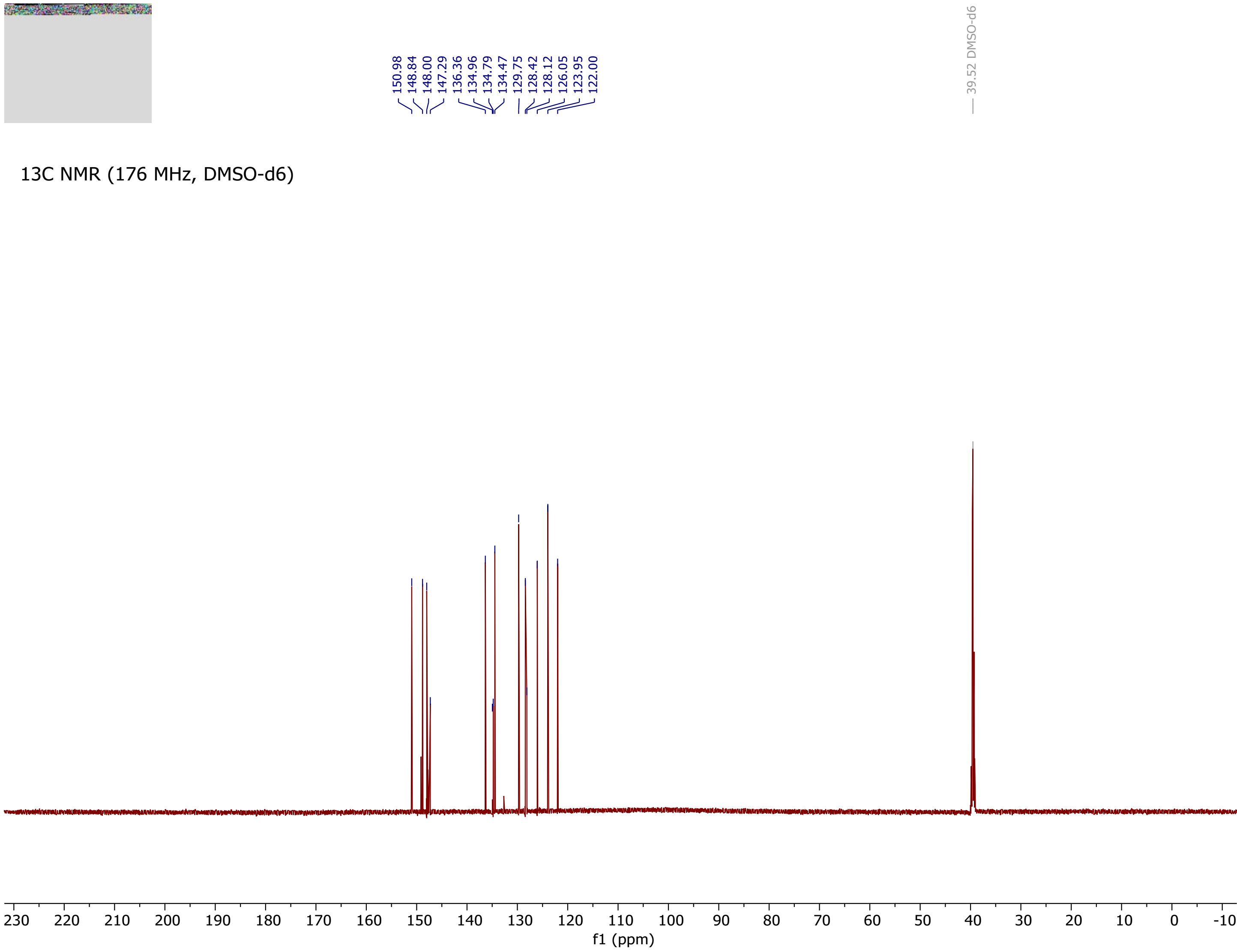
150.98
148.84
148.00
147.29
136.36
134.96
134.79
134.47
129.75
128.42
128.12
126.05
123.95
122.00
39.52 DMSO-d6
13C NMR (176 MHz, DMSO-d6)
230 220 210 200 190 180 170 160 150 140 130 120 110 100 90 80 70 60 50 40 30 20 10 0 -10
f1 (ppm)

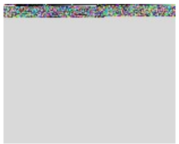

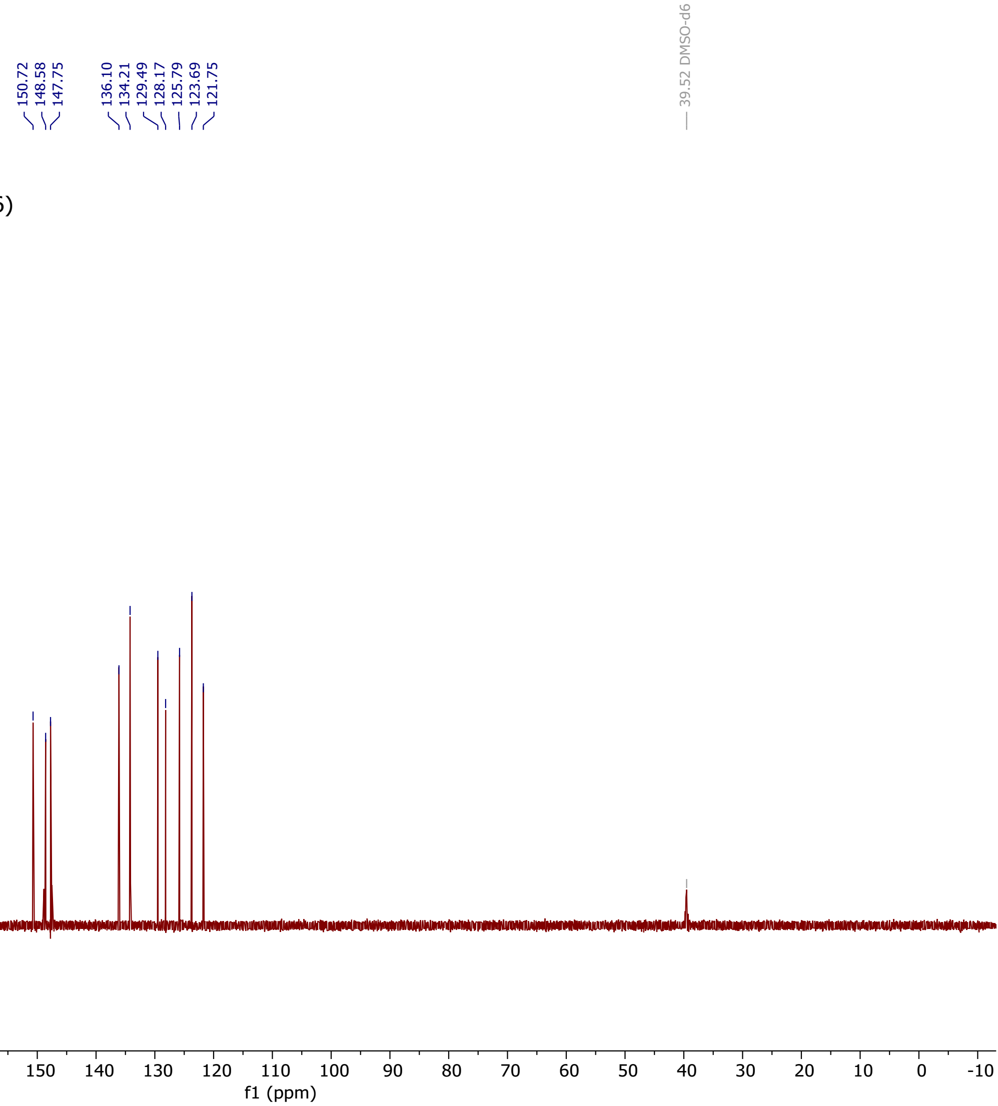

13C NMR, DEPT-135 (176 MHz, DMSO-d6)

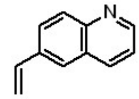

30

1H NMR (400 MHz, CDCl3)

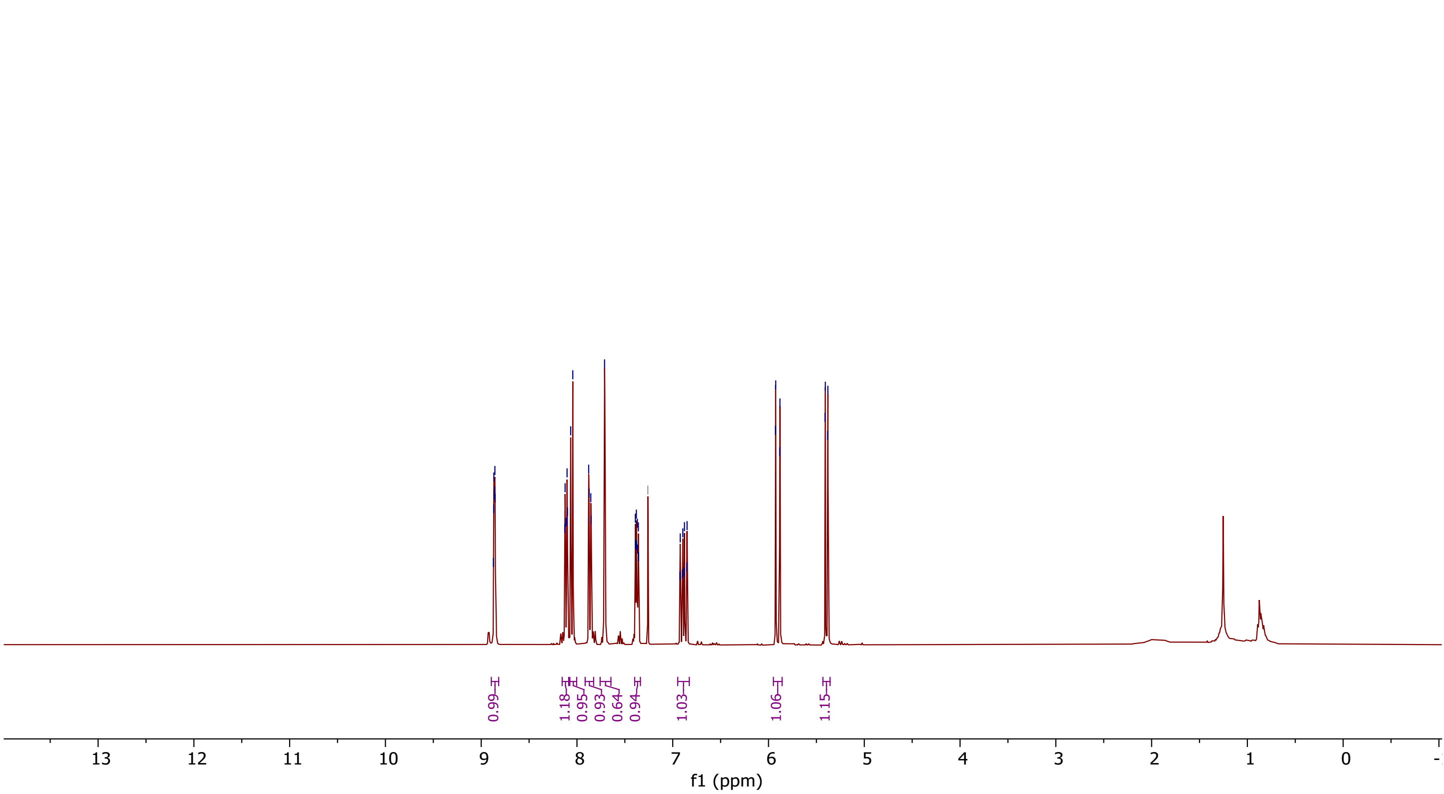

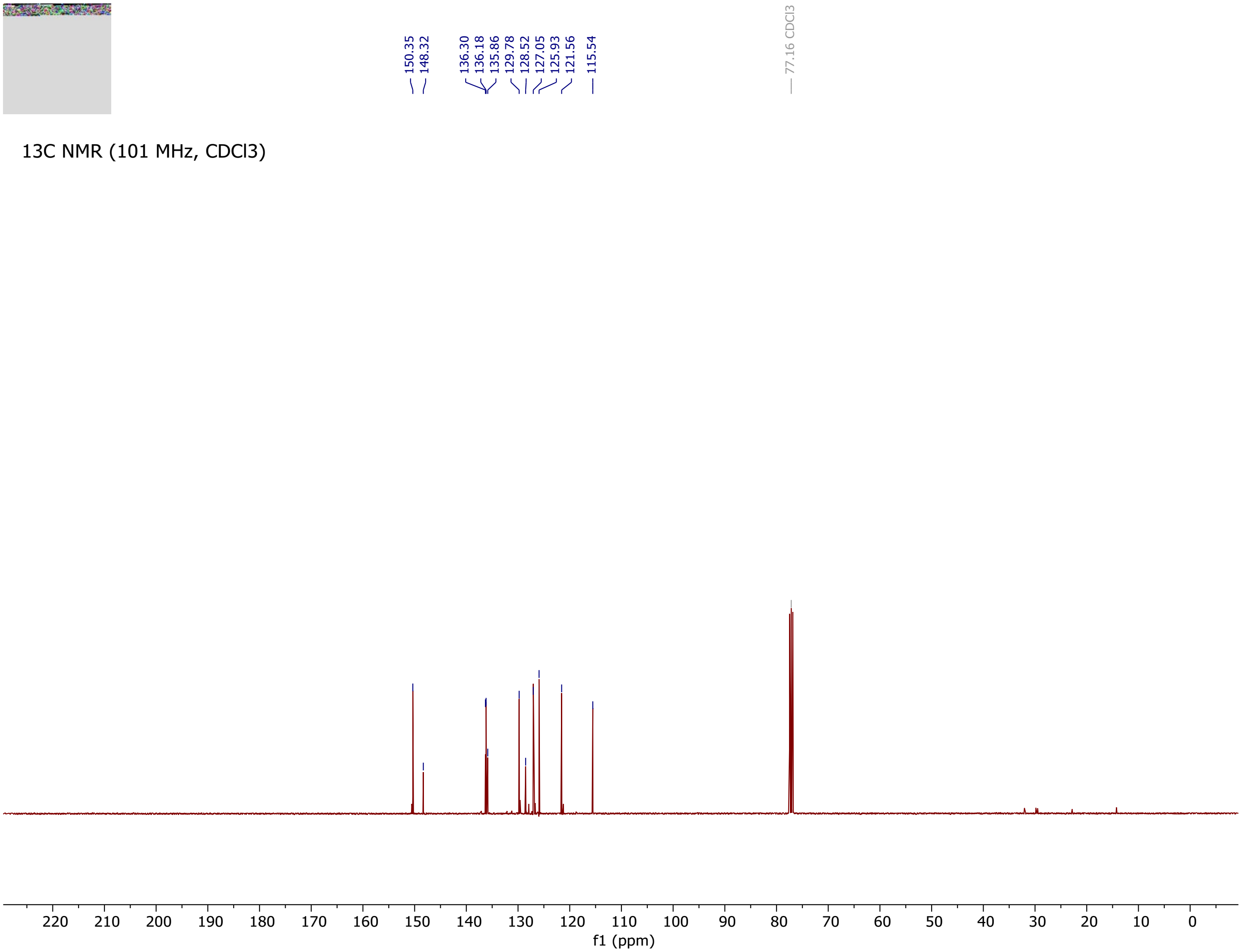
150.35
148.32
136.30
136.18
135.86
129.78
128.52
127.05
125.93
121.56
115.54
77.16 CDCl3
13C NMR (101 MHz, CDCl3)
220 210 200 190 180 170 160 150 140 130 120 110 100 90 80 70 60 50 40 30 20 10 0
f1 (ppm)

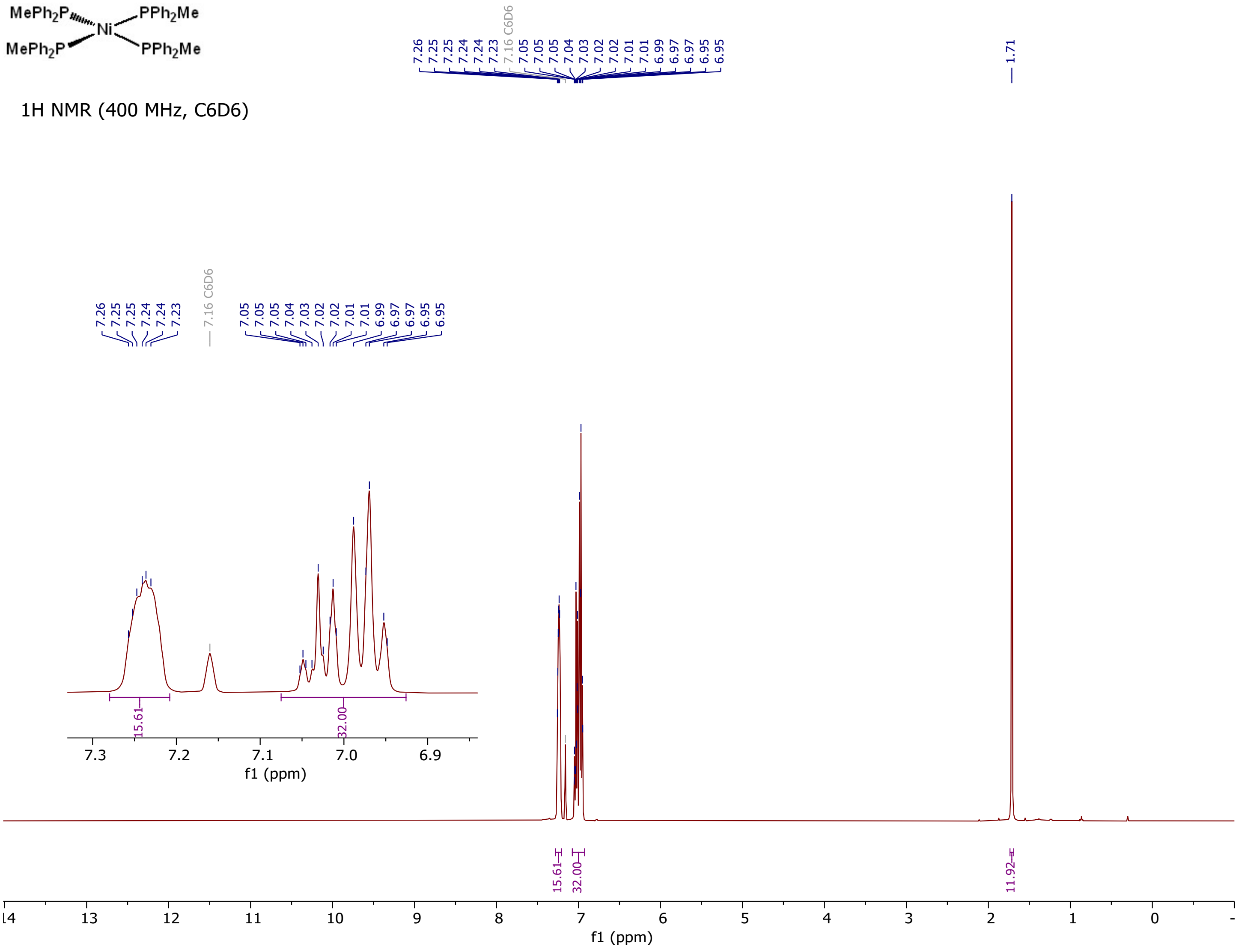
MePh2P
PPh2Me
Ni
MePh2P
PPh2Me
1H NMR (400 MHz, C6D6)
7.26 7.25 7.25 7.24 7.24 7.23 7.16 C6D6 7.05 7.05 7.05 7.04 7.03 7.02 7.02 7.01 7.01 6.99 6.97 6.95 6.95
1.71
15.61
32.00
11.92
f1 (ppm)

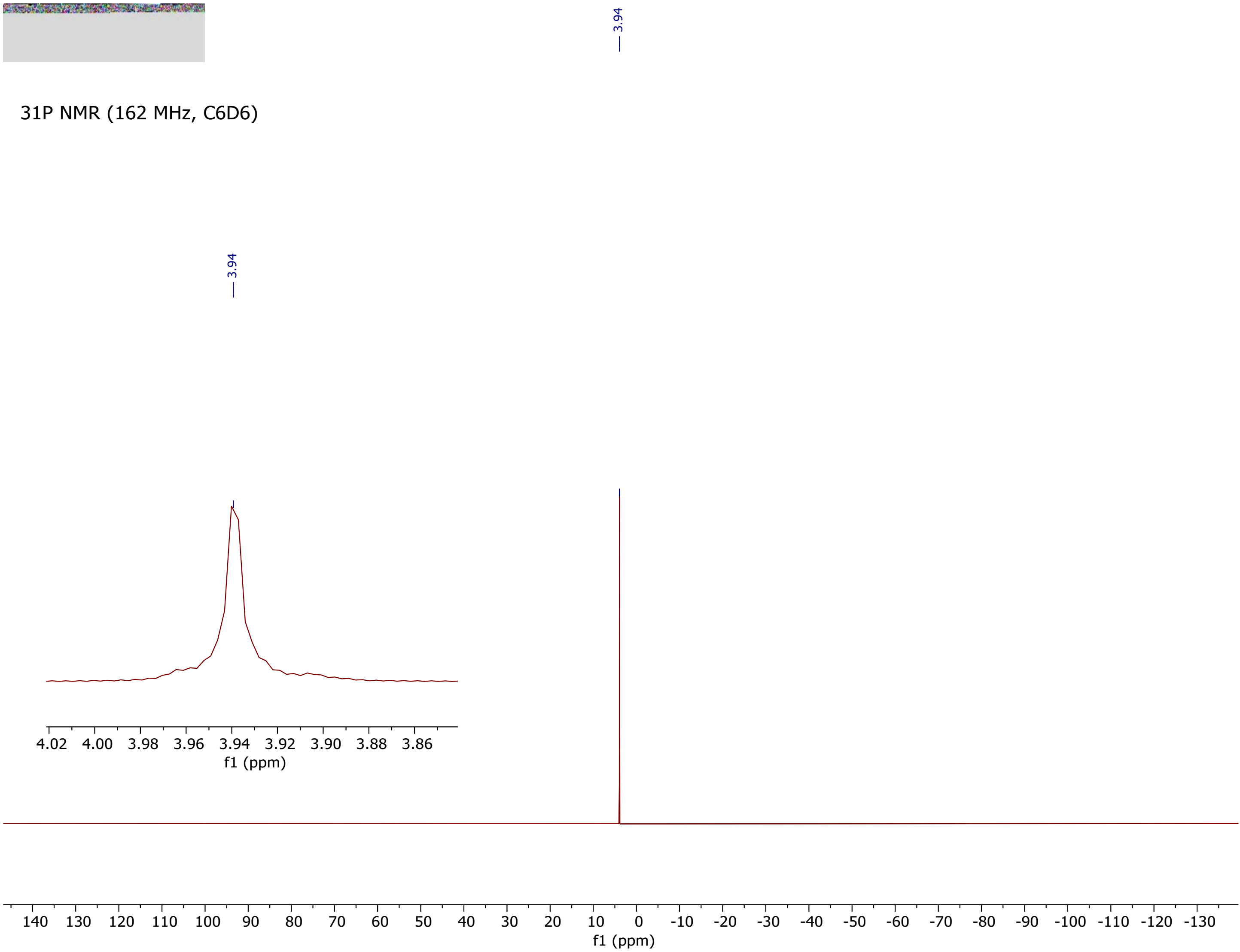
31P NMR (162 MHz, C6D6)
3.94
3.94
4.02 4.00 3.98 3.96 3.94 3.92 3.90 3.88 3.86
f1 (ppm)
140 130 120 110 100 90 80 70 60 50 40 30 20 10 0 -10 -20 -30 -40 -50 -60 -70 -80 -90 -100 -110 -120 -130
f1 (ppm)

PPh_2Me-HBF_4

1H NMR (400 MHz, CDCl3)

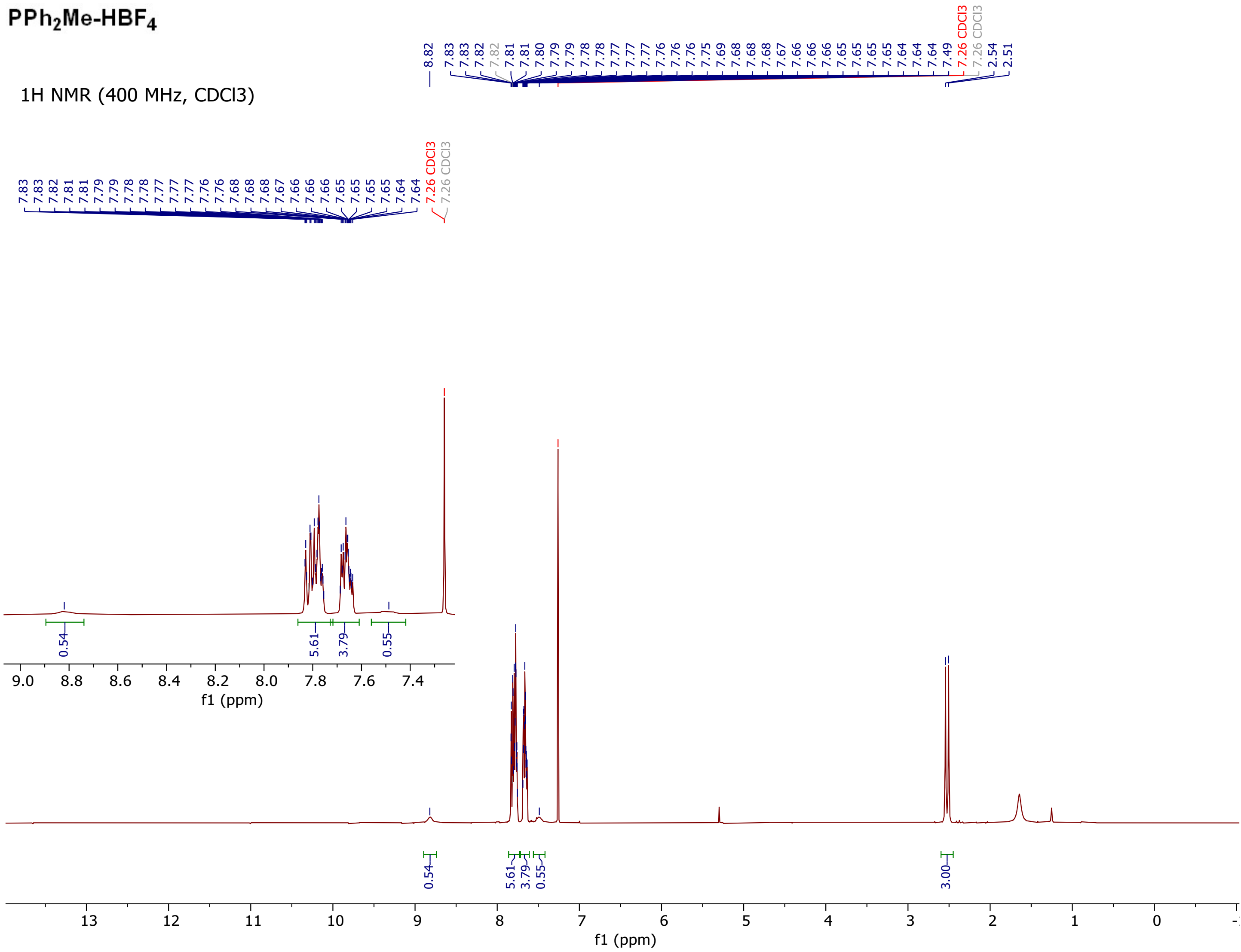

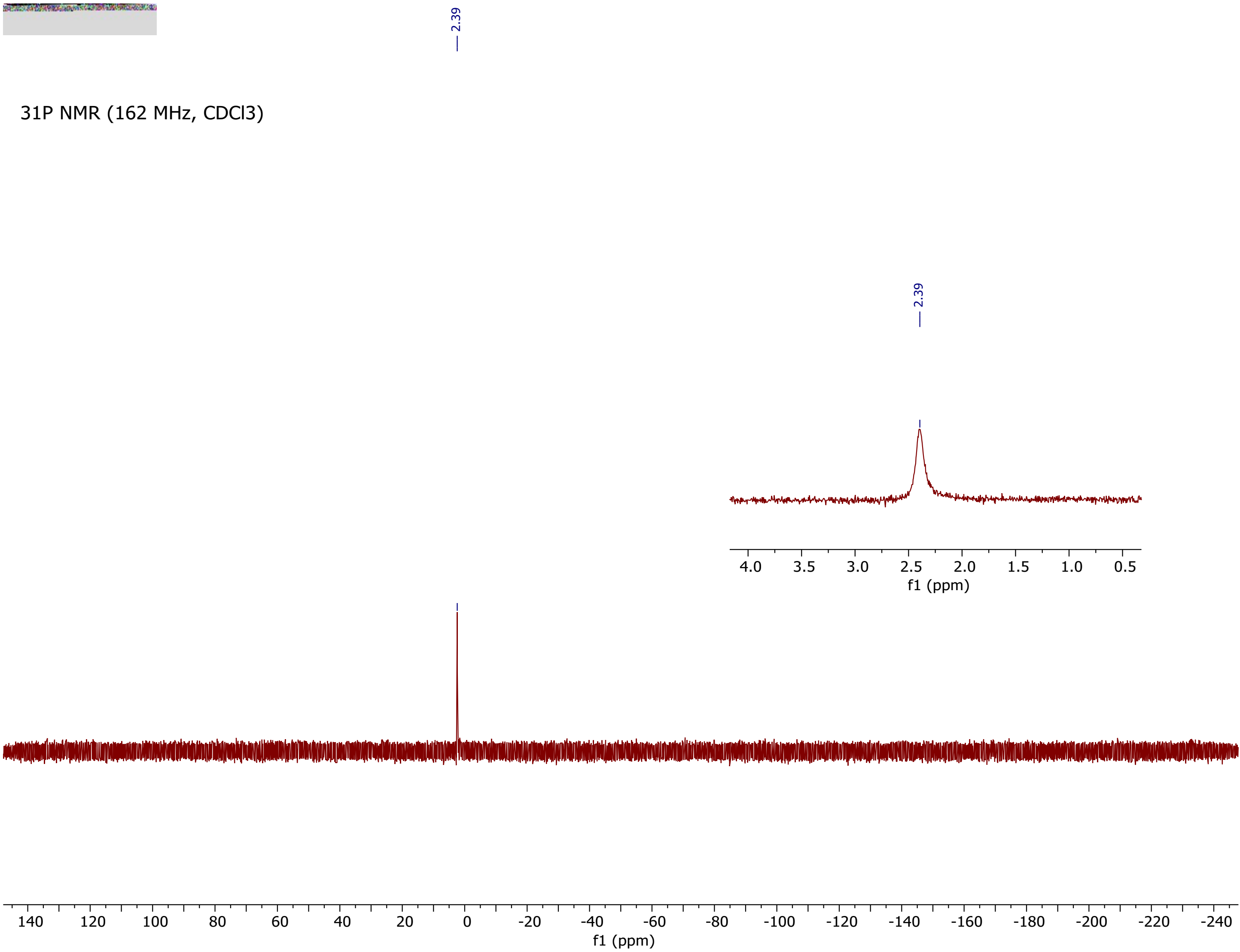
31P NMR (162 MHz, CDCl3)
2.39
2.39
4.0 3.5 3.0 2.5 2.0 1.5 1.0 0.5
f1 (ppm)
140 120 100 80 60 40 20 0 -20 -40 -60 -80 -100 -120 -140 -160 -180 -200 -220 -240
f1 (ppm)

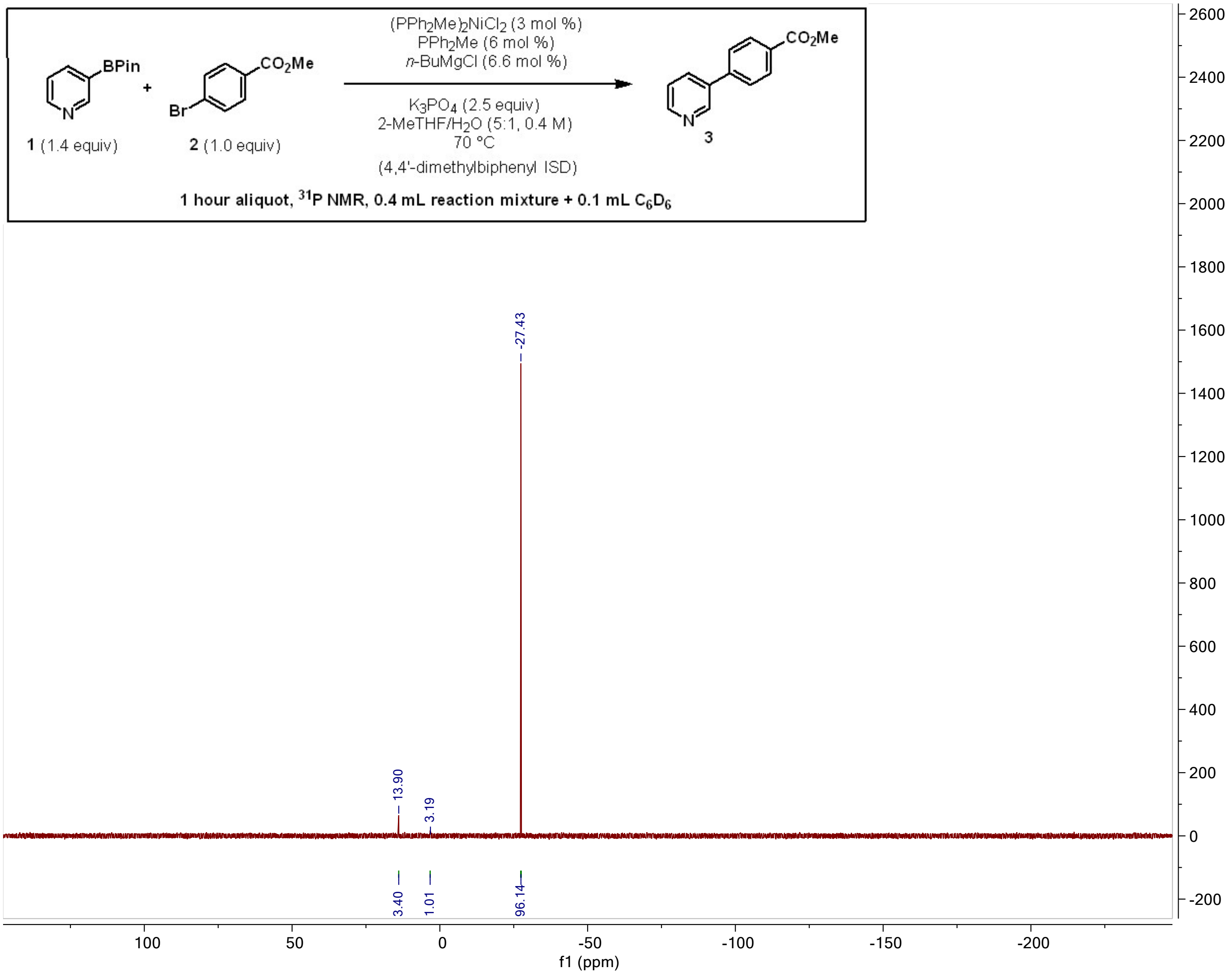

(PPh2Me)2NiCl2 (3 mol %)
PPh2Me (6 mol %)
n-BuMgCl (6.6 mol %)
K3PO4 (2.5 equiv)
2-MeTHF/H2O (5:1, 0.4 M)
70 °C
(4,4'-dimethylbiphenyl ISD)
BPin
CO2Me
Br
1 (1.4 equiv)
2 (1.0 equiv)
3
1 hour aliquot, 31P NMR, 0.4 mL reaction mixture + 0.1 mL C6D6
13.90
3.19
-27.43
3.40
1.01
96.14
f1 (ppm)

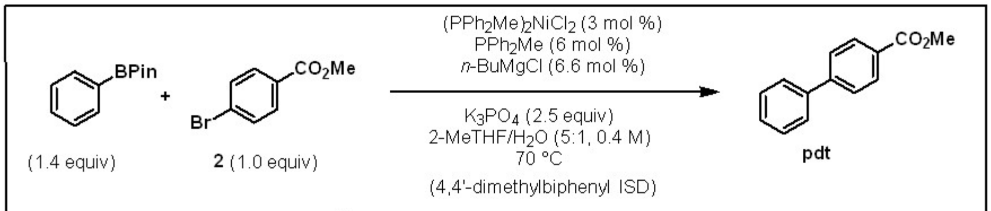

1 hour aliquot, ^{31}P NMR, 0.4 mL reaction mixture + 0.1 mL C_6D_6

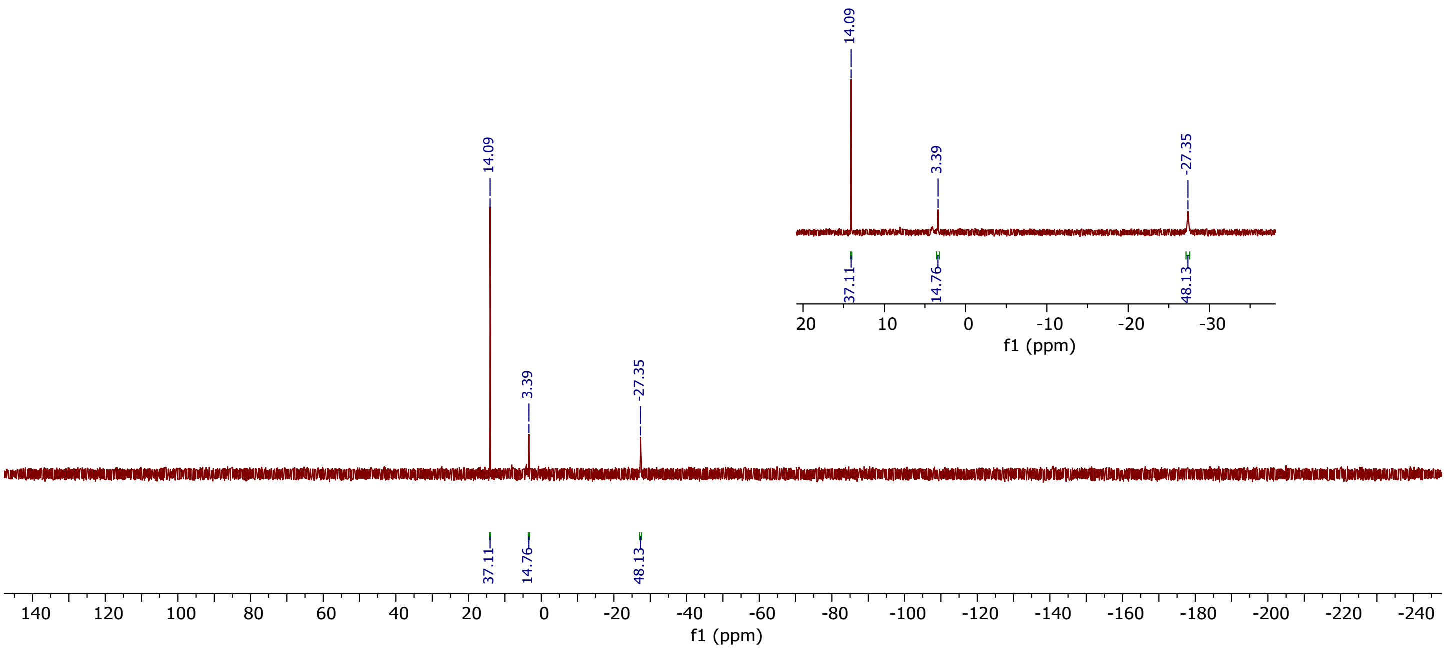

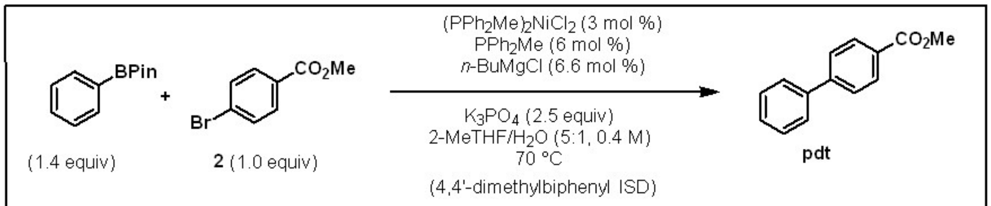

16 hour aliquot, ^{31}P NMR, 0.4 mL reaction mixture + 0.1 mL C_6D_6

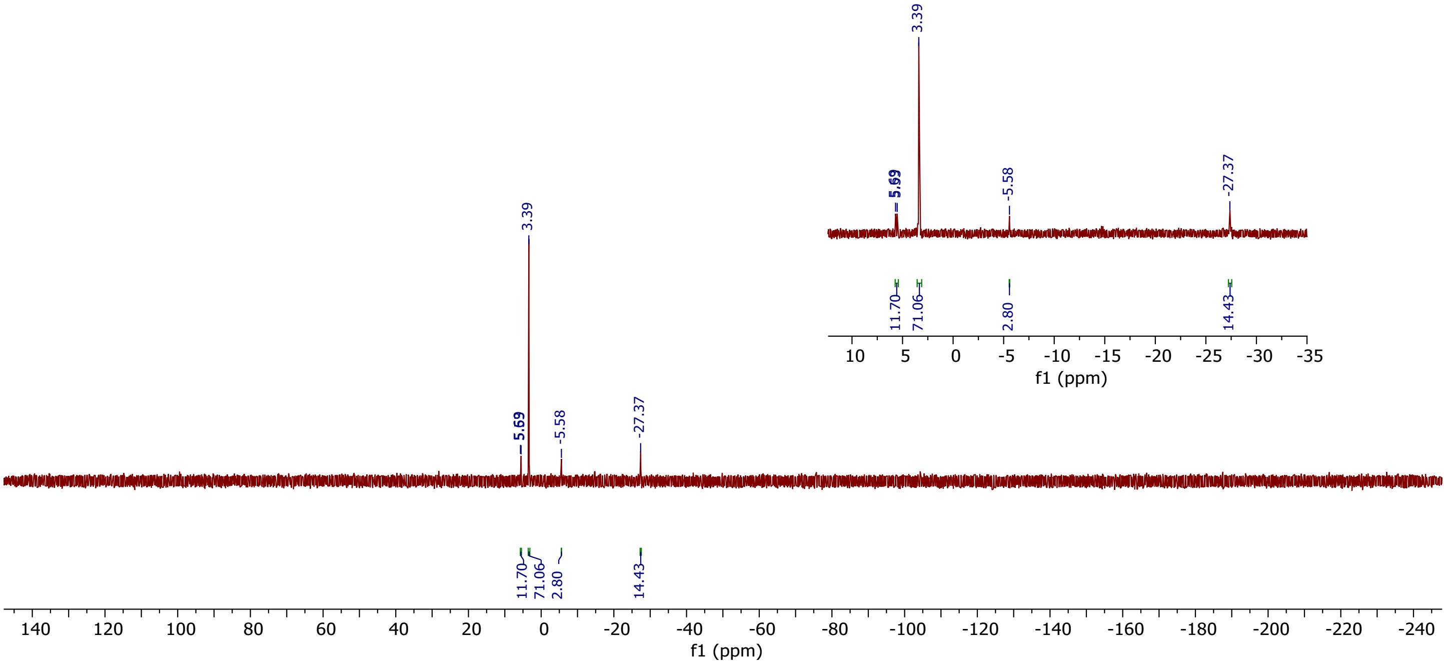

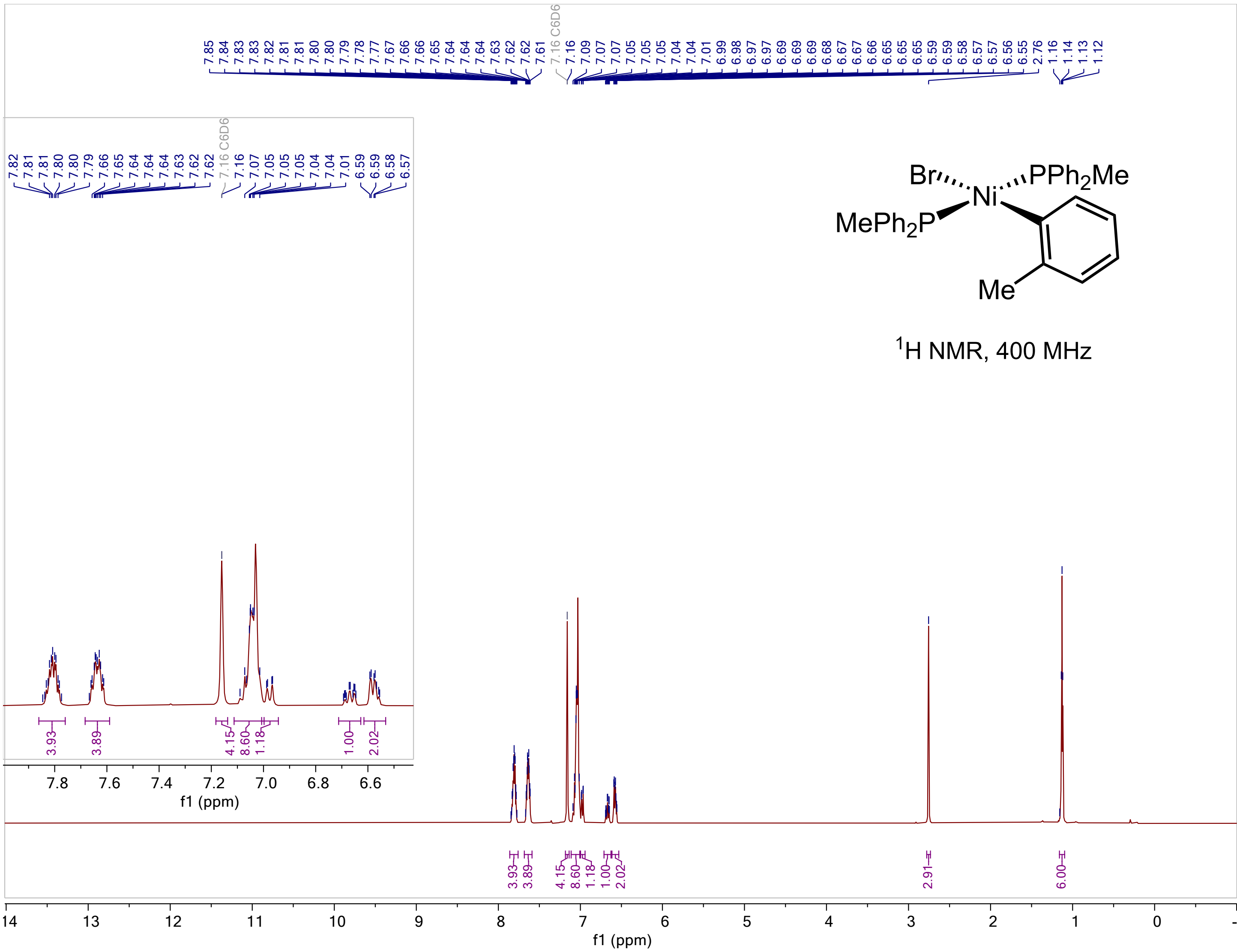
Br
Ni
PPh2Me
MePh2P
Me
1H NMR, 400 MHz
f1 (ppm)

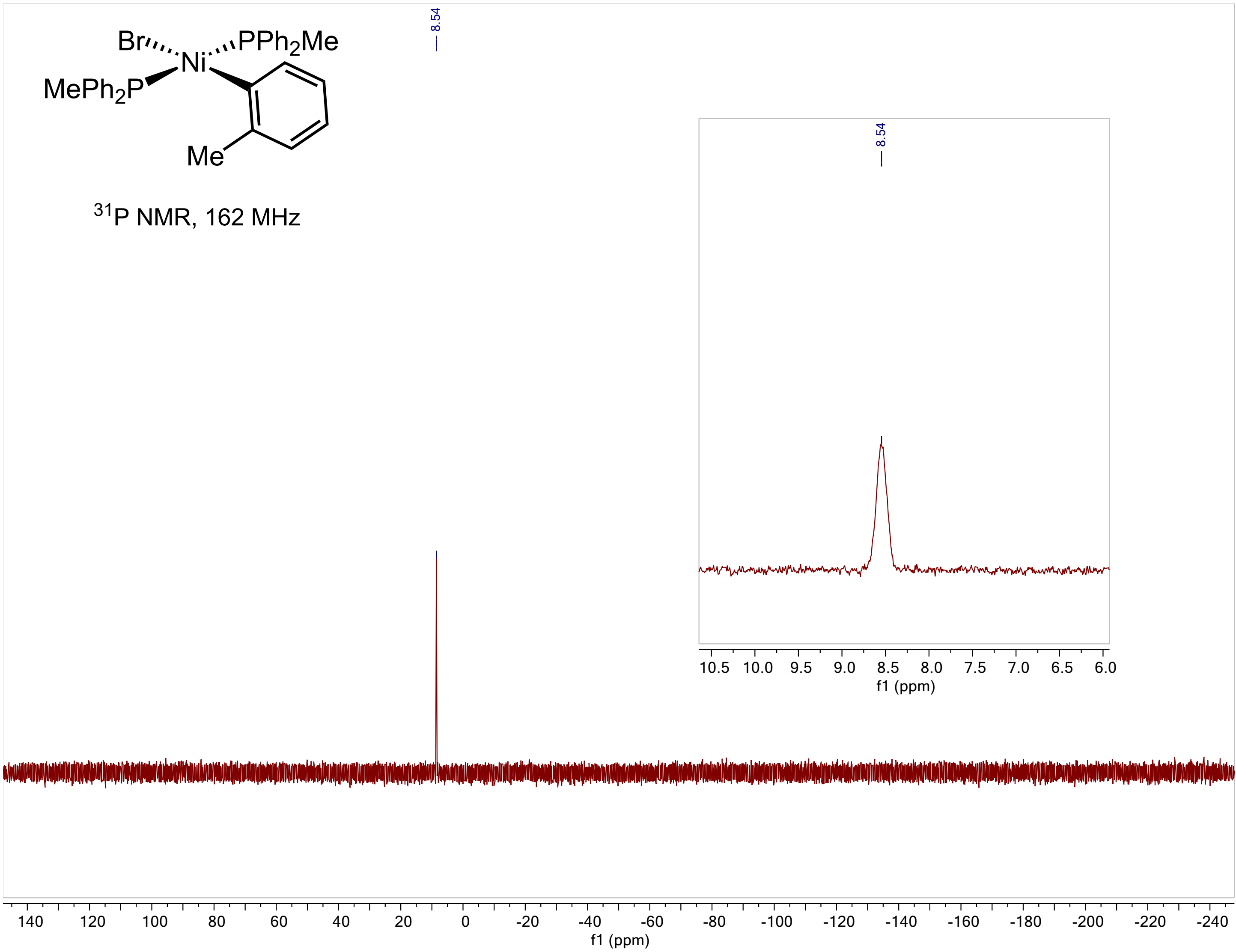
Br
Ni
PPh$_2$Me
MePh$_2$P
Me
^{31}P NMR, 162 MHz
8.54
8.54
10.5 10.0 9.5 9.0 8.5 8.0 7.5 7.0 6.5 6.0
f1 (ppm)
140 120 100 80 60 40 20 0 -20 -40 -60 -80 -100 -120 -140 -160 -180 -200 -220 -240
f1 (ppm)

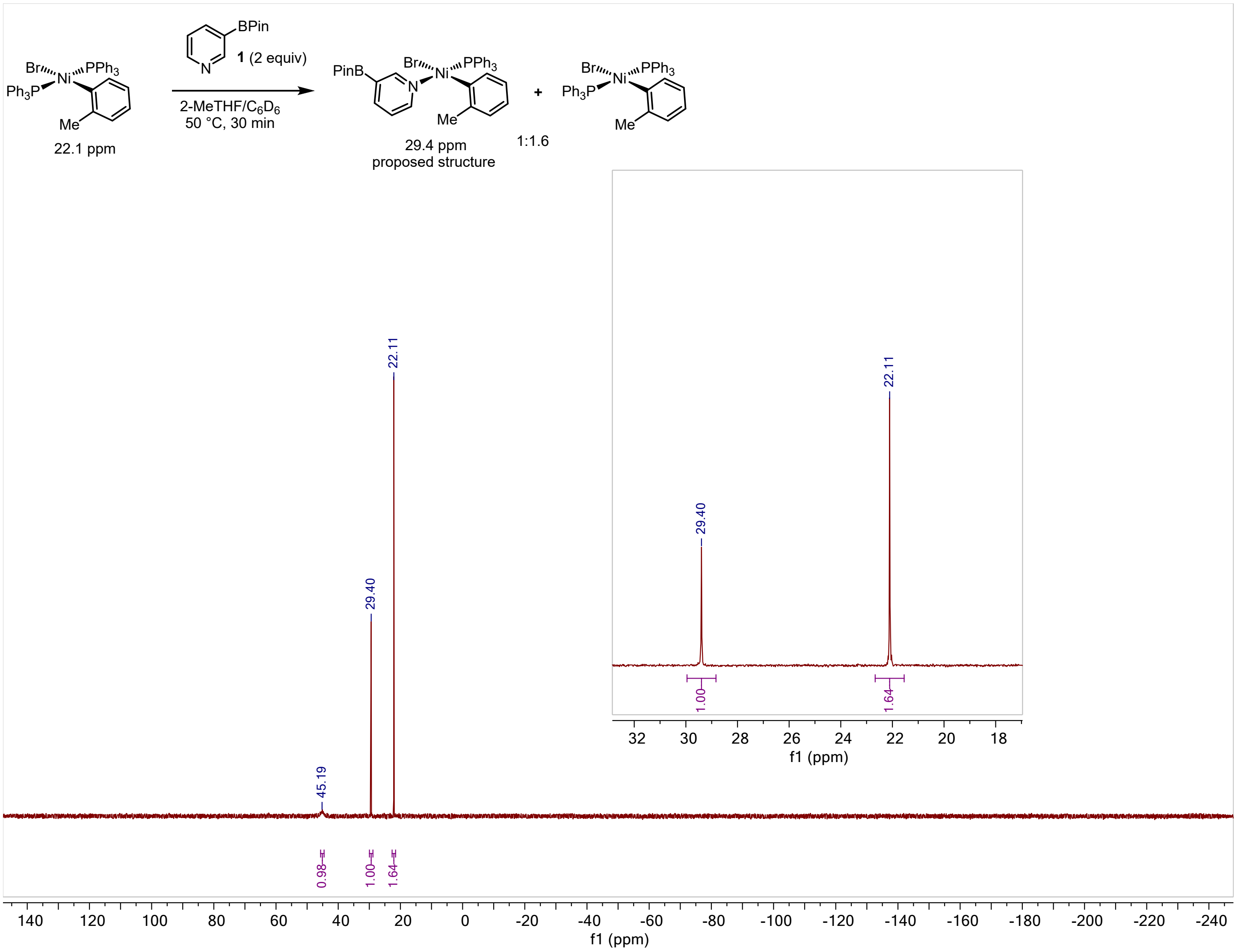

BPin
1 (2 equiv)
Br
PPh3
Ni
Ph3P
Me
22.1 ppm
2-MeTHF/C6D6
50 °C, 30 min
PinB
N
29.4 ppm
proposed structure
1:1.6
45.19
29.40
22.11
0.98
1.00
1.64
f1 (ppm)

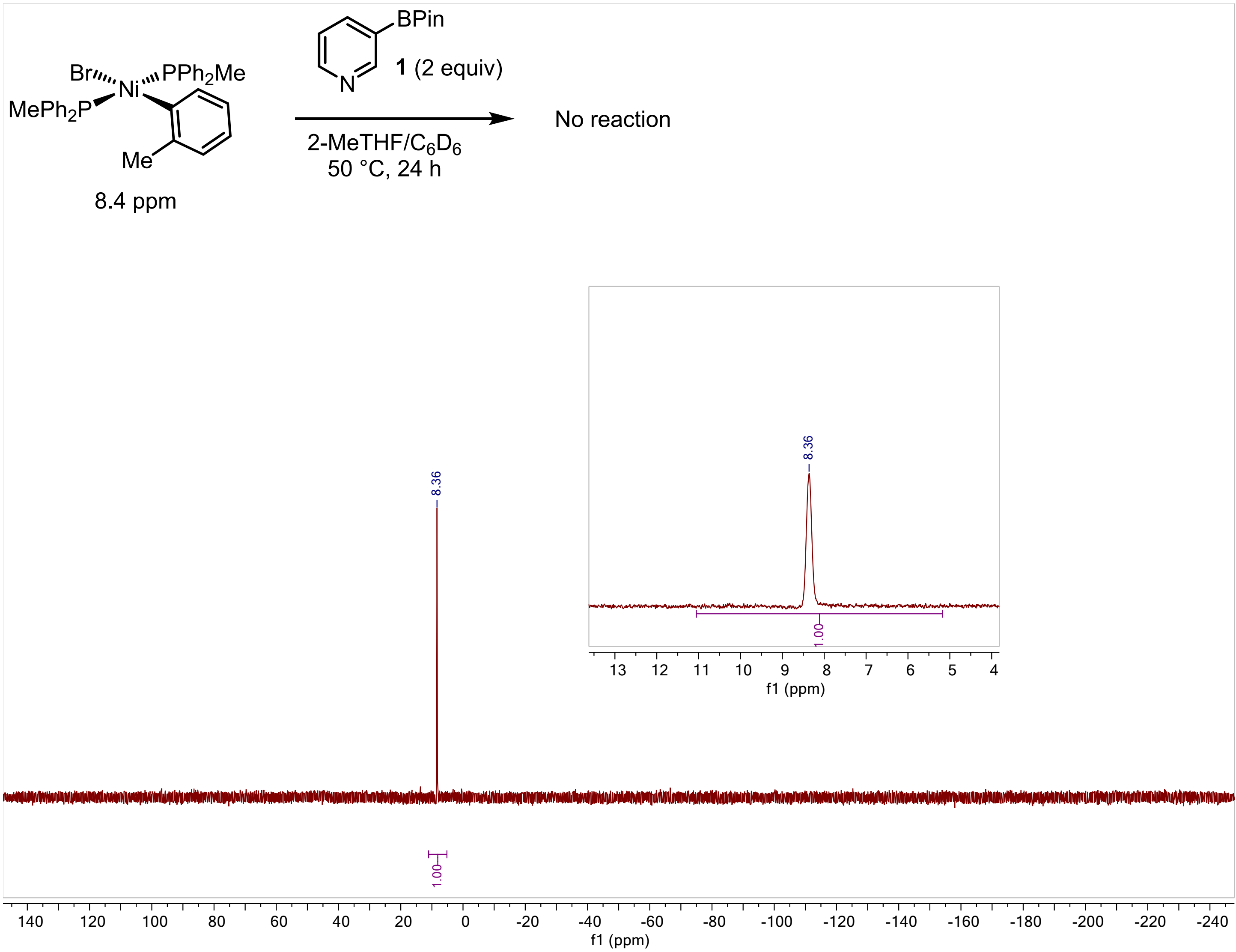
BPin
1 (2 equiv)
Br
Ni
PPh2Me
MePh2P
Me
8.4 ppm
2-MeTHF/C6D6
50 °C, 24 h
No reaction
8.36
1.00
f1 (ppm)

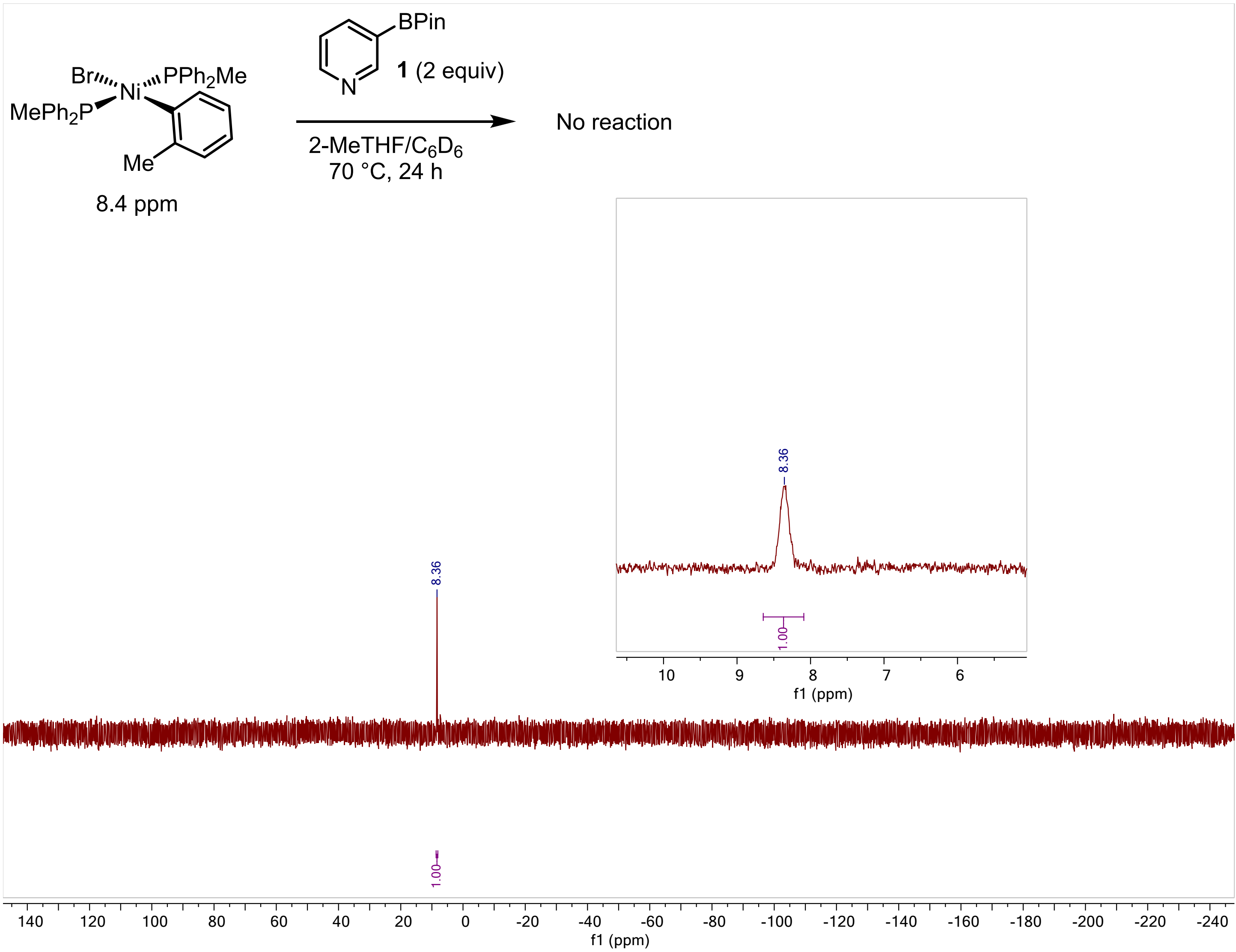
BPin
1 (2 equiv)
Br
Ni
PPh2Me
MePh2P
Me
8.4 ppm
2-MeTHF/C6D6
70 °C, 24 h
No reaction
8.36
1.00
f1 (ppm)
